# Multiplexed optical barcoding of cells *via* photochemical programming of bioorthogonal host–guest recognition†

**DOI:** 10.1039/d0sc06860h

**Published:** 2021-02-19

**Authors:** Arka Som, Meenakshi Pahwa, Sumit Bawari, Nilanjana Das Saha, Ranjan Sasmal, Monica Swetha Bosco, Jagannath Mondal, Sarit S. Agasti

**Affiliations:** New Chemistry Unit, Chemistry & Physics of Materials Unit, School of Advanced Materials (SAMat), Jawaharlal Nehru Centre for Advanced Scientific Research (JNCASR) Bangalore Karnataka 560064 India sagasti@jncasr.ac.in; Tata Institute of Fundamental Research 36/P, Gopanpally Village Hyderabad 500046 India

## Abstract

Modern chemical and biological studies are undergoing a paradigm shift, where understanding the fate of individual cells, in an apparently homogeneous population, is becoming increasingly important. This has inculcated a growing demand for developing strategies that label individual cells with unique fluorescent signatures or barcodes so that their spatiotemporal trajectories can be mapped in real time. Among various approaches, light-regulated methods employing photocaged fluorophores have received particular attention, owing to their fine spatiotemporal control over labelling. However, their multiplexed use to barcode large numbers of cells for interrogating cellular libraries or complex tissues remains inherently challenging, due to the lack of multiple spectrally distinct photoactivated states in the currently available photocaged fluorophores. We report here an alternative multiplexable strategy based on optically controlled host–guest recognition in the cucurbit[7]uril (CB[7]) system that provides spatial control over the positioning of fluorophores to generate distinct barcodes in ‘user-defined’ cells. Using a combination of three spectrally distinct CB[7]-conjugated fluorophores and by sequentially performing cycles of photoactivation and fluorophore encoding, we demonstrate 10-color barcoding in microtubule-targeted fixed cells as well as 7-color barcoding in cell surface glycan targeted live MCF7 cells.

## Abstract 2

Barcoding provides abilities to learn about individual species within an apparently homogeneous population. We describe a light-mediated multiplexed cellular barcoding strategy through spatial programming of cucurbit[7]uril molecular recognition.

## Graphical abstract

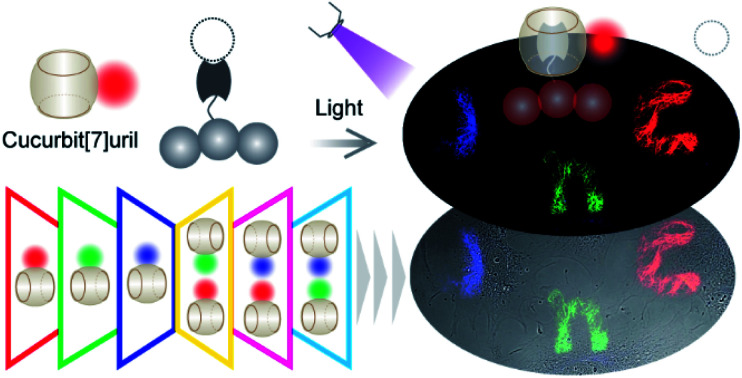

## Introduction

A central challenge in biology is to quantitatively understand how each cell processes information and spatiotemporally responds to environmental cues that ultimately produce complex and emergent functions or leads to a diseased state. However, much of our current knowledge is based on ensemble measurements where population averaging tends to obscure key insights from individual cells. For example, ensemble measurements can miss rare cell types and their function or completely overlook variability between cells and the functional significance of the heterogeneity.^1^ This highlights the growing importance of studying individual cells in complex environments and correlating their spatiotemporal dynamics with corresponding functional outcomes.^2^ Barcoding represents a pivotal strategy in this direction to enable single cell investigation across various domains of biological space.^3^ Barcoding is a technique in which selected members from a collection of identical species are labelled with unique recognizable signatures, termed as barcodes, so that their trajectories can be tracked individually through space and time.^4^ Among various possible barcodes, fluorescence-based marking offers significant advantages as it provides fast signaling, easy readout and real-time tracking with minimal disruption to the biological system.^5^ Particularly, in recent years light regulated fluorescence labeling methods have attracted increasing interest for this purpose, largely owing to their non-invasive nature and capability for fine spatial and temporal control over labelling.^6^

Traditionally light-regulated labeling requires specific fluorescent probes that are capable of exhibiting pronounced changes in their fluorescence emission in response to light stimulation. Towards this end, a class of photocaged fluorophores has been developed that has revolutionized the possibility of spatiotemporally controlled labeling.^7^ This specifically designed class of fluorophores remains in a non-fluorescent state prior to activation by light but switches over to a fluorescence ON state upon illumination at an activating wavelength of light, thereby providing a means to highlight a selected cell with spatiotemporal precision. In general, access to a range of photocaged fluorophores was achieved by synthetic modifications that either hinder their ability to absorb excitation light or control their nonradiative deactivation process.^6*d*,*f*,8^ However, an inherent challenge with the photocaged fluorophores is that they allow only single-color investigation, since photoactivatable fluorophores switch to a single fluorescent emissive state from an essentially non-emissive ‘OFF’ state upon light activation. A multicolor optical highlighting approach, generating a wide array of fluorescent profiles at spatiotemporal precision, can provide unique identification tags to many cells. This can be an extremely powerful tool for visualizing the dynamics of many cells within a given cellular population to address fundamentally different kinds of questions, including establishing interaction circuits between like cells and piecing together their lineage relationships over developmental, regeneration, or differentiation trajectories.^3^

A smaller subset of light-responsive probes, called photoconvertible probes, which are more abundant among the family of fluorescent proteins, have been developed for multicolor optical highlighting.^6*h*,9^ As a result of their ability to convert from one emissive state to another spectrally distinct emissive state upon light activation, these probes can improve multiplexing capability. While the photoconvertible probes based on proteins can be specifically expressed and possess distinct spectral features, the disadvantage of these probes, however, is that they are not suitable for continuous and long-term imaging as they are susceptible to photobleaching, and their color diminishes with protein turnover. On the other hand, the reports of synthetic fluorophores that display photoconvertible behavior are extremely rare.^10^ Optimizing the scaffold of fluorophores to achieve multiple distinguishable emission states from light-irradiation remains highly challenging and their ability to color-code many specifically targeted cells continues to be limited. These considerations prompted us to set a goal to develop a strategy that harnesses the benefits of light activation and concurrently provides scalable multiplexing with highly optimized synthetic fluorophores to enable barcoding of many cells in parallel.

**Scheme 1:**
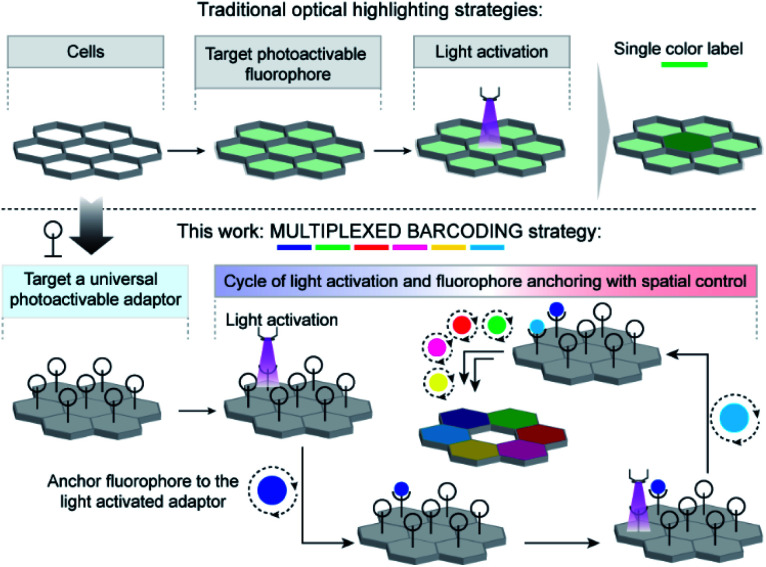
Strategy to achieve multiplexed barcoding of cells. In contrast to traditionally used photoactivatable fluorophores to label cells, we employed a universal photoactivatable adaptor to label cells. The universal adaptor allows spatial control of the positioning of fluorophores, where multiplexed barcoding can be readily achieved by performing sequential cycles of photoactivation and fluorophore coupling.

Here, to develop a light-mediated multiplexed barcoding strategy, rather than relying on the fluorophore's photophysical properties, we explored the possibility of spatially controlling the fluorophore's positioning by using a universal photoactivatable adaptor (Scheme 1). This universal adaptor can be specifically photoactivated to anchor any fluorophore or other user-defined color signatures in a spatially defined cell. We hypothesized that multiplexing would increase by sequentially performing a cascade of photoactivation and fluorophore coupling in different cells and by using a unique color signature for each cell. To design this universal adaptor, we considered bioorthogonal reactions, as they can be used to specifically couple synthetic reporters in live cells.^11^ The photoinducible version of certain bioorthogonal reactions has also been recently reported in the literature; however, there were a few challenges in adopting them in our sequential multiplexing scheme.^12^ They suffer from sluggish reaction kinetics, and their activation requires an extensive amount of light irradiation. These constraints typically push each photolabeling step to be completed in hour timescale, requiring many hours to completely label a cellular library *via* sequential activation. In addition, each step's inefficient coupling may result in extensive mixing of color signatures between two consecutive steps. Hence, a critical challenge for the success of our scheme was to achieve highly efficient bioorthogonal photolabeling with substantially minimized time of activation and conjugation in each cycle. We postulated that this can be achieved if we employ non-covalent bioorthogonal pairs for labeling as their association operates with diffusion-controlled kinetics (*k*_on_ ∼10^8^ M^−1^ s^−1^).^13^ However, to be suitable in our labeling scheme, we required non-covalent bioorthogonal pairs that not only display photoresponsive association, but also simultaneously provide high affinity and high selectivity recognition for efficient target imaging. To this end, we first developed a photoinducible bioorthogonal coupling strategy based on non-covalent synthetic host–guest recognition in the cucurbit[*n*]uril (CB[*n*]) family of synthetic hosts.^14^ Specifically, the heptameric member of this family, cucurbit[7]uril (CB[7]), has been the highlight in recent studies for its exceptional molecular recognition property in the biological milieu towards rationally designed guest molecules.^15^ One remarkable example is 1-adamantylamine (ADA), which displays high affinity (*K*_a_ > 10^12^ M^−1^) and highly specific molecular recognition towards CB[7] through a combination of hydrophobic, dipolar, and electrostatic interactions.^16^ To place CB[7] host–guest molecular recognition under optical control, we designed a photocaged ADA derivative (^*C*^ADA) based on theoretical insights from molecular dynamics simulation. The ^*C*^ADA was then used as a universal adaptor for spatially controlling the position of CB[7]-conjugated fluorophores (CB[7]-FL) in individual cells. Spatiotemporal control is first demonstrated in fixed cells by tethering ^*C*^ADA onto microtubule and actin filaments and subsequently labeling cytoskeletal filaments at sub-cellular resolution. Subsequently, utilizing a combination of three spectrally distinct CB[7]-FLs and combining it with the spatial encoding approach, we demonstrated 10-color barcoding in fixed cells. Finally, to show the photochemical encoding of barcodes in the living system, we paired light-mediated recognition with a metabolically incorporated reporter tag and showed 7-color barcoding in MCF7 cells.

## Results and discussion

**Fig. 1:**
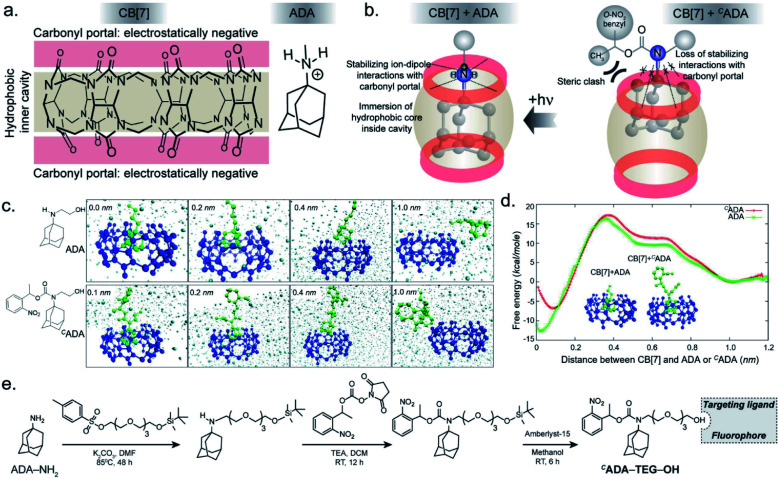
Design strategy for the photoresponsive CB[7] host–guest system and a theoretical insight on the association of the host and guest. (a) Chemical structure of the CB[7] host and the ADA guest. (b) In the high-affinity CB[7]–ADA inclusion complex, the hydrophobic core of the guest is immersed in the inner cavity while the outfitted cationic group is stabilized by the carbonyl portal. Attaching a suitable caging group with ADA restricts the formation of the inclusion complex due to the loss of stabilizing interactions and an increase in steric crowding around ADA. (c) Simulation snapshots at different binding distances between CB[7] and ADA or ^*C*^ADA. (d) Calculated free energy plot comparing the binding energies of CB[7] with ADA and ^*C*^ADA. (e) Synthesis scheme of ^*C*^ADA derivatives.

In the CB[7]–ADA host–guest complex, the hydrophobic core of the guest is preferentially immersed in the inner CB[7] cavity while the outfitted amine group is stabilized by ion–dipole interactions with the carbonyl portal (Fig. 1a). The synergistic interaction with the cavity and portal is the key trait to achieve high-affinity inclusion complex formation.^17^ In our molecular design of ^*C*^ADA, we thought to eliminate the impact of synergistic interplay that renders high-affinity binding between CB[7] and ADA by converting the amine residue of ADA to a carbamate functionality in ^*C*^ADA. This essentially blocks the ion–dipole stabilizing interaction with the carbonyl portal. Moreover, the formation of a bulky nitrogen center, with the large caging group in the vicinity, hinders the approach of the rigid CB[7] structure towards the hydrophobic ADA core of ^*C*^ADA, which additionally imposes a steric constraint for complex formation (Fig. 1b). In the case of activation, the carbamate functionality in ^*C*^ADA, being a part of the *o*-nitrobenzyl group, can be efficiently cleaved from the ADA core to generate the original amine moiety under single or two-photon illumination. This photocleavage eliminates the steric constraints as well as re-establishes the cooperative interaction with the carbonyl portal, thereby activating the high-affinity association between CB[7] and ADA. For theoretical insight, we performed molecular dynamics simulation studies, where we have calculated the free energy of association of CB[7] with ADA and ^*C*^ADA. We used the popular umbrella sampling method within the framework of classical molecular dynamics simulation for this purpose.^18^ This method introduces harmonic potentials (in addition to the molecular mechanical force field) across the range of the reaction coordinate, here taken as the distance between the center of mass (COM) between CB[7] and ADA or ^*C*^ADA. This approach makes sure that the free energy landscape is extensively sampled by the system and a quantitative measure of the free energy surface can be obtained. In the case of complexation between CB[7] and ADA, snapshots of the bound (0.0 nm) and unbound (1.0 nm) states are shown in Fig. 1c, along with snapshots at the barrier (0.4 nm) and partial binding (0.2 nm). The free energy landscape is also shown in Fig. 1d, which shows ∼12.77 kcal mol^−1^ free energy stabilization for complexation between CB[7] and ADA at a COM distance of ∼0.0 nm. However, for the interaction between CB[7] and ^*C*^ADA, the extent of free energy stabilization is much lower, and importantly the ^*C*^ADA molecule is observed to lie at a distance of ∼0.1 nm away from CB[7] (Fig. 1c and d). This suggests that while the ADA molecule is able to dock inside the cavity of CB[7], ^*C*^ADA is unable to access the hydrophobic cavity of CB[7]. Based on this theoretical support, we proceeded to synthesize various ^*C*^ADA derivatives, which were finally appended on fluorophores, proteins, and other cellular targeting motifs for barcoding studies (see ESI†).

**Fig. 2:**
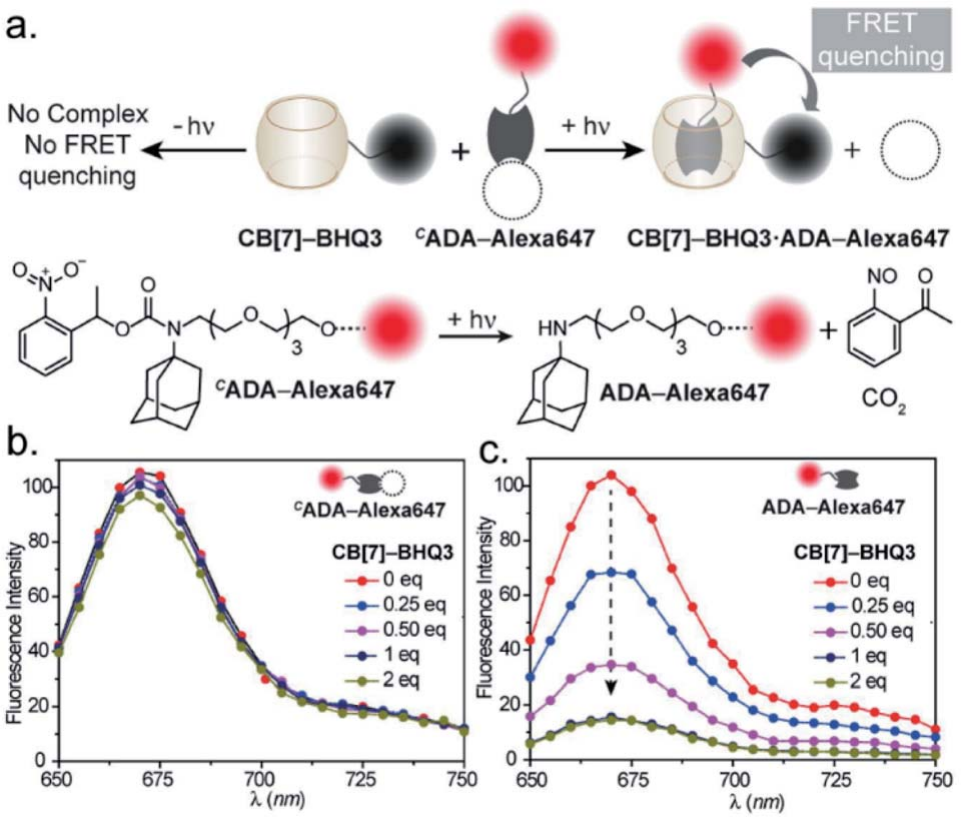
Binding studies using the FRET-based quenching assay. (a) Schematic representation of the assay. (b) Fluorescence titration of ^*C*^ADA-Alexa647 with the CB[7]–BHQ3 quencher, where increasing quencher concentration did not result in any significant fluorescence change. (c) Fluorescence titration of photoactivated ^*C*^ADA-Alexa647 (ADA-Alexa 647) with the CB[7]–BHQ3 quencher. Prominent changes in fluorescence intensity are observed with increasing quencher concentration and nearly quantitative FRET quenching at 1 equivalent of quencher concentration was detected from the titration.

We first used UV-vis absorption spectroscopy to analyze the time course of the photochemical reaction of the ^*C*^ADA (ESI Fig. S20†). Under 365 nm handheld LED light illumination ^*C*^ADA was completely cleaved within ∼90 s, suggesting highly efficient activation kinetics. Additionally, the presence of isosbestic points in the differential absorption profiles (*A*_*t*_–*A*_0_) indicated that the reaction proceeds without any undesired side products (ESI Fig. S21†). We also analyzed the photochemical reaction products of ^*C*^ADA derivatives by liquid chromatography-mass spectrometry (LCMS) analysis. Quantitative formation of desired photocleaved ADA products was observed from each ^*C*^ADA conjugate, indicating a clean and efficient photochemical transformation that is unperturbed by derivatization (ESI Fig. S22†). Upon confirming the photo uncaging characteristic of ^*C*^ADA, we designed a Förster resonance energy transfer (FRET)-based quenching experiment to investigate light-gated molecular recognition with the CB[7] host. For this experiment, ^*C*^ADA was derivatized with an Alexa Fluor 647 fluorescent dye (^*C*^ADA-Alexa 647), and CB[7] was covalently conjugated with a BHQ3 Quencher (CB[7]–BHQ3) (Fig. 2a). To estimate the amount of quenching, a 100 nM solution of ^*C*^ADA-Alexa 647 in PBS (pH = 7.4) was titrated against CB[7]–BHQ3. Alexa Fluor 647 fluorescence was recorded after each addition of CB[7]–BHQ3 aliquots, and the titration was conducted up to a total of 2 eq. of CB[7]–BHQ3 with respect to the concentration of ^*C*^ADA-Alexa 647. Notably, the emission spectra showed negligible fluorescence quenching throughout the course of titration (Fig. 2b), effectively demonstrating the lack of host–guest complex formation between ^*C*^ADA and CB[7]. However, when titration was performed with a ^*C*^ADA-Alexa 647 solution that was already irradiated with light, an immediate and near-complete fluorescence quenching was observed upon the addition of 1 eq. of CB[7]–BHQ3 (Fig. 2c). This indicates the quantitative formation of the CB[7]–ADA host–guest complex, a consequence that is well-aligned with the generation of high-affinity ADA-Alexa647 guests upon light irradiation. Moreover, a direct comparison of quenching efficiencies, before and after light irradiation, suggests that the system can completely transform from nearly ‘no complex’ to ‘∼100% complex’ with a pulse of light stimulus in a concentration range (low nM) that is well suited for biological studies. Further, we used microscale thermophoresis (MST) and NMR experiments to validate our findings, which also concluded a much-reduced affinity for ^*C*^ADA towards CB[7], which can be activated by light irradiation (ESI Fig. S23 and S24†).

**Fig. 3:**
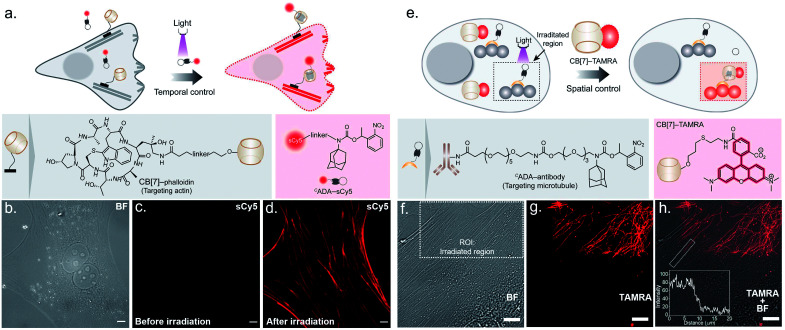
Photocontrolled host–guest molecular association in cells. (a) Schematic showing photochemical control over labeling of actin filaments using the ^*C*^ADA-sCy5 conjugate. (b) Brightfield image (BF) of MEF cells. (c) SIM image showing no visual actin localization of the sCy5 fluorophore before photoactivation. (d) SIM image after irradiation showing clear actin labeling in the sCy5 channel, mediated *via* activation of ^*C*^ADA and formation of the CB[7]–ADA-sCy5 complex. (e) Schematic showing spatial control over microtubule labeling using the ^*C*^ADA-antibody. (f) Brightfield (BF) image of MEF cells showing the region of interest (ROI, dotted rectangle) selected for localised photoirradiation. (g) CLSM image after photo-irradiation showing subcellularly localised microtubule labelling (shown in red) in the TAMRA channel. (h) Merged BF and CLSM image represent that the photocontrolled labelling of the microtubule is confined only in the defined ROI. The inset represents the fluorescence intensity profile along the activated microtubule. Scale bar: 10 μm.

To probe the feasibility of this CB[7] host–guest system to enable photocontrolled molecular associations in cellular complexities, we first performed a biomolecular labeling experiment in fixed cells under temporal light control. We utilized a CB[7] conjugated phalloidin derivative to target actin filaments in mouse embryonic fibroblast (MEF) cells (Fig. 3a). To this end, we employed sCy5 dye conjugated ^*C*^ADA as the fluorescent reporter probe to gain temporal control over the labeling process. Post CB[7]-phalloidin staining, the cells were incubated with a solution of ^*C*^ADA-sCy5 and the fluorescence image was subsequently acquired using structured illumination microscopy (SIM). As shown in Fig. 3c, the SIM image showed negligible fluorescence from the cells (3b, bright field image) and no apparent visualization of the actin filaments prior to illumination. Subsequently, the cells were irradiated with a hand-held 365 nm LED while ^*C*^ADA-sCy5 was still floating in the solution. Interestingly, SIM images acquired after the light pulse showed a fluorescently labelled actin network throughout the cellular structure (Fig. 3d), indicating *in situ* assembly of CB[7] decorated actin filaments with fluorescent probes. This result demonstrates that light-gated uncaging of the ADA moiety promotes its *in vivo* assembly with CB[7], resulting in stable and highly selective labeling of functional components in cells.

Next, we explored the capability of light-mediated molecular recognition to provide spatial control over the assembly process. Spatial confinement of the assembly instructions necessitates restriction over the free diffusion of the photoactivated component; hence an immobilization strategy for the ^*C*^ADA was devised. To this end, we have conjugated ^*C*^ADA with antibodies that ensure immobilization of the ^*C*^ADA on a specific target biomolecule (Fig. 3e). Attachment of the ^*C*^ADA with the secondary antibody against tubulin was verified by the mass shift in MALDI-MS measurement (ESI Fig. S25†). For the spatially confined labeling experiment, the tubulin cytoskeleton structure in MEF cells was decorated with ^*C*^ADA *via* a secondary immunostaining approach. Subsequently, a 100 nM solution of CB[7]–TAMRA was added onto the cells to visualize the location of the activated ADA labels. To activate the host–guest molecular recognition, the cells were placed under a Confocal Laser Scanning Microscope (CLSM), and a small square area of the visual window was illuminated by sequential line scanning with a 405 nm diode laser (Fig. 3f). As shown in Fig. 3g, TAMRA fluorescence was immediately observed from this area following a brief period of light illumination, indicating rapid formation of the CB[7]–ADA complex after activation of ^*C*^ADA. Fluorescence patterns resembling filamentous structures confirmed that the CB[7] was site-specifically assembled along the microtubule filaments. Importantly, when the photoactivated region (square area) was imaged under lower magnification and merged with a bright-field image (Fig. 3h), TAMRA labels appeared along the microtubule filaments only from a sub-cellular region; this demonstrates that the host–guest assembly was restricted to a spatially defined region. In addition, to quantitatively evaluate the performance of the system, we followed a single microtubule filament and drew an intensity profile along a 20 μm line (see the inset of Fig. 3h). Highly contrasting fluorescence intensity was observed between the illuminated and non-illuminated parts of the marked filament, indicating that the activation was achieved over a minimally emitting background, a feature that arises from the result of the minimum off-target assembly. To evaluate the stability of the fluorescence labels, we acquired time-lapse fluorescence microscopy images of the CB[7]–ADA mediated photo labeled cells for a period of >24 h. Quantitative analysis of the time-lapse fluorescence images showed a minimal change in the fluorescence intensity of the labeled cells over a 28 h period (ESI Fig. S26†), indicating negligible dissociation of the ADA-CB[7]–FL complex under the cellular environment and the suitability of this labeling strategy for monitoring cells over an extended period of time. Along with the high binding affinity of the CB[7]–ADA complex, we attribute this slow dissociation to additional factors, including secondary interactions of CB[7], post-CB[7]–ADA complex formation, with surrounding chemical moieties (*e.g.*, amino acid residues in a proximal protein) *via* the free carbonyl portal. In addition, the mass transport limitation in the crowded cellular environment can create a local retention zone for CB[7]-FL to allow dissociated CB[7]-FL molecules to rebind to an ADA site, contributing to the slow dissociation kinetics of the CB[7]–ADA complex. Next, we tested if this strategy can be expanded to other cytoskeleton proteins or even in tissues. In this direction, we have synthesized a ^*C*^ADA conjugated phalloidin derivative to label actin in a spatially controlled manner (ESI Fig. S27†). ESI Fig. S27† shows a fluorescence image, where actin staining in a photoactivated cell could be easily distinguished from the rest of the cells. Likewise, when targeted to the muscle associated actin structure in *Drosophila melanogaster*, we observed a light-mediated assembly in tissue with concurrent visualization of the specific actin patterns in muscle (ESI Fig. S28†). We also investigated whether the assembly process can be activated under two-photon illumination. To test this, we have illuminated a small intercellular volume of a ^*C*^ADA labeled cellular actin with a pulse of 725 nm laser light from a Ti-sapphire laser. To our delight, we observed a specific actin labeling *via* two-photon activation of the interaction between CB[7] and ADA (ESI Fig. S29†), demonstrating an attribute that can have far-reaching applications in studies *in vivo* and with animal models.

**Fig. 4:**
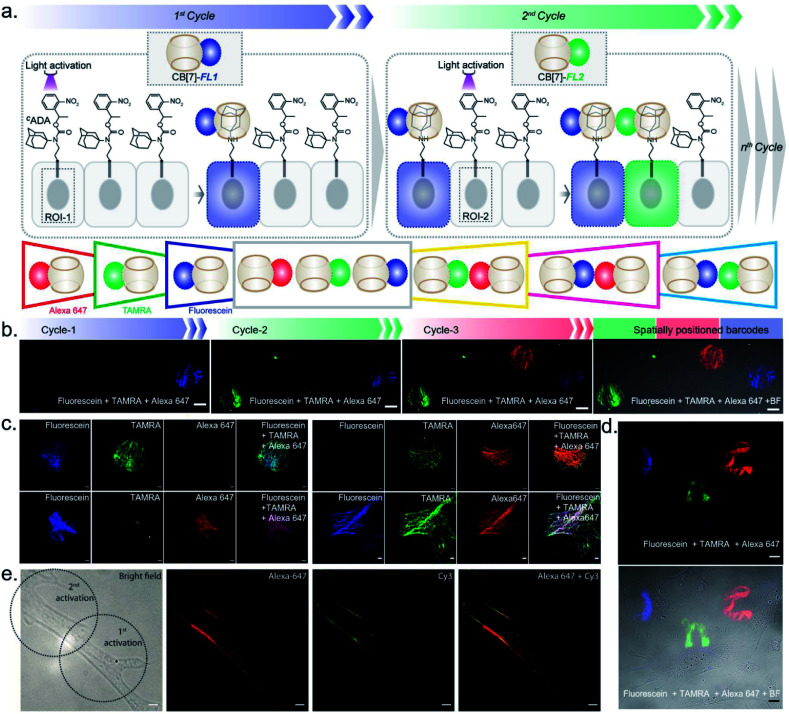
Multicolor barcoding of cells *via* photocontrolled host–guest association. (a) Schematic of the light-activated cellular barcoding strategy. Each cycle represents photoactivation followed by assembly with a unique color coded CB[7] host. (b) Three color barcoding of cells using three cycles of sequential photoactivation and fluorophore tagging. (c) Fluorescence images representing dual-color and triple-color barcoding that are achieved *via* additive combination of binary and ternary mixtures of CB[7] fluorophores in equal ratio. (d) Barcoding in the shape of J, n, and c with three cycles of fluorophore coding. (e) Dual color barcoding of a single actin filament within a cell. Scale bar: 20 μm (b), 10 μm (c), 20 μm (d), and 5 μm (e).

**Fig. 5:**
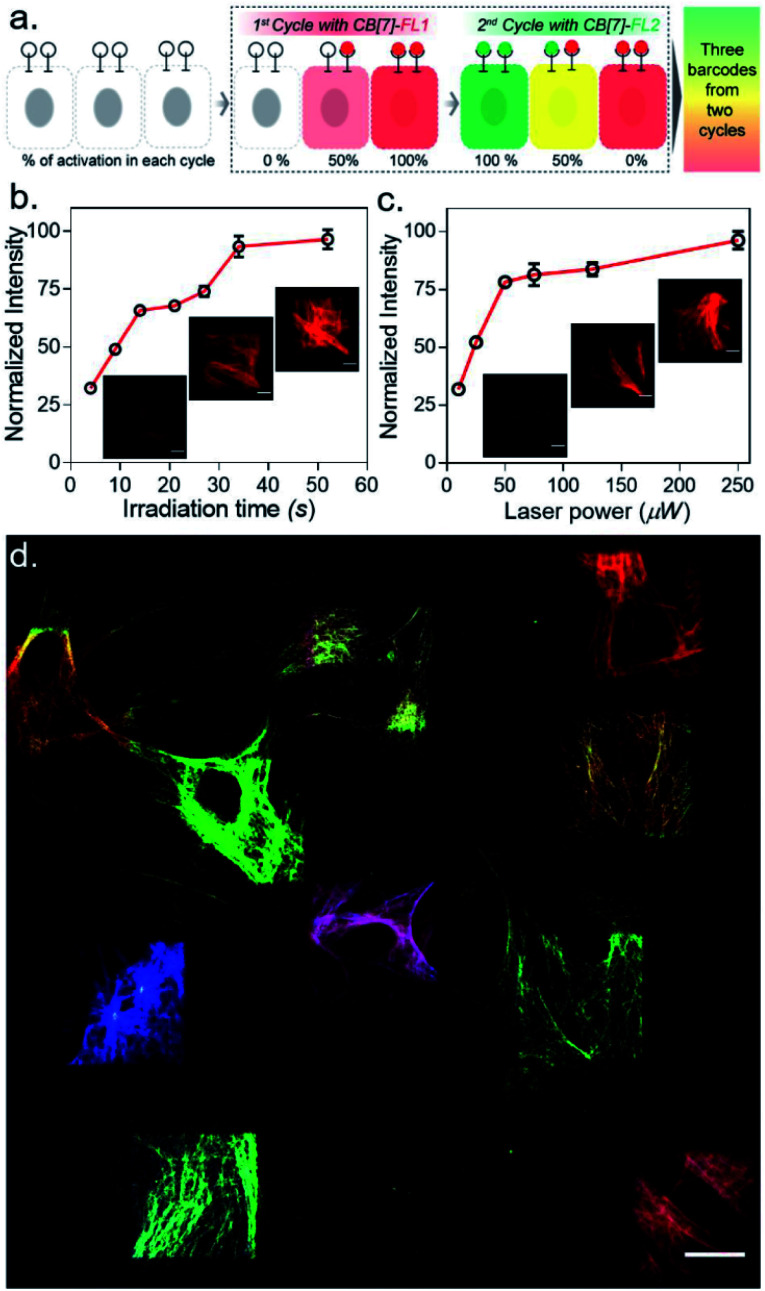
Rapid barcoding *via* secondary control over the degree of photoactivation. (a) Strategy showing the control over the degree of ^*C*^ADA photoactivation which allows 2 cycles to generate 3 barcodes in cells. (b and c) Control over the degree of ^*C*^ADA photoactivation/labeling *via* microscopic control over irradiation time (b) and laser power (c). (d) The 10-color barcodes that are generated in the cells by using 3 cycles and 3 CB[7]-FLs. Scale bar: 10 μm (b, c) and 20 μm (d).

After establishing spatially programmed assembly *via* optically controlled host–guest recognition, we proceeded to perform multiplexed barcoding of cells. We strategized that sequentially performing multiple rounds of photoactivation and CB[7]-FL assembly at ‘user-defined’ spatial coordinates, with a unique CB[7]-FL in each round, will graft recognizable optical signatures in individual cells (Fig. 4a). To test our hypothesis, we positioned our ^*C*^ADA adaptor in MEF cells by targeting α-tubulin using a ^*C*^ADA-conjugated antibody. During the 1st cycle, a defined spatial coordinate, called the region of interest (ROI), in a selected cell was photoactivated using a 405 nm laser to uncage the ^*C*^ADA molecule (Fig. 4a). It was followed by incubation with 100 nM CB[7]-fluorescein for 5 min. This allowed anchoring of the fluorescein signature over the first selected cell *via* CB[7]-fluorescein assembly with the activated ADA label. The cells were then washed to remove the excess fluorophore and imaged simultaneously in three fluorescence channels (*λ*_ex_: 488 nm, 561 nm, and 633 nm). The 1st cycle resulted in a fluorescence signal in the 488 nm channel and a negligible signal in other channels (Fig. 4b). The activation and anchoring cycle was repeated in two different cells using two other spectrally distinct CB[7]-FLs, CB[7]-TAMRA and CB[7]-Alexa 647. The image acquired after the 2nd cycle (using CB[7]-TAMRA) showed two optically barcoded cells with fluorescein and TAMRA signatures (Fig. 4b). Subsequently, the completion of the 3rd cycle (using CB[7]-Alexa 647) resulted in the appearance of three distinct optical signatures encoded in three different cells, fluorescein, TAMRA, and Alexa-647 (Fig. 4b). This result effectively demonstrates that ^*C*^ADA can act as a universal adaptor to incorporate unique optical signatures or barcodes in a number of selected cells. Further, barcoded cells were monitored for >24 h to evaluate any colour mixing between these cells over time. ESI Fig. S32† shows time-lapse fluorescence images of the barcoded cells with individual fluorophore channels as well as merged images. These images clearly depict no observable colour mixing over a 30 h time period, confirming negligible dissociation of the ADA–CB[7]-FL complex. In addition to using a single CB[7]-FL in each cycle, we further employed a mixture of CB[7]-FLs in each round, including binary (*e.g.*, 1 : 1, 4 : 1 and 1 : 4) and ternary mixtures (*e.g.*, 1 : 1 : 1) of CB[7]-FLs. This process generated a larger number of color palettes and enhanced the number of possible barcodes from this strategy (Fig. 4c and ESI S33†). We selected the ratio of CB[7]-FLs in such a way that a distinct difference could be observed between two barcodes upon analysis of the fluorescence images (ESI Fig. S34†). Having a large difference between these proportions would ensure an easy discrimination between the barcodes even while taking any experimental variations into account. Moreover, we demonstrated that optical encoding can be achieved in a user-defined shape, where we have written the letters ‘J’, ‘n’, and ‘c’ with three different CB[7]-FLs over the cells (Fig. 4d). We further explored if this barcoding strategy could be extended to the resolution of a single filament structure. For this purpose, we performed spatial color-coding of a single actin fiber inside a cell using two CB[7]-FLs, CB[7]-Alexa 647 and CB[7]-Cy3. As shown in Fig. 4e, super-resolution probing by SIM imaging showed color-coded blocks on a single fiber, demonstrating effective translation of the barcoding strategy to a single filament level, which could be a useful tool to control and study spatiotemporal changes at the sub-cellular level. To enhance the applicability of our barcoding strategy, we next explored whether we can barcode a large number of cells with a minimum number of activation and CB[7]-FL assembly cycles. In addition to spatial control, we strategized that a secondary control over the degree of ^*C*^ADA photoactivation will allow *n* number of cycles to generate ˃*n* number of unique barcodes in cells. For example, Fig. 5a demonstrates how 3-color barcodes can be generated by two activation cycles with two CB[7]-FLs. Here, the additional control over the degree of ^*C*^ADA photoactivation (in each cycle) provides a way to generate a unique color signature *via* controlled mixing of CB[7]-FLs. In this respect, we tested two microscope parameters to control the degree of ^*C*^ADA photoactivation: the activating laser power and the activation time. Selected ROIs in MEF cells, containing microtubule targeted ^*C*^ADA, were activated either by varying laser power or irradiation time and subsequently labeled with CB[7]-Alexa 647. Quantitative analysis of the microtubule fluorescence intensity from different ROIs demonstrated that the degree of labeling (∝^*C*^ADA activation) can be efficiently controlled by varying these two microscope parameters (Fig. 5b and c). Subsequently, we tried to integrate these secondary controls in our strategy to generate 10 different barcodes in 10 different MEF cells, using three activation cycles and three CB[7]-FLs. To implement this strategy, during every cycle, each of the selected ROIs in MEF cells was photoactivated by a specific activation power and irradiation time. The 1st activation cycle was followed by labeling with CB[7]-Alexa 647, whereas the 2nd and 3rd activation cycles were followed by labeling with CB[7]-fluorescein and CB[7]-TAMRA, respectively (ESI Scheme S9†). After completion of the 3rd cycle, fluorescence images of the cells were recorded in three emission channels. The final image (Fig. 5d) which is constructed by merging all the fluorescent channels clearly showed the presence of 10 unique color signatures (barcodes) in 10 spatially defined cellular ROIs. Each one of these barcodes is composed of a unique combination of three fluorophores, as evident from the analysis of their emission intensities (ESI Fig. S36a†). This result successfully establishes a simple strategy to quickly generate a large number of cellular barcodes by using a control over the degree of ^*C*^ADA photoactivation.

**Fig. 6:**
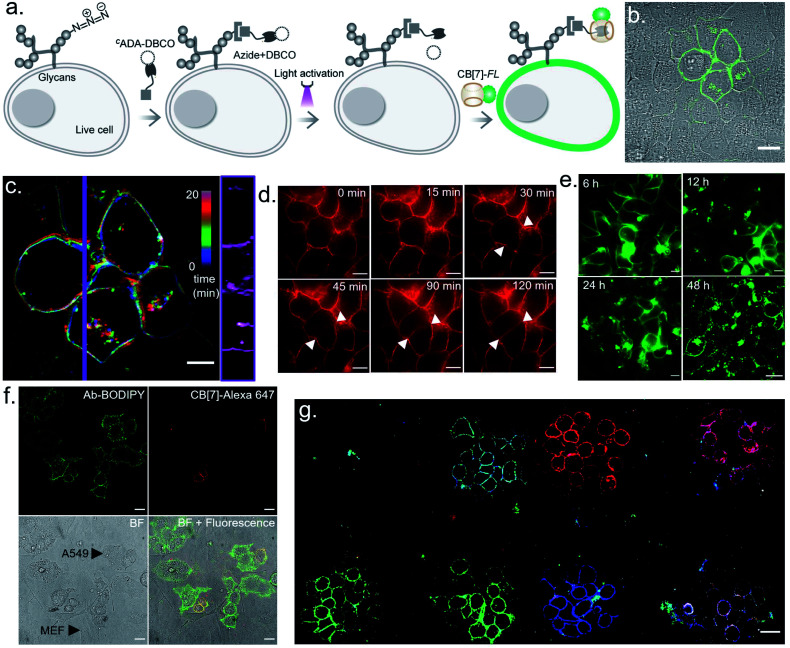
Multicolour barcoding of live cells *via* targeting cell surface glycans. (a) Schematic illustration of photoactivated labelling of glycans in live cells. Sialic acid bearing glycans are metabolically labelled with Ac_4_ManNAz and subsequently reacted with ^*C*^ADA-DBCO. (b) Optical labelling of glycans in HEK-293 cells using CB[7]-TAMRA. (c) Color-coded time lapse imaging of spatially labelled glycans over a time period of 20 min. Kymograph (right) constructed along the purple vertical line showing time-dependent changes in membrane reorganization. (d) Continuous monitoring of the labelled glycans in MCF7 cells over time shows that the host–guest probe is retained over the newly formed membrane of the daughter cells. (e) Long term monitoring of the glycan label in live cells demonstrates the existence of a fluorescence signal over a 48 h period. (f) Photoactivated labelling of EGFR overexpressing live A549 cells in a co-culture with fibroblast cells. Post BODIPY and ^*C*^ADA conjugated antibody staining of the EGFR receptor, A549 cells can be specifically identified *via* the presence of the fluorescence BODIPY signature (green) from their membrane. After photoactivation of two regions and assembly with CB[7]-Alexa 647, selected A549 cells are seen to be specifically stained with CB[7]-Alexa 647 dye (red). (g) CLSM image showing generation of 7-color barcodes in glycan labelled live MCF cells. Barcodes were generated in 3 cycles using 3 CB[7]-FLs. Please note that two fields of view were merged to generate this overall image. Scale bar: 20 μm (b), 10 μm (c, d), and 20 μm (e, f, g).

We next investigated the translation of this multiplexed barcoding strategy to live cell settings. Live cells require a significantly challenging setting for programming host–guest assembly instructions. Their complexity and dynamic environment demand a high level of specificity, affinity, and stability to facilitate a desired complexation between synthetic recognition partners at spatiotemporal resolution. Additionally, host and guest pairs should be programmable in a concentration range that imposes a minimal adverse effect on cell health. To investigate the applicability of the CB[7] based photo responsive system for multiplexed barcoding of live cells, we integrated ^*C*^ADA with cell surface glycans in HEK-293 and MCF7 cells. To target glycans in HEK-293 and MCF7 cells, we used a metabolic labelling strategy, where the bioorthogonal azide tag was incorporated into sialic acid bearing glycans by incubating cells with azide-modified mannosamine (Ac4ManNAz).^19^ Cells expressing azido glycans were reacted with a DBCO derivative of ^*C*^ADA *via* a strain promoted cycloaddition reaction to integrate ^*C*^ADA in the cell surface glycan structure (Fig. 6a). Cells were then incubated with CB[7]-TAMRA in phenol red-free culture media and placed under a microscope that is capable of maintaining precise and stable physiological conditions to maintain normal cell growth. Cells that were in the visual window of the microscope were activated with the 405 nm laser light. To understand the effect of activation, the fluorescence image of the cells in the TAMRA channel was acquired under lower magnification and merged with the bright-field image (Fig. 6b). The cell image revealed a strong TAMRA fluorescence signal emanating from the plasma membrane of the selected sub-population of activated HEK-293 cells. This confirms that CB[7]-TAMRA has been specifically assembled with membrane-associated glycans in live cells *via* photoactivation of ^*C*^ADA. The control experiment without the addition of ^*C*^ADA showed negligible fluorescence from the cell membrane, indicating specificity of the labeling process (Fig. S37†). Additionally, color-coded projection of time-lapse images shows the dynamic motion, growth and contraction of the cell membrane, indicating a minimal perturbation of the regular cellular physiology by the host–guest probes or from the light activation process (Fig. 6c). Importantly, temporal monitoring of the CB[7]-FL labelled glycans showed that our host–guest probe is retained over the newly formed membrane of the daughter cells along the cell division cycle (Fig. 6d). This not only confirms the stability of the CB[7]–ADA interaction in a living and dynamic environment but also indicates the possibility to track and monitor cellular progeny by this method. Moreover, we could find the fluorescence signals, emitting from the CB[7]-FL labelled cells, even after 48 h of labelling, potentially indicating long-term tracking prospects *via* this synthetic fluorophore based labelling strategy (Fig. 6e). Next, we demonstrated selective labelling of a specific cell type in the presence of other cells. For this purpose, we selected A549 lung carcinoma cells for photochemical host–guest labelling and co-cultured them with fibroblast cells. In the live co-culture, the A549 cells were specifically targeted using an antibody that binds with the overexpressed epidermal growth factor receptor (EGFR) on its cell surface. To specifically find the A549 cells for photolabeling in the live co-culture set-up with fibroblast cells, we attached a fluorescent dye (BODIPY) with the antibody along with the ^*C*^ADA derivative which is necessary for host–guest mediated photolabeling. As shown in Fig. 6f, post antibody staining, A549 cells can be specifically identified *via* the presence of the fluorescence BODIPY signature from their membrane. Subsequently, we selected two small regions for photolabeling *via* the CB[7]–ADA mediated interaction. After photoactivation and assembly with CB[7]-Alexa 647, we found that only photoactivated cells are specifically stained with Alexa 647 dye (Fig. 6f). This experiment demonstrates the possibility to find and selectively photolabel cancer cells in the presence of other cells. Finally, we explored the possibility to extend the multiplexed barcoding strategy to this live cell setting. Like the fixed cell barcoding, we selected seven different ROIs over MCF7 cells that were decorated with ^*C*^ADA *via* glycan attachment with the azide tag. The ROIs were activated by variable laser intensity in three sequential cycles. In each cycle, activated labels were tagged with spectrally distinguishable CB[7]-FLs, namely CB[7]-Alexa 647 (1st cycle), CB[7]-fluorescein (2nd cycle) and CB[7]-TAMRA (3rd cycle) (ESI Scheme S10†). Finally, ROIs were imaged in three different emission channels, corresponding to Alexa 647, fluorescein and TAMRA fluorophores. As shown in Fig. 6g and ESI Fig. S36b,† a merged image from these three different emission channels showed the presence of 7 different colour coded cells at spatially defined locations, demonstrating successful translation of the multiplexed barcoding strategy to live cell settings.

## Conclusion

In conclusion, we have developed a light-regulated cellular barcoding strategy based on photocontrolled host–guest molecular recognition in the CB[7] macrocycle. Rather than controlling the fluorophore's photophysical properties, we developed a distinct approach to barcode cells by spatially controlling the positioning of fluorescent labels. This represents a unique strategy that harnesses the benefits of light-regulation and concurrently provides highly multiplexed barcoding of living cells with highly optimized synthetic fluorophores. Our strategy builds upon the exceptional molecular recognition property of CB[7], where based on molecular dynamics simulation we designed and synthesized a photocaged guest molecule (^*C*^ADA) to place this molecular recognition under optical control. We utilized this optical control over the CB[7] host–guest recognition to spatially control the positioning of fluorophores to generate distinct barcodes in ‘user-defined’ cells. We optimized and validated this barcoding strategy in a fixed cell setting by specifically targeting the photoresponsive CB[7] host–guest pairs to microtubules. We subsequently showed the successful translation of this barcoding strategy to live cells by targeting cell surface glycans. In addition, we established an approach to rapidly create an enhanced number of distinct optical signatures or barcodes in cells by synergistically combining spatial encoding with microscopic control over the degree of ^*C*^ADA photoactivation. Given our strategy is independent of the types of labels used, we envision that our strategy can be easily synergized with other multiplexable labels, such as geometrically encoded fluorescent DNA-origami labels, to enhance the barcoding power of our strategy.^20^ Besides application in cellular barcoding, our photoresponsive CB[7] system benefits from the diffusion-controlled association kinetics and represents one of the fastest bioorthogonal coupling tools that can be manipulated by light. We expect that these fundamentally beneficial characteristics of this system will additionally enable a gamut of new explorations for light-gated bioorthogonal coupling strategies in basic and synthetic biology as well as in biomedical and imaging-related fields.

## Author contributions

A. S. and S. S. A. conceived the study, designed and performed the experiments, analysed and interpreted the data and wrote the manuscript. M. P. performed the binding experiments. S. B. and J. M. performed the simulation studies and wrote the manuscript. N. D. S. and M. S. B. prepared cells for the imaging experiment and reviewed the manuscript. R. S. helped with the imaging experiments. S. S. A. supervised the overall study.

## Conflicts of interest

There are no conflicts to declare.

## Supplementary Material

SC-012-D0SC06860H-s001

## References

[cit1] Walling M. A., Shepard J. R. E. (2011). Chem. Soc. Rev..

[cit2] Giedt R. J., Pathania D., Carlson J. C. T., McFarland P. J., del Castillo A. F., Juric D., Weissleder R. (2018). Nat. Commun..

[cit3] Kebschull J. M., Zador A. M. (2018). Nat. Methods.

[cit4] Gorris H. H., Wolfbeis O. S. (2013). Angew. Chem., Int. Ed..

[cit5] Chen C., Ao L., Wu Y.-T., Cifliku V., Cardoso Dos Santos M., Bourrier E., Delbianco M., Parker D., Zwier J. M., Huang L., Hildebrandt N. (2018). Angew. Chem., Int. Ed..

[cit6] Yu N., Huang L., Zhou Y., Xue T., Chen Z., Han G. (2019). Adv. Healthcare Mater..

[cit7] Zhao Y. R., Zheng Q., Dakin K., Xu K., Martinez M. L., Li W. H. (2004). J. Am. Chem. Soc..

[cit8] Li H., Vaughan J. C. (2018). Chem. Rev..

[cit9] Gurskaya N. G., Verkhusha V. V., Shcheglov A. S., Staroverov D. B., Chepurnykh T. V., Fradkov A. F., Lukyanov S., Lukyanov K. A. (2006). Nat. Biotechnol..

[cit10] Tang S., Zhang Y., Dhakal P., Ravelo L., Anderson C. L., Collins K. M., Raymo F. M. (2018). J. Am. Chem. Soc..

[cit11] Devaraj N. K., Weissleder R. (2011). Acc. Chem. Res..

[cit12] V Mayer S., Murnauer A., von Wrisberg M.-K., Jokisch M.-L., Lang K. (2019). Angew. Chem., Int. Ed..

[cit13] Tang H., Fuentealba D., Ko Y. H., Selvapalam N., Kim K., Bohne C. (2011). J. Am. Chem. Soc..

[cit14] Assaf K. I., Nau W. M. (2015). Chem. Soc. Rev..

[cit15] Cao L., Šekutor M., Zavalij P. Y., Mlinarić-Majerski K., Glaser R., Isaacs L. (2014). Angew. Chem., Int. Ed..

[cit16] Liu S., Ruspic C., Mukhopadhyay P., Chakrabarti S., Zavalij P. Y., Isaacs L. (2005). J. Am. Chem. Soc..

[cit17] Ghale G., Ramalingam V., Urbach A. R., Nau W. M. (2011). J. Am. Chem. Soc..

[cit18] Kumar S., Rosenberg J. M., Bouzida D., Swendsen R. H., Kollman P. A. (1992). J. Comput. Chem..

[cit19] V Chang P., Prescher J. A., Sletten E. M., Baskin J. M., Miller I. A., Agard N. J., Lo A., Bertozzi C. R. (2010). Proc. Natl. Acad. Sci. U. S. A..

[cit20] Lin C., Jungmann R., Leifer A. M., Li C., Levner D., Church G. M., Shih W. M., Yin P. (2012). Nat. Chem..

