# Supplementary material for: Multiplexed optical barcoding of cells via photochemical programming of bioorthogonal host–guest recognition
Source: Chem Sci. 2021 Feb 19;12(15):5484–94. doi: 10.1039/d0sc06860h (PMC8179588; doi:10.1039/d0sc06860h)
Supplement: SC-012-D0SC06860H-s001 [file SC-012-D0SC06860H-s001.pdf]

Electronic Supplementary Material (ESI) for Chemical Science.
This journal is © The Royal Society of Chemistry 2021

Supporting Information

Multiplexed Optical Barcoding of Cells via Photochemical Programming of Bioorthogonal Host-Guest Recognition

Arka Som[1], Meenakshi Pahwa[1], Sumit.Bawari[2], Nilanjana Das Saha[1], Ranjan Sasmal[1], Monica Swetha Bosco[1], Jagannath Mondal[2], and Sarit S. Agasti[1*]

[1]New Chemistry Unit, Jawaharlal Nehru Centre for Advanced Scientific Research (JNCASR), Bangalore, Karnataka 560064, India. Chemistry & Physics of Materials Unit, Jawaharlal Nehru Centre for Advanced Scientific Research (JNCASR), School of Advanced Materials" (SAMat), Jawaharlal Nehru Centre for Advanced Scientific Research (JNCASR), Bangalore, Karnataka 560064, India.

[2] Tata Institute of Fundamental Research, 36/P, Gopanpally Village, Hyderabad 500046.

*Correspondence e-mail: sagasti@jncasr.ac.in

1. General materials and methods

All chemicals were purchased from either of the following company: Merck-Sigma Aldrich, Alfa Aesar, Fischer Scientific, TCI chemicals, SD fine chemicals and Spectrochem, unless mentioned specifically. Amino phalloidin was purchased from American Peptide Company (Product No. 92-1-10). ZebaTM spin desalting columns were purchased from Thermo Fisher Scientific. Antibodies and dyes were purchased from commercial sources as listed below. Whenever necessary, solvents were dried by using standard solvent drying methods and used for reactions. The compound yields reported here refer to that of the spectroscopically pure compounds upon purification.

^{1}H NMR spectra were recorded using Bruker ADVANCE III 400 MHz instrument and JEOL 600 MHz instrument. Data analysis was done using TopSpin 3.5pl7, SpinWorks 4 and JEOL Delta v5.0.5.1. High Resolution Mass Spectrometry (HRMS) was carried out using Agilent 6538 Ultra High Definition (UHD) Accurate-Mass Q-TOF LC/MS. Liquid Chromatography Mass Spectrometry (LCMS) was carried out using Waters e2695 instrument. High Performance Liquid Chromatography (HPLC) purification was carried out using Agilent 1260 infinity quaternary HPLC system equipped with analytical ZORBAX Eclipse plus C18 column (4.6 mm × 100 mm, 3.5 micron). The solvents, used as eluent in HPLC purification, were solvent A (water containing 0.1% TFA) and solvent B (acetonitrile containing 0.1% TFA). Absorbance measurement for photochemical study was carried out using Perkin Elmer Lambda 900 UV-Vis-NIR Spectrometer. The concentration of fluorophores/quenchers was checked in an Eppendorf BioSpectrometer. *In vitro* light activation was performed using a Thorlabs supplied 365 nm UV LED System (CS2010). Fluorescence measurement was carried using a Perkin Elmer LS-55 Luminescence Spectrometer**.** Fluorescence quenching and FRET experiments were carried out in a microplate reader (Synergy H1, BioTek). Microscale thermophoresis was carried out in Monolith NT.115 instrument. Fluorescence imaging using Structured Illumination Microscopy (SIM) was carried out using an inverted Zeiss ELYRA PS1 microscope. Zeiss LSM 880 and Leica TCS SP8 was used for confocal imaging. Zeiss LSM 510 META with Tsunami Spectra-Physics Millennia Pro (50 mW Pump-diode) was used for two-photon activation experiment. Widefield fluorescence imaging was carried out using Olympus inverted fluorescence microscope.

Supporting Table 1: Primary antibody used for immunostaining.

Target	Species	Antibody commercial sources
Microtubule (α-tubulin)	Rat	Thermo Fisher Scientific (MAI-80017)
Human EGFR (Cetuximab) MAb (Clone Hu1)	Human	R&D Systems, Inc (MAB9577-100)

Supporting Table 2: List of secondary antibodies used for immunostaining.

Target	Host	Specification and commercial source
Rat	Donkey	Donkey Anti-Rat IgG (H+L) (min X Bov, Ck, Gt, GP, SyHms, Hrs, Hu, Ms, Rb, Shp Sr Prot) Jackson ImmunoResearch Laboratories (Cat. No. 712-005-153)
Human	Donkey	Dockey Anti-Human IgG (H+L) (min X Bov, Ck, Gt, GP, SyHms, Hrs, Ms, Rb, Rat Shp Sr Prot) Jackson ImmunoResearch Laboratories (Cat. No. 709-005-149)

Supporting Table 3: List of fluorophores/quenchers used.

Fluorophores/Quenchers used	Commercial Source
Fluorescein Isothiocyanate	TCI (F0784)
TAMRA NHS ester	ANASPEC (AS-81124-01)
Cy3 NHS ester	Lumiprobe (Cat. No. 21020)
Sulfo-Cyanine5 NHS ester	Lumiprobe (Cat. No. 23320)
Alexa Fluor 647 NHS ester	Thermo Fisher Scientific (A20006)
BHQ-3 Succinimidyl Ester	Biosearch Technologies (BHQ-3000S-5)
BODIPY NHS Ester	TOCRIS bioscience (Cat. No. 5465)

2. Synthesis

2.1. Synthesis of ^{C}ADA-NHS ester derivative:

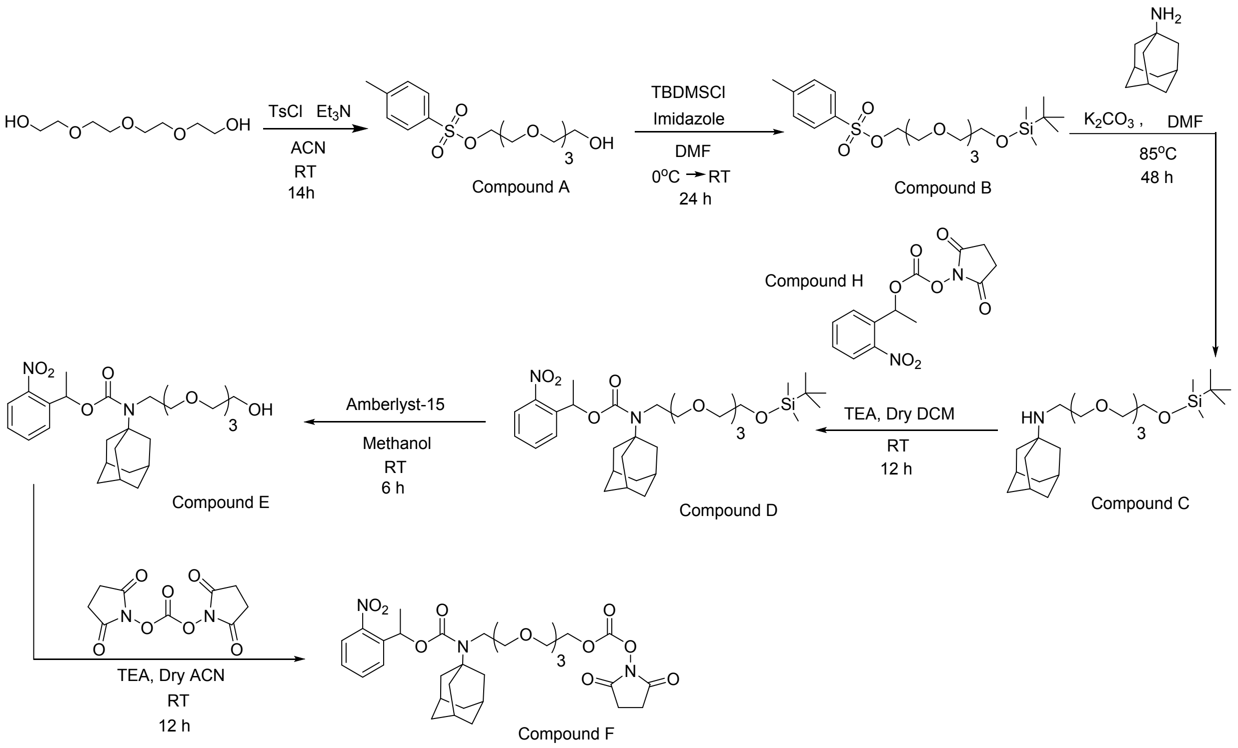

Scheme S1. Synthetic scheme for the preparation of ^{C}ADA-NHS-ester group (Compound F, ^{C}ADA–TEG–NHS-A).

Synthesis of compound A: Acetonitrile (130 mL) and triethyl amine (7.18 mL, 51.48 mmol) was added to a 250 mL round bottom (RB) flask containing tetra ethylene glycol (TEG, 10.00 g, 51.48 mmol). Tosyl chloride (9.76 g, 51.48 mmol), dissolved in 20 mL acetonitrile was added drop wise from a dropping funnel for 1 h while keeping the reaction mixture at 0°C. After the addition, the reaction mixture was stirred at room temperature for 14 h. During the reaction, a white precipitate of triethyl ammonium hydrochloride was formed. After completion of reaction, the precipitate was filtered and washed with acetonitrile. The acetonitrile was evaporated, and the compound was purified using flash silica (230-400 mess) column (eluent: ethyl acetate (EtOAc)/hexane, gradient elution from 0 to 80%). Compound A was obtained as colourless oil (7.510 g, 41.75% yield). R_f = 0.375 (EtOAc/hexane: 7:3). HRMS: (ESI-MS): calculated m/z 349.1316 $[M + H]^+$, found 349.1318 $[M + H]^+$. ^{1}H NMR (400 MHz, $CDCl_3$): δ 7.79(2H, d, ArH), 7.33(2H, d, ArH), 4.16 (2H, t, $-CH_2OSO_2-$), 3.69-

3.59 (14H, m, −OCH_2CH_2O−), 2.44 (3H, s, −CH_3). ^{1}H NMR spectra of compound A has been shown in Figure S1. ^{13}C NMR (400 MHz, $CDCl_3$): 144.85, 133.17, 129.84, 128.12, 72.52-70.76, 69.28, 68.76, 61.79, 21.61. ^{13}C NMR spectra of compound A has been shown in Figure S2.

Synthesis of compound B: Compound A (TEG-OTs, 7.51g, 21.57 mmol) was dissolved in 50 mL dry DMF in a 250 mL RB flask. Immidazole (1.47 g, 21.57 mmol) was added to the reaction mixture at 0°C followed by the addition of tert-Butyldimethylsilyl chloride (TBDMSCl, 3.25 g, 21.57 mmol). The reaction mixture was stirred for 24 h at room temperature. Upon completion of the reaction (monitored by TLC), the reaction mixture was diluted with hexane/H_2O (1:1 v/v) and the organic phase was extracted using EtOAc, the combined layer was dried over Na_2SO_4 and volatiles were removed using rota-evaporator. The crude product was purified by silica column (230-400 mess) chromatography (EtOAc/hexane) to yield compound B as a colourless viscous liquid (3.58 g, 42%). R_f = 0.60 (EtOAc/hexane: 7:3). HRMS**:** (ESI-MS): calculated m/z 480.2446 $[M +NH_4]^+$, found 480.2454 $[M + NH_4]^+$. ^{1}H NMR (400 MHz, $CDCl_3$): δ 7.79(2H, d, ArH), 7.33(2H, d, ArH), 4.16 (2H, t, −CH_2OSO_2−), 3.75-3.58 (14H, m, −OCH_2CH_2O−), 2.44 (3H, s, −CH_3), 0.88 (9H, µs, −$C(CH_3)_3$), 0.06 (6H, s, −$Si(CH_3)_2$) . ^{1}H NMR spectra of compound B has been shown in Figure S3. ^{13}C NMR (400 MHz, $CDCl_3$): 144.76, 133.14, 129.77, 127.99, 72.69-70.63, 69.22, 68.75, 62.76, 25.92, 21.61, 18.42, -5.27. ^{13}C NMR spectra of compound B has been shown in Figure S4.

Synthesis of compound C: 1-Aminoadamantane hydrochloride (1.45g, 7.73 mmol) and potassium carbonate (K_2CO_3, 2.67 g, 19.32 mmol) was taken in 250 mL RB flask. 12 mL of N, N-Dimethylformamide was added and stirred for 5 minutes. TBDMS-TEG-OTs (Compound B, 3.58 g, 8.62 mmole) was then added to the reaction mixture and stirred at 85°C for 48 h. Upon completion of the reaction (monitored by TLC), the reaction mixture was diluted with 20 mL water and extracted with dichloromethane (DCM) (3 × 40 mL). The combined organic layers were dried over Na_2SO_4 and solvent was removed under vacuum to give a white viscous liquid. The crude product was purified by silica column (230-400 mess) chromatography (eluent: DCM/Methanol (9:1)) to yield compound C as a yellow viscous liquid (1.10 g, 32.2%). R_f = 0.48 (DCM/Methanol: 9:1). HRMS**:** (ESI-MS): calculated m/z 442.3347 $[M +H]^+$, found 442.3349 $[M + H]^+$. ^{1}H NMR (400 MHz, $CDCl_3$): δ 3.76-3.54 (14H, m, −OCH_2CH_2O−), 2.82 (2H, t, −OCH_2CH_2NH−), 2.07 (3H, s, −CH), 1.69-1.62 (12H, s, −CH_2), 0.88 (9H, s, −$C(CH_3)_3$), 0.06 (6H, s, −$Si(CH_3)_2$) .^{1}H NMR spectra of compound C

has been shown in Figure S5. ^{13}C NMR (400 MHz, $CDCl_3$): 72.71-70.63, 62.76, 51.29, 42.07, 39.90, 36.62, 29.54, 25.96, 18.42, -5.24. ^{13}C NMR spectra of compound C has been shown in Figure S6.

Synthesis of compound D: Compound C (0.65 g, 1.39 mmol) was taken in 25 mL pear shaped flask under argon inert atmosphere at room temperature. 3.0 mL of dry dichloromethane and triethylamine (0.60 mL, 4.17 mmol) were added in a sequential manner. The mixture was kept under stirring and PC-ONHS (Compound H, 0.86 g, 2.79 mmol) was added to it and kept for 12 h at room temperature. Upon completion of the reaction (monitored by TLC), the reaction mixture was diluted with 10 mL water and extracted with dichloromethane (DCM) (3 × 20 mL). The combined organic layers were dried over Na_2SO_4 and solvent was removed under vacuum to give a yellow oil. The crude product was purified by silica column (230-400 mess) chromatography (eluent: Hexane/EtOAc 2:1→1:1) to yield compound D as a yellow viscous liquid (0.50 g, 56.1 %). R_f = 0.80 (Hexane/EtOAc: 1:1). ^{1}H NMR (400 MHz, $CDCl_3$): δ 7.94 (1H, d, ArH), 7.62 (2H, d, ArH), 7.40 (1H, m, ArH), 6.28 (1H, q, −CH), 3.76-3.52 (16H, m, −OCH2CH2O−), 2.09 (9H, s, −CH, −CH2), 1.63 (9H, m, −CH3, −CH2), 0.89 (9H, s, −C(CH3)3), 0.06 (6H, s, −Si(CH3)2). ^{1}H NMR spectra of compound D has been shown in Figure S7.

Synthesis of compound E: Compound D (0.50 g, 0.79 mmol) was taken in 10 mL pear shaped flask and dissolved in 3.0 mL of methanol. Amberlyst-15 (0.16 g, 0.2 g/mmol) was then added and stirred for 6 h at room temperature. Upon completion of reaction (monitored by TLC), the reaction mixture was filtered to separate out amberlyst beads and purified by silica column (230-400 mess) chromatography (eluent: EtOAc/Hexane 1:1) to yield compound E as a yellow viscous liquid (0.32 g, 32.2%). R_f = 0.66 (100% EtOAc). HRMS**:** (ESI-MS): calculated m/z 521.2857 $[M +H]^+$, found 521.2858 $[M + H]^+$. ^{1}H NMR (400 MHz, $CDCl_3$): δ 7.94 (1H, d, ArH), 7.62 (2H, d, ArH), 7.40 (1H, m, ArH), 6.28 (1H, q, −CH), 3.76-3.52 (16H, m, −OCH2CH2O−), 2.09 (9H, s, −CH, −CH2), 1.64 (9H, m, −CH3, −CH2). ^{1}H NMR spectra of compound E has been shown in Figure S8. ^{13}C NMR (400 MHz, $CDCl_3$): 154.24, 147.51, 139.28, 133.44, 127.96, 127.27, 124.58, 72.53-70.58, 68.82, 61.78, 57.20, 42.61, 40.60, 36.36, 30.21, 22.46. ^{13}C NMR spectra of compound E has been shown in Figure S9.

Synthesis of compound F (*^{C}*ADA–TEG–NHS-A): Compound E (30.0 mg, 0.06 mmol) was taken in 10 mL pear shaped flask under argon inert atmosphere at room temperature. 1 mL of

dry acetonitrile and triethylamine (48.23 µL, 0.35 mmol) was added dropwise in a sequential manner. N, N′-Disuccinimidyl carbonate was dissolved in 1 mL of dry acetonitrile which was added to the reaction mixture and stirred for 12 h at room temperature. Upon completion of the reaction (monitored by TLC), the reaction mixture was immediately evaporated to dryness using rota-vapour .The crude product was purified by silica column (230-400 mess) chromatography (eluent: Hexane/EtOAc 4:1→1:4) to yield compound F as a yellow viscous liquid (17.3 mg, 45.4%). R_f = 0.52 (Hexane/EtOAc: 1:4). HRMS: (ESI-MS): calculated m/z 662.2920 $[M+ H]^+$; found 662.2895 $[M+H]^+$. ^1H NMR (400 MHz, $CDCl_3$): δ 7.96 (1H, d, ArH), 7.62 (2H, d, ArH), 7.41 (1H, m, ArH), 6.28 (1H, q, −CH), 4.45 (2H, t, −OCH2CH2O−), 3.78-3.53 (14H, m, −OCH2CH2O−), 2.83 (4H, s, −CH2), 2.09 (9H, s, −CH, −CH2), 1.64 (9H, m, −CH3, −CH2). ^1H NMR spectra of compound F has been shown in Figure S10.

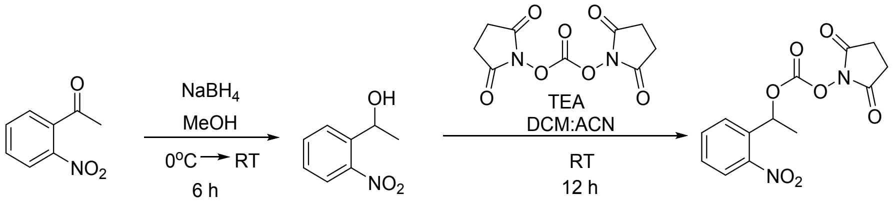

Compound G

Compound H

Scheme S2. Synthetic scheme for the preparation of photocleavable group (Compound H)

Synthesis of compound G: 2′-Nitroacetophenone (1.00 g, 6.05 mmol) was taken in a 100 mL RB flask with 15 mL methanol. Sodium borohydrate (0.92 g, 24.20 mmol) was added to the mixture in parts at 0°C under stirring. After stirring for 6 h at room temperature, the reaction mixture was monitored by TLC. Upon completion of the reaction the solution was evaporated to dryness using rota-vapour and diluted with 20 mL water and extracted with ethyl acetate (EtOAc) (3 × 40 mL). The combined organic layers were dried over Na_2SO_4 and solvent was removed under vacuum to give a yellow liquid (0.75 g, 74.09%). The crude product was used for next step without further purification. R_f = 0.24 (Hexane/EtOAc: 5:1). ^1H NMR (400 MHz, $CDCl_3$): δ 7.90 (1H, d, ArH), 7.83 (1H, d, ArH), 7.65 (1H, t, ArH), 7.41 (1H, t, ArH), 5.43 (1H, q, −CH), 2.30 (1H, s, OH), 1.57 (3H, d, $-CH_3$). ^1H NMR spectra of compound G has been shown in Figure S11.

Synthesis of compound H: Compound G (0.75 g, 4.49 mmol) was dissolved in 5 mL of dry acetonitrile and dichloromethane (1:1) in a 100 mL RB flask under nitrogen inert atmosphere. Triethylamine (TEA, 1.88 mL, 13.47 mmol) and disuccinimidyl carbonate (DSC, 3.45 g,

13.47 mmol) was added in a sequential manner and stirred for 12 h at room temperature. Upon completion of the reaction, (monitored by TLC), the reaction mixture was immediately evaporated to dryness using rota-vapour, diluted with 20 mL water and extracted with dichloromethane (DCM) (3 × 40 mL). The combined organic layers were dried over Na_2SO_4 and solvent was removed under vacuum to give a yellowish solid. The crude product was purified by silica column (230-400 mess) chromatography (eluent: Hexane/EtOAc 4:1→1:1) to yield compound H as a white solid (0.94 g, 67.96%). $R_f = 0.52$ (Hexane/EtOAc: 1:4). ^1H NMR (400 MHz, $CDCl_3$): δ 8.02 (1H, d, ArH), 7.74 (2H, m, ArH), 7.50 (1H, t, ArH), 6.39 (1H, q, −CH), 2.79 (4H, s, $-CH_2$), 1.79 (3H, d, $-CH_3$). ^1H NMR spectra of compound H has been shown in Figure S12.

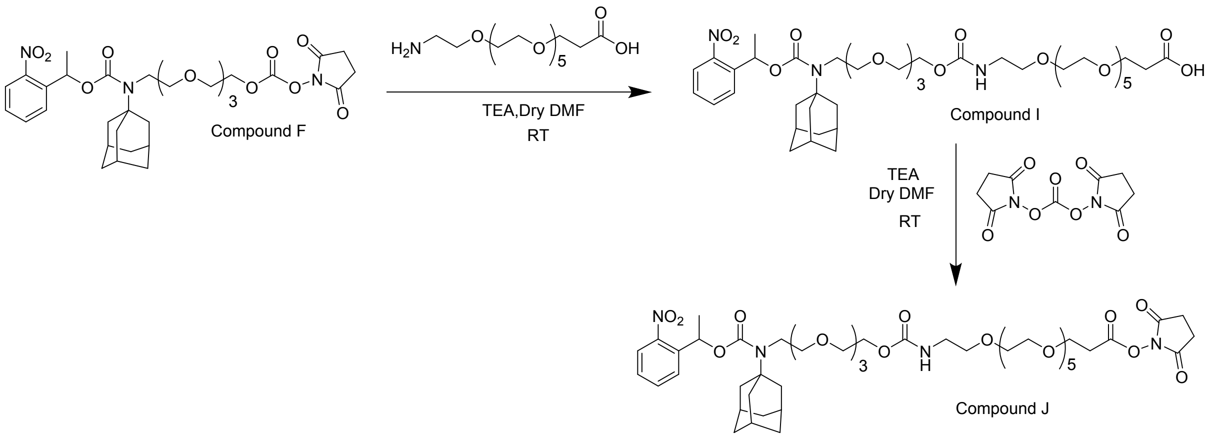

Scheme S3. Synthetic scheme for the preparation of ^{C}ADA–TEG–NHS-B (compound J).

Synthesis of compound I: Amino-PEG6-carboxylic acid (5.0 mg, 0.0076 mmol, 1-Amino-3,6,9,12,15,18-hexaoxahenicosan-21-oic Acid) was dissolved in dry DMF (0.3 mL) and triethylamine (TEA, 3.17 µL, 0.0228 mmol) was added and vortexed for 1 min. To the resulting solution compound F (5.0 mg, 0.0076 mmol) was added and stirred overnight at room temperature. The product was purified by semi-preparative HPLC [Solvents: 0.1% Trifluoroacetic acid (TFA), 5% acetonitrile, water (A), 0.1% Trifluoroacetic acid (TFA), acetonitrile (B); gradient: 5 to 100% of B in 40 min]. The target product was eluted at Rt = 25.6 min to yield compound I as a viscous liquid (6.80 mg, quantitative). LCMS**:** (ESI-MS): calculated m/z 900.47 $[M+H]^+$; found 900.75 $[M+H]^+$.^1H NMR (400 MHz, DMSO-d_6): δ 12.16 (1H, s, br, −COOH), 7.96 (1H, d, ArH), 7.78 (1H , t, ArH), 7.68 (1H, d, ArH), 7.55 (1H, t, ArH), 7.17 (1H, t, −NH−), 5.96 (1H, q, −CH), 4.03 (2H, t, −CH2), 3.60-3.37 (42H, m,

−OCH2CH2O−), 3.12 (2H, m, −CH2), 2.01 (9H, s, −CH, −CH2), 1.58 (9H, m, −CH3, −CH2). ^{1}H NMR spectra of compound I has been shown in Figure S13.

Synthesis of compound J (*ᶜ*ADA–TEG–NHS-B): Compound I (6.80 mg, 0.0076 mmol) was dissolved in dry DMF, triethylamine (TEA, 3.17 µL, 0.0228 mmol) and disuccinimidyl carbonate (DSC, 9.68 mg, 0.0338 mmol) was added and stirred overnight at room temperature. The product was purified by semi-preparative HPLC [Solvents: 0.1% Trifluoroacetic acid (TFA), 5% acetonitrile, water (A), 0.1% Trifluoroacetic acid (TFA), acetonitrile (B); gradient: 5 to 100% of B in 40 min]. The target product was eluted at R_t = 28.0 min to yield compound J as a viscous liquid (7.50 mg, quantitative). HRMS**:** (ESI-MS): calculated m/z 997.4864 $[M+ H]^+$, 1014.5129 $[M +NH_4]^+$; found 997.4849 $[M+ H]^+$, 1014.5115 $[M +NH_4]^+$. ^{1}H NMR (600 MHz, DMSO-d_6): δ 7.96 (1H, d, ArH), 7.78 (1H , t, ArH), 7.68 (1H, d, ArH), 7.54 (1H, t, ArH), 7.18 (1H, t, −NH−), 5.94 (1H, q, −CH), 4.02 (2H, t, −CH2), 3.70-3.40 (36H, m, −OCH2CH2O−), 3.10 (2H, t, −CH2), 2.91 (2H, t, −CH2), 2.91 (4H, s, −CH2), 2.00 (9H, s, CH, −CH2), 1.56 (9H, m, −CH3, −CH2). ^{1}H NMR spectra of compound J has been shown in Figure S14.

2.2. Synthesis of *ᶜ*ADA-Fluorophores:

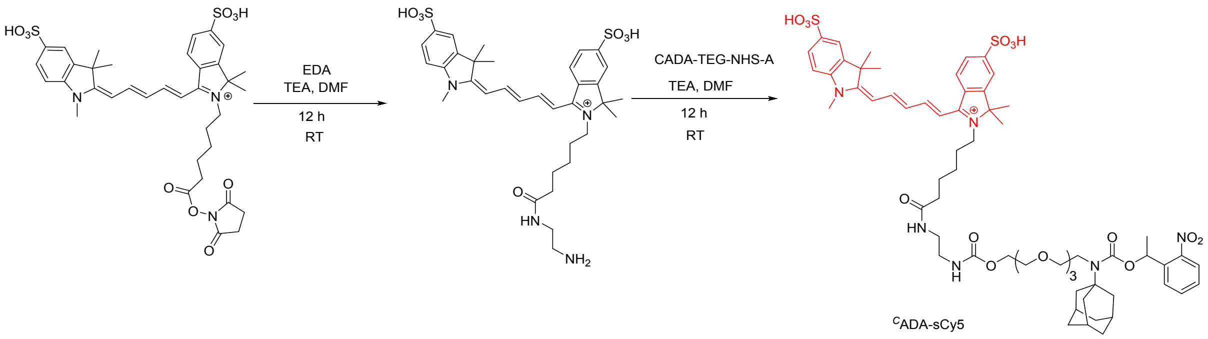

Scheme S4. Synthetic scheme for the preparation of Sulpho-cy5 conjugated *ᶜ*ADA (*ᶜ*ADA─sCy5).

Synthesis of Sulpho-cy5-diamine: A mixture of Sulfo-Cyanine5 NHS ester (0.200 mg, 0.262 µmole), ethylene diamine (0.048 mg, 0.788 µmole) and triethylamine (TEA, 0.100 µL, 0.788 µmole) in DMF (25 µL) was stirred overnight at room temperature. The product was purified by semi-preparative HPLC [Solvents: 0.1% Trifluoroacetic acid (TFA), 5% acetonitrile, water (A), 0.1% Trifluoroacetic acid (TFA), acetonitrile (B); gradient: 5 to 50% of B in 20 min and 50% to 100% of B in 40 min]. The target product was eluted at R_t = 9.0 min (yield: quantitative). HRMS**:** (ESI-MS): calculated m/z 685.2725 $[M]^+$, found 685.2665 $[M]^+$.

Synthesis of ^{C}ADA─sCy5: A mixture of Sulfo-Cyanine5 diamine (11.0 µg, 0.016 µmole), ^{C}ADA–TEG–NHS-A (Compound F, 31.0 µg, 0.048 µmole) and triethylamine (TEA, 6.74 nL, 0.048 µmole) in DMF (10 µL) was vortexed overnight at room temperature. The product was purified by HPLC [Solvents: 0.1% Trifluoroacetic acid (TFA), 5% acetonitrile, water (A), 0.1% Trifluoroacetic acid (TFA), acetonitrile (B); gradient: 5 to 50% of B in 20 min and 50% to 100% of B in 40 min]. The target product was eluted at R_t = 20.0 min (yield: quantitative). HRMS**:** (ESI-MS): calculated m/z 1231.5301 $[M]^+$, found 1231.5131 $[M]^+$.

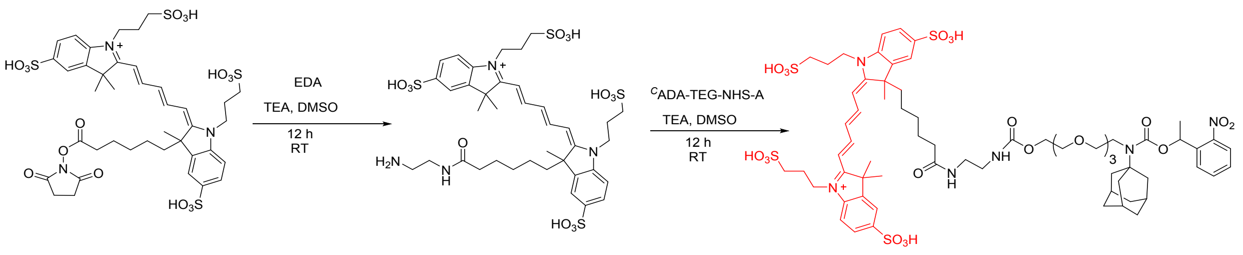

^{C}ADA-Alexa647

Scheme S5. Synthetic scheme for the preparation of Alexa Fluor 647 conjugated ^{C}ADA (^{C}ADA─Alexa647).

Synthesis of Alexa647-diamine: A mixture of Alexa Fluor 647 NHS ester (0.100 mg, 0.103 µmole), ethylene diamine (0.018 mg, 0.309 µmole) and triethylamine (TEA, 0.043 µL, 0.309 µmole) in DMSO (25 µL) was stirred overnight at room temperature. The product was purified by semi-preparative HPLC [Solvents: 0.1% Trifluoroacetic acid (TFA), 5% acetonitrile, water (A), 0.1% Trifluoroacetic acid (TFA), acetonitrile (B); gradient: 5 to 100% of B in 30 min]. The target product was eluted at R_t = 11.3 min (yield: quantitative). LCMS**:** (ESI-MS): calculated m/z 901.25 $[M]^+$, found 901.38 $[M]^+$.

Synthesis of ^{C}ADA─Alexa647: A mixture of Alexa647-diamine (5.0 µg, µmole), ^{C}ADA–TEG–NHS-A (Compound F, 11.0 µg, 0.017 µmole) and triethylamine (TEA, 2.32 nL, 0.017 µmole) in DMSO (10 µL) was vortexed overnight at room temperature. The product was purified by HPLC [Solvents: 0.1% Trifluoroacetic acid (TFA), 5% acetonitrile, water (A), 0.1% Trifluoroacetic acid (TFA), acetonitrile (B); gradient: 5 to 50% of B in 20 min and 50% to 100% of B in 40 min]. The target product was eluted at R_t = 21.6 min (yield: quantitative). HRMS **:** (ESI-MS): calculated m/z 722.2423 $[M-3H]^{2-}$, found 722.2406 $[M-3H]^{2-}$.

2.3. Synthesis of ^{C}ADA-Targeting Ligands:

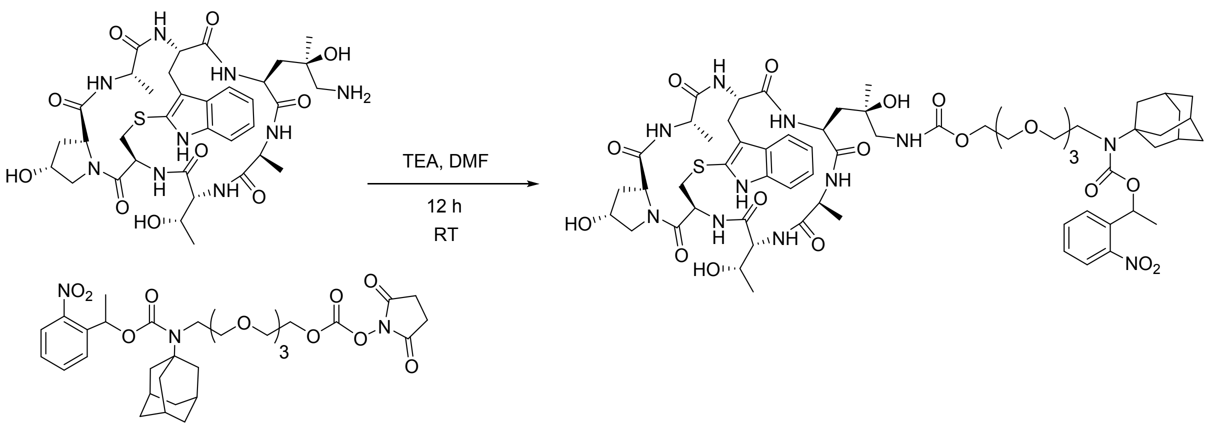

Scheme S6. Synthetic scheme for the preparation of ^{C}ADA─phalloidin.

Synthesis of ^{C}ADA─phalloidin: A mixture of Amino-Phalloidin ([Leu(4-OH,5-NH_2)$_7$], 25.0 µg, 0.032 µmole), ^{C}ADA–TEG–NHS-A (Compound F, 104.9 µg, 0.159 µmole) and triethylamine (TEA, 22.0 nL, 0.159 µmole) in DMF (25 µL) was stirred for 3 h at room temperature. The product was purified by semi-preparative HPLC [Solvents: 0.1% Trifluoroacetic acid (TFA), 5% acetonitrile, water (A), 0.1% Trifluoroacetic acid (TFA), acetonitrile (B); gradient: 5 to 35% of B in 17 min and 35% to 100% of B in 25 min]. The target product was eluted at R_t = 22.5 min (yield: 47.5 %). HRMS**:** (ESI-MS): calculated m/z 1334.5973 $[M+H]^+$, found 1334.5726 $[M+H]^+$.

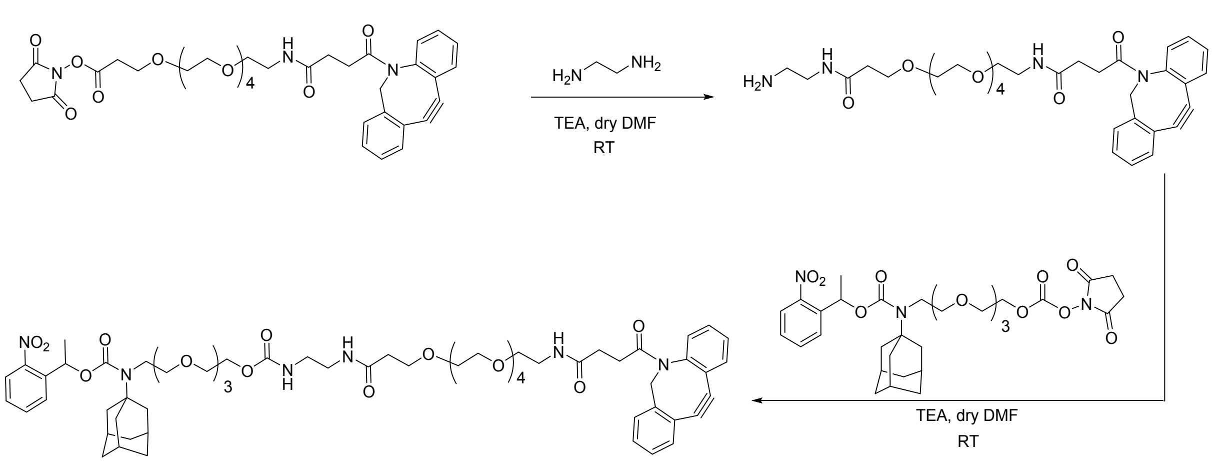

Scheme S7. Synthetic scheme for the preparation of ^{C}ADA─DBCO.

Synthesis of DBCO-diamine: A mixture of DBCO-PEG-NHS ester (0.200 mg, 0.290 µmole), ethylene diamine (EDA, 0.058 µL, 0.870 µmole) and triethylamine (TEA, 0.121 µL, 0.870 µmole) in DMF (25 µL) was stirred overnight at room temperature. The product

was purified by semi-preparative HPLC [Solvents: 0.1% Trifluoroacetic acid (TFA), 5% acetonitrile, water (A), 0.1% Trifluoroacetic acid (TFA), acetonitrile (B); gradient: 5 % to 100% of B in 35 min]. The target product was eluted at R_t = 11.0 min (yield: quantitative). LCMS: (ESI-MS): calculated m/z 639.34 $[M+H]^+$, found 639.43 $[M+H]^+$. ^1H NMR (600 MHz, DMSO-d_6): δ 8.00 (1H, t, −NH−), 7.68-7.65 (3H, m, −NH−), 7.61 (1H, d, ArH), 7.58 (1H, m, ArH), 7.49 (1H, m, ArH), 7.45 (2H, m, ArH), 7.39 (1H, t, ArH), 7.35 (1H, t, ArH), 7.30 (1H, d, ArH), 5.04 (2H, d, $-CH_2$), 3.59-3.43 (22H, m, $-CH_2$-CH_2-), 3.09-2.92 (4H, m, $-CH_2$-CH_2-), 2.83 (2H, t, $-CH_2$), 2.33 (2H, t, $-CH_2$), 2.16 (2H, t, $-CH_2$). ^1H NMR spectra of DBCO-diamine has been shown in Figure S18.

Synthesis of ^{C}ADA─DBCO: A mixture of DBCO-EDA (200.0 µg, 0.313 µmole), ^{C}ADA-NHS (Compound F, 414.1 µg, 0.626 µmole) and triethylamine (TEA, 130.9 nL, 0.939 µmole) in DMF (25 µL) was vortexed overnight at room temperature. The product was purified by semi-preparative HPLC [Solvents: 0.1% Trifluoroacetic acid (TFA), 5% acetonitrile, water (A), 0.1% Trifluoroacetic acid (TFA), acetonitrile (B); gradient: 5 % to 100% of B in 35 min]. The target product was eluted at R_t = 22.5 min (yield: 44.5%). HRMS: (ESI-MS): calculated m/z 1185.5966 $[M+H]^+$, found 1185.5956 $[M+H]^+$. ^1H NMR (600 MHz, DMSO-d_6): δ 7.95 (1H, d, ArH), 7.86 (1H , t, ArH), 7.76 (1H, t, ArH), 7.67 (2H, m, −NH−), 7.62 (1H, d, ArH), 7.58 (1H, m, ArH), 7.54 (1H, t, ArH), 7.50 (1H, m, ArH), 7.45 (2H, m, ArH), 7.38 (1H, t, ArH), 7.34 (1H, t, ArH), 7.30 (1H, d, ArH), 7.17 (1H, t, −NH−), 5.95 (1H, q, −CH), 5.03 (2H, d, $-CH_2$), 4.02 (2H, m, $-CH_2$), 3.63-3.41 (40H, m, $-CH_2$-CH_2-), 3.06-2.99 (4H, m, $-CH_2$-CH_2-), 2.27 (2H, t, $-CH_2$), 2.15 (2H, t, $-CH_2$), 1.99 (9H, s, CH, $-CH_2$), 1.56 (9H, m, $-CH_3$, $-CH_2$). ^1H NMR spectra of ^{C}ADA-DBCO has been shown in Figure S19.

2.4. Synthesis of Tetraacylated mannosamine azide (Ac_4ManNAz):

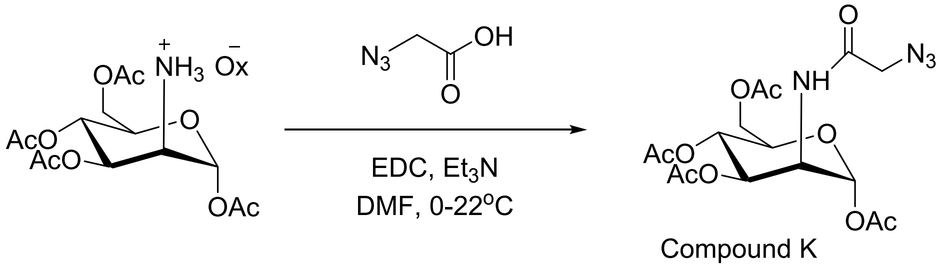

Compound K

Scheme S8. Synthetic scheme for the preparation of tetraacylated mannosamine-azide. The starting materil (Man(OAc)$_4$NH$_3^+$Ox$^-$) was synthesised using previously reported protocol. [1]

Synthesis of Tetraacylated mannosamine-azide (compound K): Man(OAc)$_4$NH$_3^+$Ox$^-$ (20.00 mg, 0.046 mmol) was dissolved in dry DMF (1 mL) in a 10 mL RB flask. A solution of 2-azidoacetic acid (9.76 mg, 0.966 mmoles) in DMF (0.5 mL) was prepared and added to the RB flask with continuous stirring. After 5 min of stirring, the RB flask was cooled to 0°C and triethylamine amine was added. The reaction mixture was allowed to warm to room temperature, which was then stirred for 12 h. Upon completion of the reaction, (monitored by TLC), the reaction mixture was immediately evaporated to dryness using rota-vapour, diluted with 20 mL water and extracted with dichloromethane (DCM) (3 × 40 mL). The crude product was purified by silica column (230-400 mess) chromatography (eluent: Hexane/EtOAc) to yield Man(OAc)$_4$NHCOCH$_2$N$_3$ as a white viscous solid (5.0 mg, yield: 25.4 %).^{1}H NMR (400 MHz, DMSO-d$_6$): δ 8.53 (1H, d, −NH), 5.83 (1H , d, −CH), 5.23 (1H,dd, −CH), 5.14 (1H,dd, −CH), 4.44 (1H, m, −CH), 4.18 (1H, dd, −CH), 4.02 (2H, m, −CH$_2$), 3.94 (2H, s, −CH$_2$), 2.16-1.94 (12H, s, −CH$_3$). ^{1}H NMR spectra of compound K has been shown in Figure S15.

2.5. Synthesis of CB[7]-Fluorophores:CB[7]– monoamine

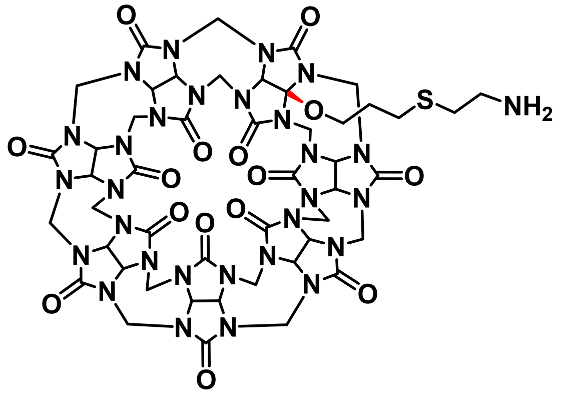

Note: CB[7] monoamine has been synthesized using our previously reported protocol.[2]

CB[7]–Alexa 647 conjugate:

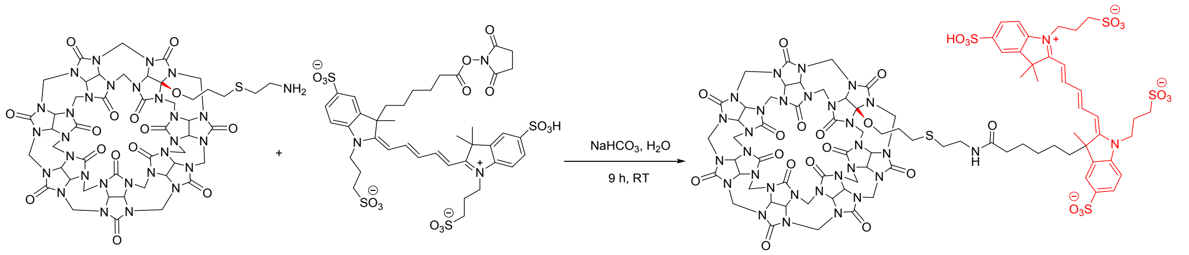

CB[7] monoamine (100 µg, 0.0771 µmol) was dissolved in milli-Q water (70 µL) in a microcentrifuge tube and 1M $NaHCO_3$ (10 µL) was added to it. The reaction mixture was stirred at room temperature for 15 min. Alexa 647 NHS ester (161 µg, 16.1 µL from stock of 10 mg.mL^{-1} in DMSO, 0.1543 µmol) was then added to it and stirred at room temperature for 9 h. The crude product was purified by using reversed phase high performance liquid chromatography [Solvents: 0.1% Trifluoroacetic acid (TFA) in water (A), 0.1% Trifluoroacetic acid (TFA) in acetonitrile (B); gradient: 5 % to 25% of B in 40 min] to yield 12% Alexa 647 conjugated CB[7]. The target product was eluted at R_t = 25.0 min. HRMS**:** (ESI-MS): calculated m/z 1142.8010 [M + cystamine]$^{2-}$, found 1142.7266 [M + cystamine]$^{2-}$.

CB[7]–Fluorescein conjugate:

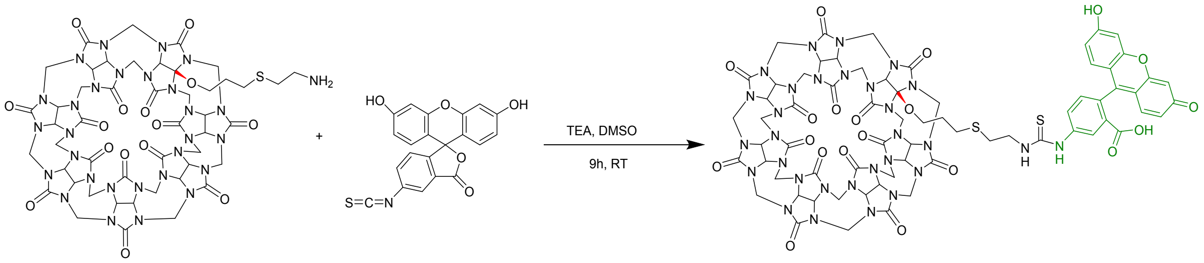

CB[7] monoamine (166.67 µg, 33.4 µL from 5 mg/mL stock in dry DMSO, 0.1286 µmol) was taken in microcentrifuge tube. Triethylamine (0.5 µL from 10 % (v/v) stock in dry DMSO, 0.3858 µmol) was added and stirred at room temperature for 15 min. Then, fluorescein isothiocyanate (125.18 µg, 5.0 µL from 25 mg/mL stock in dry DMSO, 0.3215 µmol) was added into the reaction mixture and stirred at room temperature for 9 h. The reaction mixture was diluted using DMSO (make upto 60 µL) and purified by HPLC [Solvents: 0.1% Trifluoroacetic acid (TFA) in water (A), 0.1% Trifluoroacetic acid (TFA), acetonitrile (B); gradient: 5 % to 80% of B in 40 min and 80% to 100% in 41 min]. The target product was eluted at R_t = 16.8 min (yield = 47%). The purified product was dried under

vacuum and characterized by HRMS. HRMS: (ESI-MS): calculated 919.2471 $[M+Cys+2H]^{2+}$; Found 919.2497 $[M+Cys+2H]^{2+}$.

CB[7]– BHQ3 conjugate:

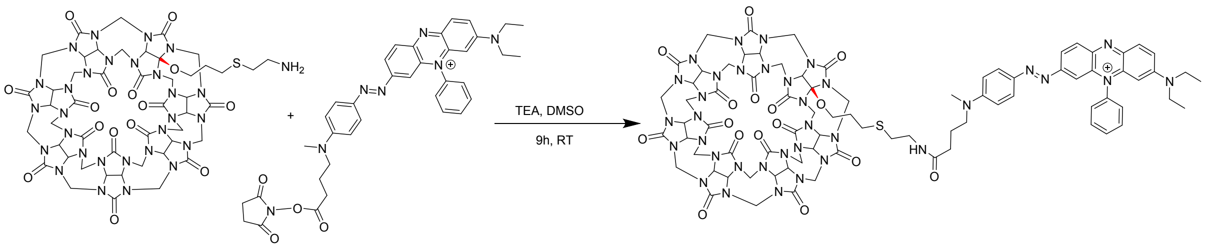

CB[7] monoamine (985 μg, 0.7603 μmol) was taken in microcentrifuge tube. Triethylamine (0.212 μL, 1.5206 μmol) was added and stirred at room temperature for 5 min. Then, NHS ester of BHQ3 (200.0 μg, 20.0 μL from 10 mg/mL stock in dry DMF,0.253 μmol) was added into the reaction mixture and stirred at room temperature for 9 h. The reaction mixture was diluted using DMSO (make up to 200 μL) and purified by HPLC [Solvents: 0.1% Trifluoroacetic acid (TFA) in water (A), 0.1% Trifluoroacetic acid (TFA) in acetonitrile (B); gradient: 5% to 40% of B in 10 min to 100% in 30 min]. The target product was eluted at R_t = 12.0 min (yield = 41 %). The purified product was dried under vacuum and characterized by HRMS. HRMS: (ESI-MS): calculated m/z 659.5765 $[M + cystamine + 2H]^{2+}$; found 659.5756 $[M + cystamine + 3H]^{3+}$.

Synthesis of CB[7]–Cy3 conjugate

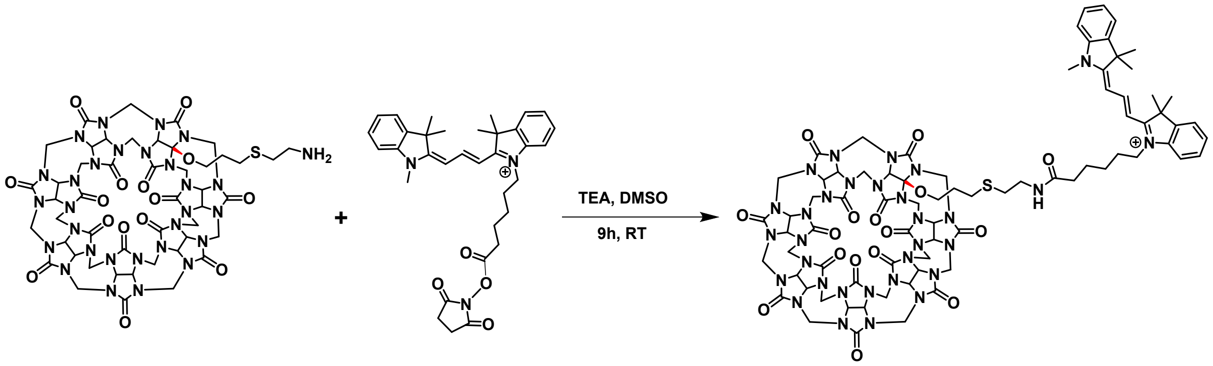

CB[7]–NH_2 (330 μg, 66 μl from 5 $mg.mL^{-1}$ stock in dry DMSO, 0.2546 μmol) was taken in microcentrifuge tube. Triethyl amine (1.1 μL from 10% (v/v) stock in dry DMSO, 0.7638 μmol) was added into it and stirred at room temperature for 15 min. Then, NHS ester of Cy3 (407 μg, 40.7 μl from 10 $mg.mL^{-1}$ stock in dry DMSO, 0.6345 μmol) was added into the reaction mixture and stirred at room temperature for 9 h. The reaction mixture was diluted

using water and injected to HPLC for purification. The purified product (yield: 45%) was dried under vacuum and characterized by ^{1}H NMR and HRMS. ^{1}H NMR (DMSO–d^6, 600 MHz): δ 8.35-8.31 (1H, t), 7.94-7.93 (1H, t), 7.64-7.63 (2H, m), 7.52-7.42 (3H, m), 7.31-7.26 (2H, m), 6.50-6.45 (2H, m), 5.76-5.63 (12H, m), 5.55-5.51 (7H, m), 5.32-5.43 (12H, m), 4.28-4.14 (15H, m), 3.65-3.63 (2H, m), 3.49-3.48 (2H, m), 3.18- 3.15 (3H, br), 2.62-2.58 (6H, br), 2.37-2.36 (4H, m), 2.17-2.15 (2H, m), 1.85-1.83 (2H, m), 1.54-1.44 (4H, m), 1.36-1.34 (2H, m), 0.96-0.80 (6H, br). HRMS (ESI–MS): calculated 943.8628 $[M+cys+H]^{2+}$, 629.5776 $[M+cys+2H]^{3+}$; Found 943.8635 $[M+cys+H]^{2+}$, 629.5788 $[M+cys+2H]^{3+}$. ^{1}H NMR spectra of CB[7]-Cy3 has been shown in Figure S16.

Synthesis of CB[7]–TAMRA conjugate

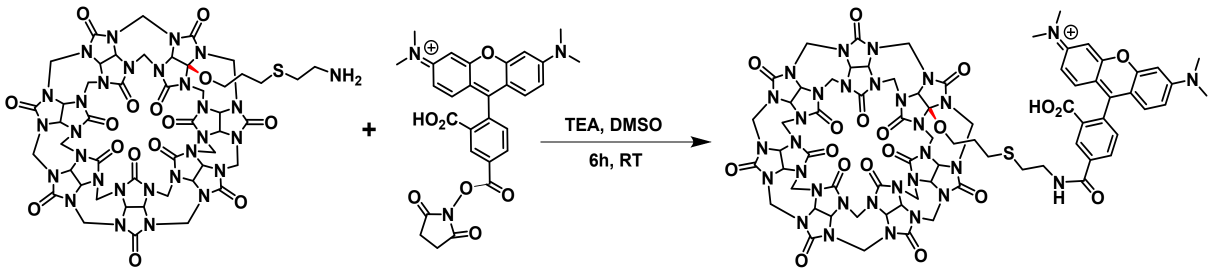

CB[7]–NH_2 (500 µg, 100 µl from 5 mg.mL^{-1} stock in dry DMSO, 0.3857 µmol) was taken in microcentrifuge tube. Triethyl amine (1.61 µl from 10% (v/v) stock in dry DMSO, 1.1571 µmol) was added into it and stirred at room temperature for 15 min. Then, NHS ester of TAMRA (508.67 µg, 50.88 µl from 10 mg.mL^{-1} stock in dry DMSO, 0.9642 µmol) was added into the reaction mixture and stirred at room temperature for 9 h. The reaction mixture was diluted using water and injected to HPLC for purification. The purified product (yield: 51%) was dried under vacuum and characterized by ^{1}H NMR and HRMS. ^{1}H NMR (400MHz, DMSO-d^6): δ 9.02 (1H, m), 8.67 (1H, br), 8.26-8.28 (1H, m), 7.59-7.61 (1H, m), 7.21 (2H, s), 7.08 (2H, s), 6.95 (2H, s), 5.63-5.76 (12H, m), 5.51-6.55 (4H, m), 5.32-3.43 (12H, m), 4.14-4.28 (13H, m), 3.68-3.70 (12H, br), 3.61-3.62 (2H, m), 3.58-3.59 (2H, m), 2.80 (2H, m), 2.78 (2H, br), 1.74-1.77 (2H, m). HRMS (ESI–MS): calculated 620.8693 $[M+cys+3H]^{3+}$; Found 620.8694 $[M+cys+3H]^{3+}$. ^{1}H NMR spectra of CB[7]-TAMRA has been shown in Figure S17.

2.6. Synthesis of CB[7]─phalloidin conjugate:

Actin targeting CB[7]─phalloidin conjugate has been synthesized using our previously reported literature. [2]

2.7. Synthesis and characterization of ^{C}ADA conjugated antibody:

1. Secondary antibodies (donkey anti-rat) was buffer exchanged by Zeba spin column pre–equilibrated with PBS containing 10% 1(M) $NaHCO_3$.

2. The concentration of the antibody was measured and used for the conjugation with ^{C}ADA–TEG–NHS-B (Compound J).

3. Buffer exchanged secondary antibody (100 µg, 6.67 x10^{-4} µmol) was first taken in 1.5 mL micro centrifuge tube.

4. 1.33 µL solution (Conc. of 10 mg.mL^{-1} in DMF) of ^{C}ADA–TEG–NHS-B (Compound J) was diluted to 5 µL by DMF (3.67 µL) and added to the antibody solution in two portions (2.5 µL each time).

5. The reaction was kept at 4°C for overnight.

6. ^{C}ADA conjugated antibody was then purified by Zeba spin column (pre–equilibrated with PBS) and characterized by MALDI mass spectrometry. MALDI mass spectrum has been shown in Figure S25.

MALDI−MS analysis for characterization of ^{C}ADA conjugated antibody:

Antibodies (1 µL) after desalting through Zeba spin column (~ 1 mg.mL^{-1} in miliQ water) was taken in micro centrifuge tube containing 1 µL of sinapinic acid (10 mg.mL^{-1}in 50:50 water (0.1% TFA)/acetonitrile).The mixed solution was placed onto a MALDI plate and allowed to dry at room temperature for analysis.

3. Photochemical reaction of ^{C}ADA─TEG─OH (Compound E): Photochemical reaction of ^{C}ADA─TEG─OH at a concentration of 100 µM was performed in water-acetonitrile mixture (9:1 v/v). A solution of the ^{C}ADA─TEG─OH under investigation was irradiated using a UV LED light source (λ = 365 nm). Specifically, 100 µM buffered solution of the sample was kept in a 1 cm quartz cuvette and irradiated with 50 mW/cm^2 emitted light from a handheld LED source. UV-Vis absorption spectra at various time intervals were recorded using a UV-Vis spectrophotometer for a total of 100 s (each measurement was taken at 10 s

interval). UV-Vis spectral changes due to cleavage of photolabile benzyl-N / $PhCH_2$-N bond and the generation of nitrosobenzaldehyde group were recorded.

4. Computational methods:

Simulation Model: A 126 atom cucurbituril (CB[7]) and adamantane based molecules ADA (35 atoms) and ^{C}ADA (56 atoms) are considered. For individual simulations with either adamantane based molecules, CB[7] is kept at different center of mass (COM) distances between 0.0 to 1.0 nm, between CB[7] and ADA/^{C}ADA. Individual CB[7] + ADA/^{C}ADA systems are kept in a box sized 5.0×5.0×5.0 nm^3, periodic in all 3 directions. The box is solvated with TIP3P water molecules.[3] Bonding and non-bonding parameters for CB[7], ADA and ^{C}ADA are generated with CHARMM-GUI, using Antechamber to generate the parameter and topology files.[4, 5]

All simulations are performed using the open source computational program GROMACS.[6] Individual snapshots of solvated CB[7] + ADA/^{C}ADA, at different COM distances, are simulated using the umbrella sampling approach, implemented in GROMACS. An umbrella potential $U = K_x(x - x_0)^2$ is applied, where x_0 is the COM distance between CB[7] and adamantane. Between 0.0 and 1.0 nm, 14 windows are considered with varying K_x. NPT simulations are performed which maintain a temperature of 300 K, using the V-rescale algorithm.[7] A constant pressure of 1 bar is maintained using the Parinello-Rahman barostat.[8] For each window, 10 ns simulation is performed, at a timestep of 2 fs. From the biased umbrella sampling simulations, the unbiased free energy is obtained using the Weighted Histogram Analysis Method (WHAM).[9]

5. FRET quenching titration of ^{C}ADA─Alexa 647 with increasing amount of CB[7]─BHQ3

Titration without irradiation

The FRET quenching based assay for determination of ^{C}ADA─Alexa 647 binding with CB[7]─BHQ-3 was performed in a fluorescence microplate reader at 25°C. ^{C}ADA─Alexa 647 fluorophore was taken in a black 96 well microplate (Costar, non-binding surface). A concentration of 100 nM in 200 µL PBS (pH=7.4) for ^{C}ADA─Alexa 647 was used in this experiment. CB[7]─BHQ3 was added to this solution in a stepwise manner. 1 µL of 5 µM CB[7]─BHQ3 (final concentration 25 nM, 0.25 eq) was added in each step, followed by

recording of fluorescence emission between 650─750 nm (λ_{ex} = 620 nm) to determine the FRET response. The titration was continued for a total of 2 eq of CB[7]─BHQ3.

Titration after irradiation

First, a ^{C}ADA─Alexa 647 solution (100 nM in 200 µL PBS (pH=7.4)) was irradiated for 5 min with 50 mW/cm^2 emitted light from a handheld 365 nm LED source. A similar experimental protocol (as described above) was used for the titration of the photoactivated ^{C}ADA─Alexa 647 with CB[7]─BHQ3.

6. Photoactivated host-guest binding analysis by microscale thermophoresis

The strength of the binding interaction of CB[7] and ^{C}ADA was measured using the technique of microscale thermophoresis (MST). This technique enables the determination of the dissociation constant based on the directed motion of substrate molecules over a thermal gradient. This has been measured by the change in fluorescence intensity with respect to the change in the concentration of the ligand molecule. Sulpho-Cy5 fluorophore conjugated ^{C}ADA molecule (^{C}ADA─sCy5) was used as a guest for the CB[7] host ligand. The measurements were made before and after 365 nm irradiation (t = 5 min, 50 mW/cm^2 emitted light from a handheld LED source) and fluorophore concentration was maintained at 20 nM while CB[7] concentration was varied (3─400 nM) . As seen in Figure S23, the MST curve obtained prior to irradiation shows no constant increase in fluorescence intensity with increasing ligand concentration indicating the absence of binding interactions between the molecules. However, upon irradiation the sigmoidal MST curve clearly indicates the host-guest interaction between CB[7] and ^{C}ADA─sCy5 upon successful photocleavage.

7. Structural characterization of host-guest complexation by ^{1}H NMR

^{1}H NMR was used to monitor the complexation of ^{C}ADA─TEG─OH (5 mM) with CB[7] before and after photoirradiation using 365 nm light (100 mW/cm^2 emitted light from a handheld LED source) for a period of 5 min. Incubation of ^{C}ADA─TEG─OH with CB[7] did not show any significant change in proton signatures of ADA. However, the NMR spectrum post irradiation showed a shift of the C–H protons of ADA group towards upfield direction, denoting the binding of adamantyl moiety inside CB[7] (Figure S24). The photocleavage of ^{C}ADA─TEG─OH was also evident from the transition in colour of the solution from transparent to faint yellow, indicating the formation of yellow nitrosobenzaldehyde molecule.

^1H NMR spectrums were taken in a 1:1 mixture of D_2O/DMSO-d_6 throughout all experiments due to the poor solubility of ^{C}ADA─TEG─OH in D_2O.

8. General cell culture protocol

1. HeLa, MEF, U2OS, A549, MCF7 and HEK cells were used for the experimental study.
2. HeLa, MEF, A549, MCF7 and HEK cells were cultured in a humidified atmosphere (5% CO_2) at 37°C and grown in Dulbecco's Modified Eagle's Medium (DMEM, high glucose) supplemented with 10% fetal bovine serum (FBS) (Gibco, USA), 2 mM Glutamax (Invitrogen, USA) and 1% antibiotics (100 U/mL penicillin, 100 µg/mL streptomycin and 0.25 µg/mL amphotericin) (Gibco, USA). U2OS was cultured in McCoy's 5A medium supplemented with same percentages of FBS and antibiotic.
3. At ~80% confluence, the cells were washed with DPBS (pH 7.3) (Gibco, USA), trypsinized and suspended in culture medium.
4. Cells were then counted and then in a typical experiment, 200 µL of cell suspension having ~20,000 cells/mL were plated in 8-well chamber slide system (Eppendorf, 1.5 glass bottom). In subset of experiments, 35 mm glass bottom cell imaging dish (1 or 1.5 glass bottom) was also employed for the imaging study.
5. The cells were then maintained again in a humidified atmosphere (37°C, 5% CO_2) for 24 h to reach ~60-80 % confluence.
6. Cells were subsequently used for imaging studies.

9. Systems used for microscopic investigation.

Structured illumination microscopy (SIM) imaging

Immunostained cells in an 8–well chamber were imaged using SIM based super-resolution imaging method. An inverted Zeiss ELYRA PS1 microscope was used for this purpose. Three lasers were used for the experiments: 405 nm (source: 50 mW), 561 nm (source: 200 mW) and 642 nm (source: 150 mW). Imaging was performed using two Zeiss oil–immersion objectives: Plan–apochromat DIC 63×/1.40 Oil DIC M27, numerical aperture (NA) 1.40 oil and Plan–apochromat DIC 40x/1.30 Oil DIC M27, numerical aperture (NA) 1.30 oil. Fluorescence light was spectrally filtered with emission filters (MBS– 405+ EF BP 420–480/LP 750 for laser line 405, MBS– 561+EF BP 570–650/LP 750 for laser line 561 and MBS–642+EF LP 655 for laser line 642) and imaged using a PCO edge sCMOS camera. Structured illumination images were processed using structured illumination analysis package for Zen software (Zeiss). Additional softwares have been used for image processing (ImageJ)

and data analysis (Origin 9.0). All the images before and after irradiation were taken with same laser power and integration time.

Note: In all the SIM imaging, before and after irradiation images were taken with same laser power, exposure time, and other acquisition parameters.

Laser confocal scanning microscopy (LCSM) imaging using Zeiss.

Cells in a 8–well chamber were imaged using Zeiss LSM 880 and Zeiss LSM 510 Meta microscope. Four lasers were used for the experiments: 405 nm (source: 30 mW), 488 nm (source: 30 mW), 561 nm (source: 30 mW) and 633 nm (source: 5 mW) in LSM 880. In LSM 510 Meta for imaging only 633 nm (source: 5 mW) laser was used. Imaging was performed using two Zeiss oil–immersion objectives: Plan–apochromat DIC 63×/1.40 Oil DIC M27, numerical aperture (NA) 1.40 oil and Plan–apochromat DIC 40×/1.30 Oil DIC M27, numerical aperture (NA) 1.30 oil. Fluorescence light was spectrally filtered with emission filters (MBT 488/561/633 + Filters: 491-554/570-623/635-735) and imaged with PMT detector. Confocal images were processed using Zen 2.1 SP3 (Zeiss) and ImageJ software. Two-photon activation was carried out using Tsunami Spectra-Physics Millennia Pro 50 mW pump-diode laser fitted to LSM 510 Meta.

Photoactivation by confocal laser scanning microscope (CLSM): The ROI based photoactivation is achieved using photo-bleaching by 405 nm laser. For bleaching 80% laser power for 90 s was used in all experiments related to CLSM, unless stated otherwise.

Note: In all the CLSM imaging, before and after irradiation images were taken with same laser power, exposure time, and other acquisition parameters.

Laser confocal scanning microscopy (LCSM) imaging using Leica.

Cells in a 35 mm glass bottom dish were imaged using Leica TCS SP8 microscope. Four lasers were used for the experiments: 405 nm (source: 50 mW), 488 nm (source: 20 mW), 552 nm (source: 20 mW) and 638 nm (source: 30 mW) in LSM 880. Imaging was performed using Leica oil–immersion objectives: HC PL APO CS2 63x with numerical aperture (NA) 1.40. Fluorescence light was spectrally filtered with emission filters (TD 488/561/633) and imaged with HyD detector. Confocal images were processed using LAS X (Leica) and ImageJ software.

10. Host-guest interaction mediated temporal actin labelling protocol in fixed cellular environment:

1. Removal of culture media from 8-well chamber slide system containing adherent MEF cells.

2. Fixation for 15 min with 200 μL 4% paraformaldehyde in PBS.
3. Washing with 200 μL PBS (3×).
4. Permeabilization with 200 μL 0.25% v/v Triton X–100 in PBS for 10 min.
5. Washing with 200 μL PBS (3×).
6. Blocking for 2 h with 200 μL 3% bovine serum albumin and 0.1% v/v Triton X–100 in PBS at room temperature.
7. Before staining cells were washed with 200 μL PBS (3×).
8. Cells were stained for 2 h at room temperature with phalloidin-CB[7] (2 μM) diluted in PBS solution.
9. Washing cells with 200 μL PBS (2×).
10. Incubation with 200 μL of ^{C}ADA─sCy5 conjugate (250 nM) in PBS for 5 min.
11. Structured illumination images were recorded at this point to get an image before photoactivation.
12. Activation using 365 nm LED light (50 mW/cm^2 emitted light from the handheld LED source) for 90 s for photoactivation of ^{C}ADA─sCy5 conjugate.
13. Structured illumination images were recorded without washing the excess fluorophore after 5 min of the irradiation period. 1% of the total laser power (642 nm laser) was used for SIM imaging. Imaging parameters are kept constant before and after irradiation imaging to have a direct comparison.

11. Host- guest interaction mediated spatiotemporal microtubule labeling in fixed MEF cells:

1. Removal of culture media from 8-well chamber slide system.
2. Washing cells with 200 μL PBS (2×).
3. Fixation for 7 minutes with 200 μL chilled methanol at −20°C followed by washing for three times with PBS.
4. Blocking for 2 h with 200 μL 3% bovine serum albumin in PBS at room temperature.
5. Incubation for 24 h at 4°C with primary antibody (10 μg.mL^{-1}, 200 μL) diluted in PBS containing 3% bovine serum albumin.
6. Washing with 200 μL PBS (3×) with 5 min incubation each time.
7. Incubation for 2 h with ^{C}ADA conjugated secondary antibody (10 μg.mL^{-1}, 200 μL) in 3% bovine serum albumin and 0.1 % triton x in PBS at room temperature.
8. Washing with 200 μL PBS (3×) with 5 min incubation each time.
9. Incubation with 200 μL of CB[7]─TAMRA (100 nM) in PBS.

10. Sub-cellar region of interest (ROI) was selected in the CLSM set-up.
11. ^{C}ADA decorated microtubule filaments in the sub-cellular ROI was activated using CLSM setup. 405 nm laser light was employed in photobleaching mode for 90 s.
12. After irradiation, cells were allowed to bind with imager for 15 min and CLSM images were recorded after washing the excess fluorophore. [Experimental parameters – Laser: 561 nm with 3.0 % power, Detection wavelength: 579-624 nm, Digltal gain: 1.00, Pinhole: 0.86 AU]

12. Time lapse imaging of photoactivated host-guest based labelled microtubules in fixed U2OS:

1. U2OS cells were grown in 8 well imaging dish.
2. Cells were fixed with methanol and tagged with ^{C}ADA conjugated antibody as described previously.
3. Activation using 365 nm LED light (50 mW/cm^2 emitted light from the handheld LED source) was carried out for 90 s.
4. Incubation with 200 μL of CB[7]-TAMRA (100 nM) in PBS for 5 min.
5. Washing with 200 μL PBS (2×).
6. 400 μL of PBS was added to the CB[7]-ADA mediated microtubule labelled cells before the image acquisition.
7. To avoid the evaporation or leakage of solution, the imaging dish is wrapped and sealed with parafilm.
8. Fluorescence imaging was done using Olympus inverted fluorescence microscope for a total of 28 h. To avoid photobleaching, a gentle and low intensity illumination from LED source was used and detection was done using sensitive sCMOS camera.
9. Images were captured at an interval of 2-4 h with same exposure time (30 ms) and excitation LED intensity (20 %, 525 nm excitation from LED source).
10. Image processing was done using ImageJ (Fiji) software. Intensity plots were generated along 5-7 μm lines for there different regions of the cells. Lines were started from outside the cell and passed through microtubule regions inside the cell. A normalized intensity plot was drawn using OriginPro 9 software after subtracting the background signal for all the three regions (Region-1, Region-2 and Region-3).

Note: For imaging Olympus oil–immersion objective UPL SAPO 60X objective (numerical aperture (NA) 1.42) and faster and sensitive Optimus sCMOS camera were used. CoolLED

pE – 4000 was used as light source for imaging. Micromanager software was used to perform imaging.

13. Host-guest interaction mediated spatiotemporal actin labeling protocol in fixed Hela cells:

1. Removal of culture media from 8-well chamber slide system.
2. Fixation for 15 min with 200 µL 4% paraformaldehyde in PBS.
3. Washing with 200 µL PBS (3×).
4. Permeabilization with 200 µL 0.25% v/v Triton X–100 in PBS for 10 min.
5. Washing with 200 µL PBS (3×).
6. Blocking for 2 h with 200 µL 3% bovine serum albumin and 0.1% v/v Triton X–100 in PBS at room temperature.
7. Before staining, cells were washed with 200 µL PBS (3×).
8. Cells were stained for 2 h at room temperature with ^{C}ADA─phalloidin (2 µM) diluted in PBS.
9. Washing cells with 200 µL PBS (2×).
10. Incubation with 200 µL of CB[7]-fluorophore conjugate (100 nM) in PBS. CB[7]─Alexa 647 was used for single colour labelling.
11. A single cell was illuminated with 405 nm laser for activation of ^{C}ADA─phalloidin tagged actin fibers. 100 % output from 405 nm laser was used from SIM set-up for 90 s to activate the ^{C}ADA.
12. Structured illumination images were recorded after washing the excess fluorophore after 30 min of photoactivation.

14. Host–guest mediated imaging of thoracic muscle tissue

Dissection of thoracic muscle tissue: Adult wild type drosophila flies were collected which were maintained in 12 h light and 12 h dark at 25°C. Once collected, flies were kept in ice for around 15 minutes for anesthetizing.

Thoracic muscle dissection

1. After flies were anesthetized, they were submerged in PBS, placed dorsally on the dissection plate, and were pierced with insect pins on the abdomen region.
2. Using forceps, top layer of thorax was dissected slowly, peeled out gently and bunch of clustered thoracic muscles were taken out. Dissected muscle tissues were transferred in chilled PBS in labelled wells of the glass dish kept on ice and were allowed to settle.

Photoactivated host-guest interaction mediated actin labeling for thoracic muscle tissue:

1. After dissections were carried out, tissues were fixed with 4% paraformaldehyde (PFA) at room temperature for 30 minutes with gentle shaking.
2. Tissues were washed with at least thrice with 5 minutes incubation for each time using PBS containing 0.5% Triton X–100 (0.5% PBT).
3. Samples were then blocked using 10% horse serum in 0.5% PBT for 1 h at room temperature.
4. ^{C}ADA─phalloidin (2 μM) in 0.5% PBT was added to each chamber and incubated overnight at 4°C.
5. Afterwards, samples were washed three times with 0.5% PBT for 5 min incubation each time.
6. A solution of CB[7]─Alexa 647 (100 nM) was added to the phalloidin labelled tissue and proceed for host-guest mediated photoactivation experiment.
7. Structured illumination image from a tissue region were recorded at this point to get an image before photoactivation.
8. The same tissue region was illuminated with 405 nm laser for activation of ^{C}ADA─phalloidin tagged actin structures in muscle. 100 % output from 405 nm laser was used from SIM set-up for 90 s to activate the ^{C}ADA.
9. Structured illumination images were recorded after washing the excess fluorophore, 30 min after photoirradiation of the tissue sample.

15. Protocol for two-photon activation of actin in MEF cell:

11. Actin was labeled with ^{C}ADA─phalloidin conjugate (2 μM) in PBS buffer for 2 h at room temperature (or, 12 h at 4°C).
12. Excess probe was removed by washing with PBS three times.
13. CB[7]–Alexa 647 fluorophore (100 nM) was incubated with the cells for 30 min before photoirradiation with a pulsed 725 nm laser light from Ti-sapphire laser.
14. After irradiation for 300 s, cells were allowed to bind with the fluorescent CB[7] conjugate for 30 min.
15. Fluorescence microscopy images were recorded after washing the excess fluorophore and exciting the sample with 633 nm laser.

Note: Zeiss LSM 510 Meta was used for two-photon based uncaging and imaging. Imaging was done using 633 nm HeNe laser while two-photon activation was carried out using Ti-sapphire laser (Tsunami spectra-physics, 50 mW). Imaging parameters – Laser: 633 nm, emission wavelength: 680 nm, Digital gain: 1.00, Pinhole: 5.22 AU, Plan–apochromat DIC 63×/1.40 Oil DIC]

16. Host-guest interaction mediated barcoding in MEF cells:

1. Removal of culture media.
2. Washing cells with 200 µL PBS (2×).
3. Fixation for 7 minutes with 200 µL chilled methanol at −20°C followed by washing for three times with PBS.
4. Blocking for 2 h with 200 µL 3% bovine serum albumin in PBS at room temperature.
5. Incubation for 24 h at 4°C with primary antibody (10 µg.mL^{-1}, 200 µL) diluted in PBS containing 3% bovine serum albumin.
6. Washing with 200 µL PBS (3×) with 5 min incubation each time.
7. Incubation for 12 h at 4°C with ^{C}ADA conjugated secondary antibody (10 µg.mL^{-1}, 200 µL) in 3% bovine serum albumin and 0.1 % triton x in PBS at room temperature.
8. Washing with 200 µL PBS (3×) with 5 min incubation each time to remove the excess ^{C}ADA conjugated secondary antibody.
9. Selection of ROI-1 using the CLSM toolbox.
10. Activation of ROI using 405 nm laser for 90 s under photobleaching mode.
11. Incubation of 200 µL of CB[7]─Fluorophore-1 (CB[7]─Fluorescein, 100 nM) in PBS for 5 min.
12. Washing cells with 200 µL PBS (2×).
13. CLSM images were recorded in three channels (488 nm-blue, 561 nm-green and 633 nm-Red).
14. Next cycle follows the repetition of steps 9-12, where ROI-2 is photoactivated followed by incubation of 200 µL of CB[7]─Fluorophore-2 (100 nM CB[7]─TAMRA in PBS) and washing.
15. CLSM images were recorded in three channels.
16. The third cycle follows the selection and photoactivation of ROI-3 (repetition of steps 9-12). Incubation with 200 µL of CB[7]- Fluorophore─3 (100 nM CB[7]─Alexa 647 in PBS)

and respective imaging in three channels. In the above mentioned method, each barcodes were created by using a single fluorophore.

[Experimental parameters – Laser : 633 nm with 2.5 % power / 561 nm with 1.0 % power/ 488 nm with 2.5 % power , Detection wavelength: 635 -735 nm / 571-624 nm / 500-554 nm, Digital gain : 1.00 is same in 3 channels, Pinhole: 0.61 AU].

The next set of barcodes were created by incubating different combinations of fluorophores.

In case of color-coding cells with combination of two fluorophores, steps all the steps are followed from the above protocol but incubation was changed to simultaneous incubation of two or three fluorophores in 1:1 ratio. The following combinations were used:

a) 200 µL of CB[7]─Fluorescein + CB[7]─TAMRA (50 nM each) in PBS and respective CLSM imaging in three channels.
b) 200 µL of CB[7]─Fluorescein + CB[7]─Alexa 647 (50 nM each) in PBS and respective CLSM imaging in three channels.
c) 200 µL of CB[7]─TAMRA + CB[7]─Alexa 647 (50 nM each) in PBS and respective CLSM imaging in three channels.
d) 200 µL of CB[7]- TAMRA + CB[7]-Alexa 647 + CB[7]-Fluorescein (50 nM each) in PBS and respective CLSM imaging in three channels. Here, barcode was generated by using combinations of three fluorophore in 1:1:1 ratio.

[Experimental parameters for **a)** to **c)** – Laser: 633 nm with 5.0 % power / 561 nm with 5.0 % power/ 488 nm with 3.0 % power , Detection wavelength: 635 -735 nm / 571-624 nm / 500-554 nm, Digltal gain : 1.00 is same in 3 channels, Pinhole: 0.85 AU]

[Experimental parameters for **d)** – Laser : 633 nm with 3.0 % power / 561 nm with 3.0 % power/ 488 nm with 3.0 % power , Detection wavelength: 635 -720 nm / 562-625 nm / 500-554 nm, Digital gain : 1.00 is same in 3 channels, Pinhole: 1.00 AU].

Creating barcodes using variable ratio of two-fluorophores.

a) Incubation with 200 µL of CB[7]─Fluorescein (20 nM) + CB[7]─TAMRA (80 nM) in PBS and respective CLSM imaging in three channels.
b) Incubation with 200 µL of CB[7]─Fluorescein (50 nM) + CB[7]─TAMRA (50 nM) in PBS and respective CLSM imaging in three channels.
c) Incubation with 200 µL of CB[7]─Fluorescein (80 nM) + CB[7]─TAMRA (20 nM) in PBS and respective CLSM imaging in three channels. Here, barcodes were created by using combinations of two fluorophore in 8:2, 1:1, 2:8 ratios, respectively.

[Experimental parameters – Laser: 633 nm with 3.0 % power / 561 nm with 3.0 % power/ 488 nm with 3.0 % power , Detection wavelength: 635 -720 nm / 562-625 nm / 500-554 nm, Digital gain : 1.00 is same in 3 channels, Pinhole: 0.85 AU].

We encoded the assembly instruction in the shape of j, n, and c using a similar protocol that is mentioned above for three single colour barcoding schemes. Desired shape ROIs were drawn in sequential steps using the CLSM toolbox.

17. Time lapse imaging of two-color barcodes in MEF cell.

1. MEF cells were grown in 35 mm glass bottom dish.
2. MEF cells were PFA fixed and tagged with ^{C}ADA conjugated antibody as described previously.
3. Imaging was performed using Leica SP8 confocal system.
4. Selection of ROI-1 having square area of 40 x 40 µm using the CLSM toolbox.
5. FRAP module of the confocal system was used to carry out the photoactivation. The selected ROI-1 was irradiated using 405 nm laser (50% intensity, source power: 50 mW) for a period of 60 s. Zoom in mode of bleaching was used with background set to zero for the photoactivation.
6. Incubation of 200 µL of CB[7]─ Fluorophore-1 (CB[7]─Alexa647, 100 nM) in PBS for a time interval of 5 min.
7. Washing with 200 µL PBS (3×).
8. Activation of ROI-2 (square area, 40 x 40 µm) and ROI-3 (square area, 60 x 60 µm) together using 405 nm laser with same laser power and time interval (similar to ROI-1).
9. Incubation of 200 µL of CB[7]─ Fluorophore-2 (CB[7]─TAMRA, 100 nM) in PBS for a time interval of 5 min.
10. Washing with 200 µL PBS (3×).
11. Next, about 400 µL of PBS was added to the cells after washing.
12. To avoid the evaporation or leakage of solution, the imaging dish was carefully sealed with parafilm.
13. CLSM images were captured at an interval of 6 h with same laser intensity and HyD detector for 30 h.
14. Image processing was done using ImageJ (Fiji) software.

Note: The emission in green channel is collected at wavelength from 565-625 nm, upon illumination at an excitation wavelength of 552 nm (0.75 % laser intensity) and emission in

red channel is collected at wavelength from 650 to 710 nm (1% laser intensity), upon illumination at an excitation wavelength of 638 nm [Excitation Beam Splitter: DD 488/552 and TD 488/552/638, Gain: 25.0 is same in 2 channels, Pinhole: 1.00 AU]

18. Generation of barcodes in a single image by varying ratios of CB[7]-FLs (1:0, 4:1, 1:1, 0:1)

1. U2OS cells were grown in 8 well imaging dish.

2. U2OS cells were methanol fixed and tagged with ^{C}ADA conjugated antibody as described previously.

3. Imaging was performed using Leica SP8 confocal system in PBS.

4. A whole cell was chosen as a ROI-1 using freehand drawing tool from CLSM toolbox.

5. FRAP module of the confocal system was used to carry out the photoactivation. The selected ROI-1 was irradiated using 405 nm laser (50% intensity, source power: 50 mW) for a period of 60s. Zoom in mode of bleaching was used with background set to zero for the photoactivation.

6. Incubation of 150 μL of CB[7]─Alexa 647 (100 nM) in PBS for a time interval of 5 min.

7. Washing with 200 μL PBS (3×).

8. Activation of ROI-2 (Cell no-2) using 405 nm laser with same laser power and time interval (similar to ROI-1).

9. Incubation of 150 μL of CB[7]─Alexa 647 + CB[7]─TAMRA (100 nM + 25 nM , Alexa647:TAMRA – 4:1) in PBS for a time interval of 5 min.

10. Washing with 200 μL PBS (3×).

11. Activation of ROI-3 (Cell no-3) and incubation of 150 μL of CB[7]─Alexa 647 + CB[7]─ TAMRA (100 nM + 100 nM , Alexa 647:TAMRA – 1:1) in PBS for a time interval of 5 min.

12. Washing with 200 μL PBS (3×).

13. Activation of ROI-4 (Cell no-4) and incubation of 150 μL of CB[7]─TAMRA (100 nM) in PBS for a time interval of 5 min.

14. Washing with 200 μL PBS (3×).

15. CLSM images were recorded with the same HyD detector. Image processing was done using ImageJ (Fiji) software where ROI manager was used to calculate the mean intensity in each region of interest (ROI) in red and green channel. The mean intensity obtained in each channel was normalized after subtracting the background intensity (from outside the cell) from each channel. Final ratio between green and red fluorescence colours were assigned by normalizing against single CB[7]-FL labelled region (i.e 1:0 or 0:1 ratio).

Note: The emission in green channel is collected at wavelength from 580-635 nm, upon illumination at an excitation wavelength of 552 nm (0.75 % laser intensity) and emission in red channel is collected at wavelength from 655 to 720 nm (0.75% laser intensity), upon illumination at an excitation wavelength of 638 nm. [Excitation Beam Splitter: DD 488/552 and TD 488/552/638, Gain: 25.0 is same in 2 channels, Pinhole: 1.00 AU]

19. Host-guest interaction mediate dual colour single actin filament labeling protocol in fixed Hela cells:

1. Removal of culture media from 8-well chamber slide system.
2. Fixation for 15 min with 200 μL 4% paraformaldehyde in PBS.
3. Washing with 200 μL PBS (3×).
4. Permeabilization with 200 μL 0.25% v/v Triton X–100 in PBS for 10 min.
5. Washing with 200 μL PBS (3×).
6. Blocking for 2 h with 200 μL 3% bovine serum albumin and 0.1% v/v Triton X–100 in PBS at room temperature.
7. Before staining, cells were washed with 200 μL PBS (3×).
8. Cells were stained for 2 h at room temperature with ^{C}ADA─phalloidin (2 μM) diluted in PBS.
9. Washing cells with 200 μL PBS (2×).
10. Incubation with 200 μL of CB[7]-fluorophore conjugate (100 nM) in PBS for 15. CB[7]─Alexa 647 was used for first labelling cycle.
11. A small part of the cell (towards the edge) was illuminated with 405 nm laser for activation of ^{C}ADA. 100 % output from 405 nm laser was used from SIM set-up for 90 s to activate the ^{C}ADA.
12. After 1st color-coding excess fluorophores were removed after 15 min of photoactivation and the second colour coding step was started. Irradiation to an adjacent region using 405

nm laser was performed for 90 s. 2nd cycle colour coding was performed using a CB[7]–Cy3 fluorophore.

13. Excess fluorophore was washed after 15 min of 2nd photoactivation. Structured illumination images were recorded in both Alexa 647 and Cy3 channel after washing.

20. Host−Guest mediated spatiotemporal live cell glycan labelling protocol:

1. Culture media was removed from the chamber well and HEK cells were washed twice in DPBS (pH 7.3).
2. Cells were incubated with $Ac_4ManNAz$ (25 µM) at 37°C.
3. Three days after incubation, cells were washed three times with cold DPBS (pH 7.3).
4. Immediately after washing, the cells were incubated with 25 µM ^{C}ADA─DBCO in complete cell culture DMEM media with FBS for 2 h at 37°C.
5. Cells were then washed five times with DPBS (pH 7.3).
6. 25 nM CB[7]─TAMRA fluorophore in complete cell culture DMEM media with FBS was added. Cells were incubated with the imager for 10 min at 37°C in a humid atmosphere (5% CO_2).
7. Region of interest (ROI) based activation of ^{C}ADA tagged sialic acid bearing cell-surface glycans were done using 405 nm laser light for 90 s (80% of 30 mW source power).
8. Excess imager was removed by washing with DBPS and cells were imaged by keeping them in phenol red free culture medium.
9. For time-lapsee imaging, cells were kept at 37°C in a humid atmosphere (5% CO_2) by using incubator attached to confocal microscope.

[Experimental parameters – Laser: 561 nm with 2.5 % power, Detection wavelength: 562-625 nm, Digital gain : 1.00, Pinhole: 0.85 AU]

21. Temporal monitoring of CB[7]-fluorophore labelled glycans:

1. Culture media was removed from the chamber well and MCF7 cells were washed twice in DPBS (pH 7.3).
2. Cells were incubated with $Ac_4ManNAz$ (20 µM) at 37°C.
3. Three days after incubation, cells were washed three times with cold DPBS (pH 7.3).
4. Immediately after washing, the cells were incubated with 10 µM ^{C}ADA─DBCO in complete cell culture DMEM media with FBS for 2 h at 37°C.
5. Cells were then washed five times with DPBS (pH 7.3).

6. Cells were photoirradiated using 365 nm LED light (50 mW/cm^2 emitted light from the handheld LED source) for 90 s.
7. 100 nM of CB[7]-Alexa 647 in complete cell culture DMEM media with FBS was added. Cells were incubated with the imager for 15 min at 37°C in a humid atmosphere (5% CO_2).
8. Excess imager was removed by washing with DBPS and cells were imaged by keeping them in phenol red free culture medium.
9. For time-lapsee SIM imaging, cells were kept at 37°C in a humid atmosphere (5% CO_2) by using incubator attached to microscope.

22. Long-term monitoring of glycans in live cells:

1. Culture media was removed from the chamber well and MCF7 cells were washed twice in DPBS (pH 7.3).
2. Cells were incubated with Ac_4ManNAz (20 µM) at 37°C.
3. Three days after incubation, cells were washed three times with cold DPBS (pH 7.3).
4. Immediately after washing, the cells were incubated with 10 µM ^{C}ADA–DBCO in complete cell culture DMEM media with FBS for 2 h at 37°C.
5. Cells were then washed five times with DPBS (pH 7.3).
6. Cells were photoirradiated using 365 nm LED light (50 mW/cm^2 emitted light from the handheld LED source) for 90 s.
7. 100 nM of CB[7]-Fluorescein in complete cell culture DMEM media with FBS was added. Cells were incubated with the imager for 15 min at 37°C in a humid atmosphere (5% CO_2).
8. Excess imager was removed by washing with DBPS and cells were kept in phenol red free culture medium. Cells were then imaged at a time point of 6 h, 12 h, 24 h and 48 h.

Note: During the process of experiment, cells were stored in an incubator which was maintained at 37°C under humid atmosphere (with 5% CO_2).

23. Host- guest interaction mediated spatiotemporal labelling of live A549 cells in a co-culture with MEF cells.

1. MEF and A549 cells were individually cultured in a humidified atmosphere (5% CO_2) at 37°C and grown in Dulbecco's Modified Eagle's Medium (DMEM, high glucose) supplemented with 10% fetal bovine serum (FBS) (Gibco, USA), 2 mM Glutamax

(Invitrogen, USA) and 1% antibiotics (100 U/mL penicillin, 100 μg/mL streptomycin and 0.25 μg/mL amphotericin) (Gibco, USA).

2. At ~ 80% confluence, both the cells were washed with DPBS (pH 7.4) (Gibco, USA), trypsinized and suspended in culture medium.
3. Cells were then counted and ~4,000 cells of MEF and ~4,000 cells of A549 cells were suspended in 200 μL of cell culture media (MEF:A549=1:1). This cell suspension was placed in a 35 mm imaging dish. This co-culture of two cells in the glass bottom dish was kept for overnight in the incubator.
4. Before experiment, culture media was removed carefully from the glass dish and cells were washed twice in DPBS (pH 7.4).
5. Cells were incubated with 100 μL primary antibody against EGFR (human, 10 μg mL^{-1}) in DPBS for 20 min at 4°C.
6. Excess antibody was removed, and cells were washed 2 times with 100 μL DPBS (pH 7.4).
7. Then cells were incubated with 100 μL ^{C}ADA and BODIPY conjugated donkey anti–human 2° antibody (10 μg.mL^{-1}) in DPBS for 20 min at 4°C.
8. After subsequent removal of excess 2° antibody with DPBS wash, cells were imaged using the Leica SP8 confocal system in DPBS.
9. Two ROIs having circular area of 30 x 30 μm were selected using the CLSM toolbox.
10. FRAP module of the confocal system was used to carry out the photoactivation. The selected ROI-1 and ROI-2 was irradiated together using 405 nm laser (40% intensity, source power: 50 mW) for a period of 60 s. Zoom in mode of bleaching was used with background set to zero for the photoactivation.
11. Incubation of 100 μL of CB[7]─Alexa 647 (100 nM) in DPBS for a time interval of 5 min.
12. Washing with 100 μL DPBS (2×).
13. CLSM images were recorded with the same HyD detector. Image processing was done using ImageJ (Fiji) software.

Note: The emission in green channel is collected at wavelength from 500-545 nm, upon illumination at an excitation wavelength of 488 nm (2 % laser intensity) and emission in red channel is collected at wavelength from 655 to 720 nm (4% laser intensity), upon illumination at an excitation wavelength of 638 nm [Excitation Beam Splitter: DD 488/552 and TD 488/552/638, Gain: 100.0 is same in 2 channels, Pinhole: 1.00 AU].

Synthesis of ^{C}ADA and BODIPY conjugated donkey anti-Human antibody:

1. Secondary antibody (Donkey Anti-Human) was buffer exchanged by Zeba spin column pre–equilibrated with PBS containing 10% 1M $NaHCO_3$.

2. The concentration of the antibody was measured and used for the conjugation with ^{C}ADA–TEG–NHS-B (Compound J).

3. Buffer exchanged secondary antibody (82.0 μg, 5.46 x10^{-4} μmol) was first taken in 1.5 mL autoclaved micro centrifuge tube.

4. 1.1 μL solution (Conc:10 mg.mL^{-1} in DMSO) of ^{C}ADA–TEG–NHS-B (Compound J) and 0.85 μL solution of BODIPY-NHS (Conc.: 0.5 mg.mL^{-1} in DMSO) were diluted to 5 μL by DMSO (3.67 μL) and added to the antibody solution in two portions (2.5 μL each time).

5. The reaction was kept at 4°C for overnight.

6. ^{C}ADA and BODIPY conjugated donkey anti-human antibody was then purified by Zeba spin column (pre–equilibrated with PBS) and the concentration was measured.

24. Host-guest interaction mediated multiple barcoding in MEF cellular region:

1. Removal of culture media.
2. Washing cells with 200 μL PBS (2×).
3. Fixation for 7 minutes with 200 μL chilled methanol at −20°C followed by washing for three times with PBS.
4. Blocking for 2 h with 200 μL 3% bovine serum albumin in PBS at room temperature.
5. Incubation for 24 h at 4°C with primary antibody (10 μg.mL^{-1}, 200 μL) diluted in PBS containing 3% bovine serum albumin.
6. Washing with 200 μL PBS (3×) with 5 min incubation each time.
7. Incubation for 12 h at 4°C with ^{C}ADA conjugated secondary antibody (10 μg.mL^{-1}, 200 μL) in 3% bovine serum albumin and 0.1 % triton x in PBS at room temperature.
8. Washing with 200 μL PBS (3×) with 5 min incubation each time to remove the excess ^{C}ADA conjugated secondary antibody.
9. Selection of ROIs using the CLSM toolbox at ten different regions each having square area of 40 x 40 μm.
10. Incubation of 200 μL of CB[7]─ Fluorophore-1 (CB[7]─Alexa647, 100 nM) in PBS.

11. Activation of ROI using 405 nm laser with different laser power and time interval as shown in Scheme S9.
12. Washing cells with 200 μL PBS (2×) 5 min after irradiation.
13. Incubation of 200 μL of CB[7]─ Fluorophore-2 (CB[7]─ Fluorescein, 100 nM) in PBS.
14. Activation of ROI using 405 nm laser with laser power and time interval as shown in in Scheme S9.
15. Washing cells with 200 μL PBS (2×) 5 min after irradiation.
16. Incubation of 200 μL of CB[7]─ Fluorophore-3 (CB[7]─ TAMRA, 100 nM) in PBS.
17. Activation of ROI using 405 nm laser with different laser power and time interval as shown in Scheme S9.
18. Washing cells with 200 μL PBS (2×) 5 min after irradiation.
19. CLSM images were recorded in three channels (638 nm, 552 nm, and 488 nm) with the same HyD detector.

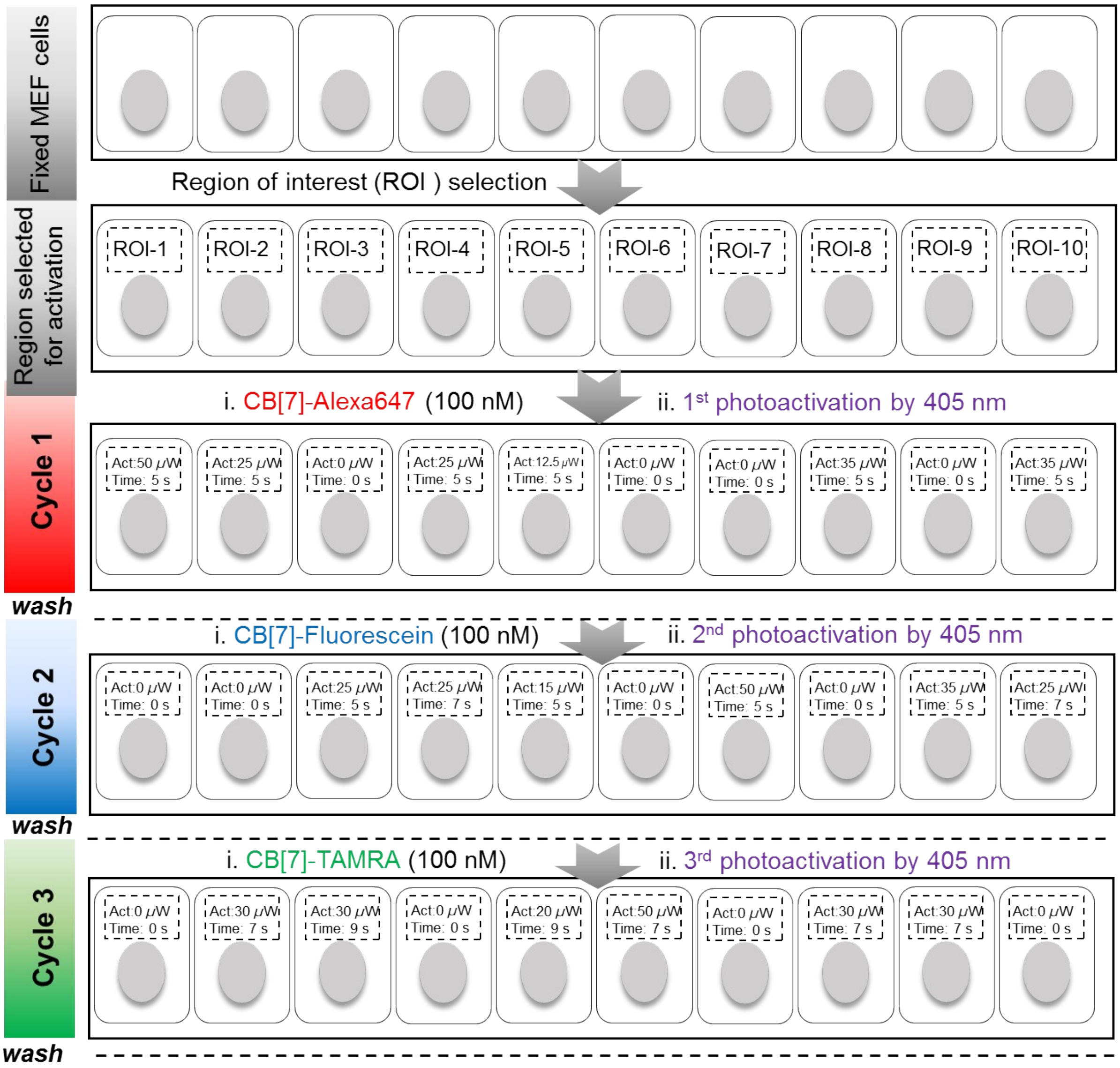

Scheme S9: Schematic of the light-activated cellular barcoding by controlling degree of ^{C}ADA photoactivation. α-tubulin of MEF cell is tagged with photoresponsive ^{C}ADA–antibody for barcoding**.** Each cycles represents photoactivation with variable laser power and time-interval (shown inside the ROI), followed by assembly with three unique color coded CB[7]-fluorophores (Alexa, Fluorescein and TAMRA) to generate 10-barcodes in 3 cycle.

Note: The laser power for 405 nm is around 0.5 mW (Leica TCS SP8). The region of interest (ROI) photoactivated for barcoding is over 1600 μm^2, which is constant for each barcodes. The emission in blue channel is collected at wavelength from 495 to 540 nm, upon illumination at an excitation wavelength of 488 nm (2% laser intensity), while the emission in green channel is collected at wavelength from 575-625 nm, upon illumination at an excitation wavelength of 552 nm (2% laser intensity) and finally the emission in red channel is collected at wavelength from 645 to 705 nm (2% laser intensity), upon illumination at an excitation wavelength of 638 nm.

25. Host−Guest mediated multiple barcoding on live cell glycan of MCF7 cells:

1. Culture media was removed from the chamber well and MCF7 cells were washed twice in DPBS (pH 7.3).
2. Cells were incubated with $Ac_4ManNAz$ (20 μM) at 37°C.
3. Three days after incubation, cells were washed three times with cold DPBS (pH 7.3).
4. Immediately after washing, the cells were incubated with 10 μM ^{C}ADA-DBCO in DMEM for 2 h at 37°C.
5. Cells were then washed two times with DPBS (pH 7.3).
6. Selection of ROIs using the CLSM toolbox at seven different regions each having square area of 70 x 70 μm.
7. Incubation of 200 μL of CB[7]─ Fluorophore-1 (CB[7]─Alexa647, 100 nM) in DPBS.
8. Activation of ROI using 405 nm laser with different laser power as shown in Scheme S10.
9. Washing cells with 200 μL DPBS (2×) 5 min after irradiation.
10. Incubation of 200 μL of CB[7]─ Fluorophore-2 (CB[7]─ Fluorescein, 100 nM) in DPBS.
11. Activation of ROI using 405 nm laser with different laser power as shown in Scheme S10.
12. Washing cells with 200 μL DPBS (2×) 5 min after irradiation.
13. Incubation of 200 μL of CB[7]─ Fluorophore-3 (CB[7]─ TAMRA, 100 nM) in DPBS.
14. Activation of ROI using 405 nm laser with different laser power as shown in Scheme S10.
15. Washing cells with 200 μL DPBS (2×) 5 min after irradiation.
16. CLSM images were recorded in three channels (638 nm, 552 nm, and 488 nm) with the same HyD detector.

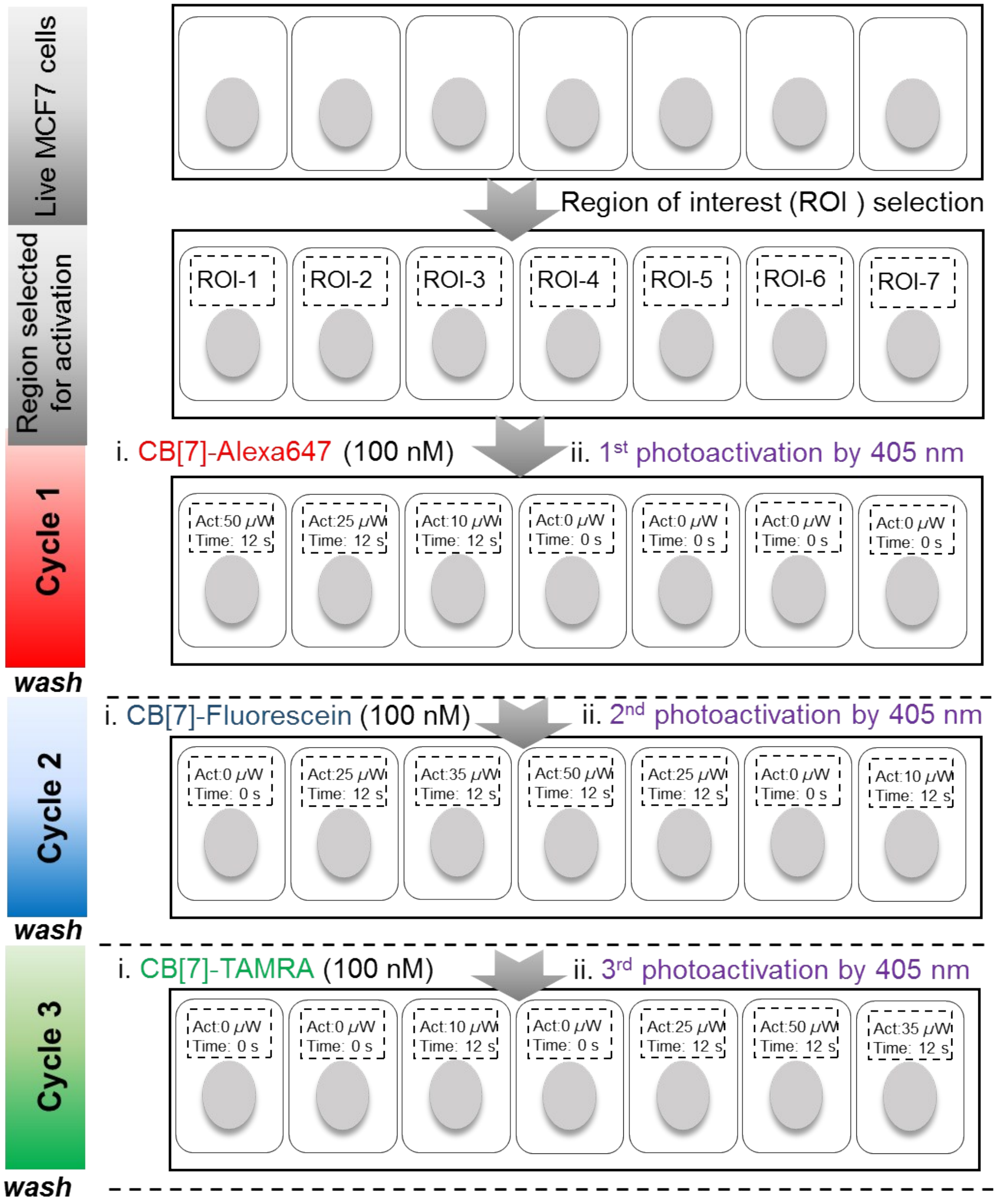

Scheme S10: Schematic of the light-activated cellular barcoding in live cell by controlling degree of ^{C}ADA photoactivation. Cell surface sialic acid is tagged with photoresponsive ^{C}ADA for barcoding**.** Each cycles represents photoactivation with variable laser power (shown inside the ROI), followed by assembly with three unique color coded CB[7]-fluorophores (Alexa, Fluorescein and TAMRA) to generate 7-barcodes in 3 cycle in live MCF7 cells.

Note: The laser power for 405 nm is around 0.5 mW (Leica TCS SP8). The region of interest (ROI) photoactivated for barcoding is over 4900 μm^2, which is constant for each barcodes.

The emission in blue channel is collected at wavelength from 495 to 540 nm, upon illumination at an excitation wavelength of 488 nm (4% laser intensity), while the emission in green channel is collected at wavelength from 575-625 nm, upon illumination at an excitation wavelength of 552 nm (2% laser intensity) and finally the emission in red channel is collected at wavelength from 645 to 705 nm (2% laser intensity), upon illumination at an excitation wavelength of 638 nm.

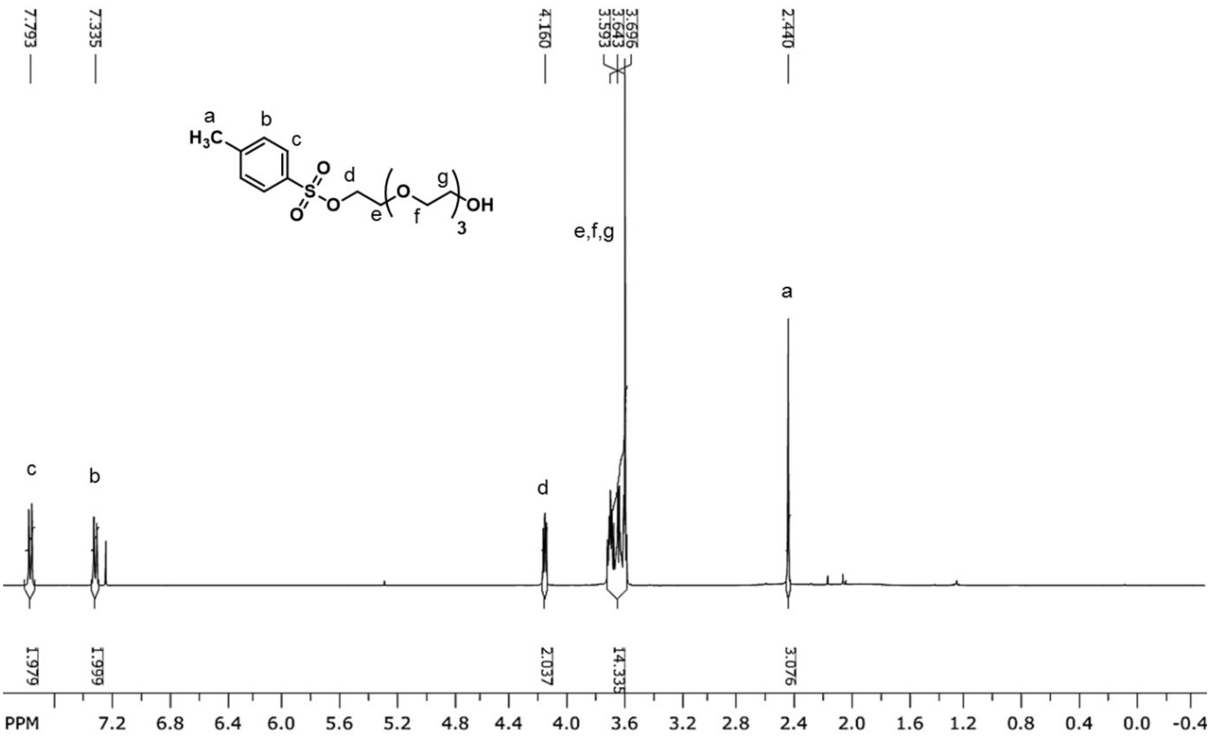

Figure S1. ^{1}H NMR spectrum of Compound A in $CDCl_3$ (400 MHz).

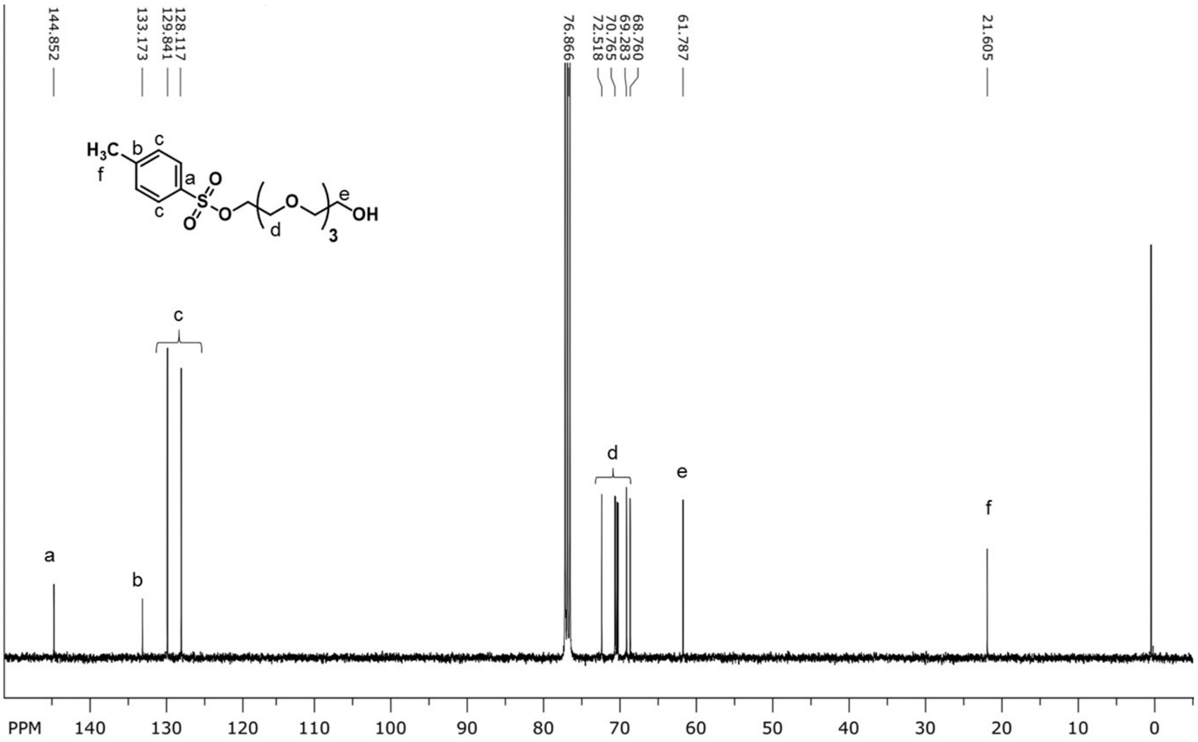

Figure S2. ^{13}C NMR spectrum of Compound A in $CDCl_3$ (400 MHz).

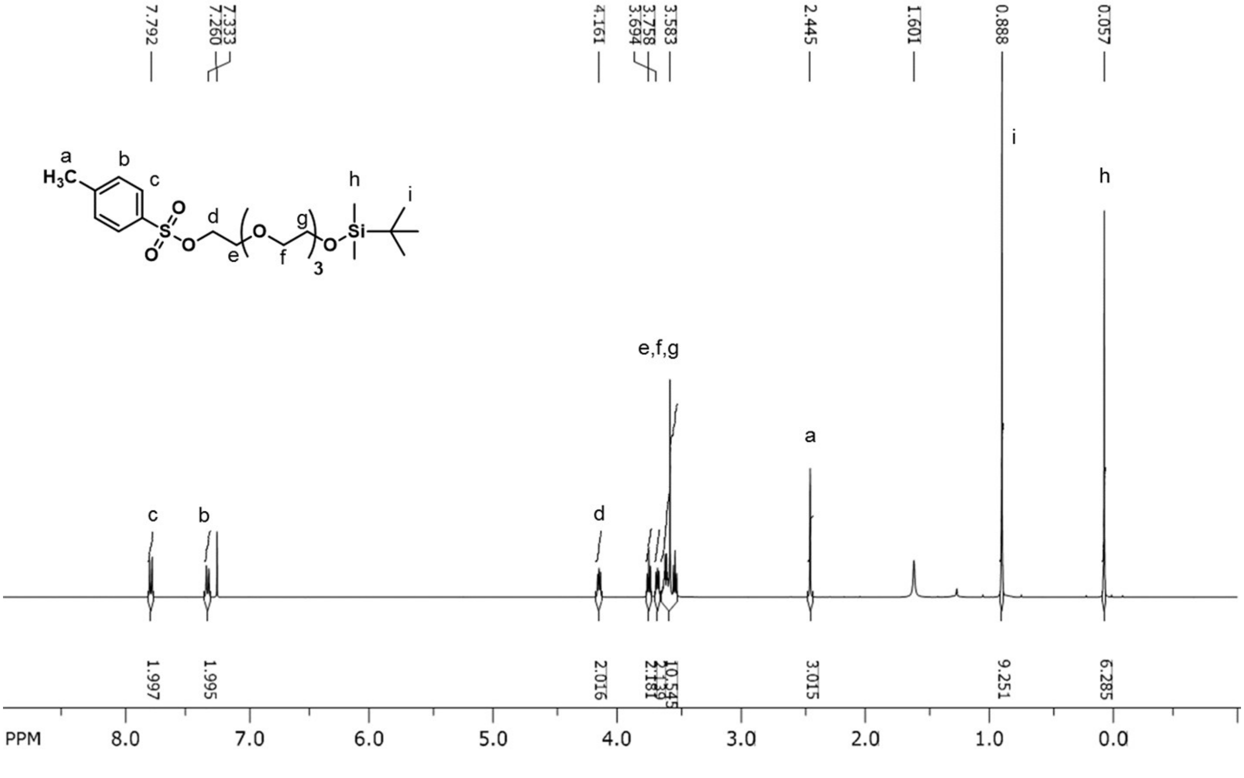

Figure S3. ^{1}H NMR spectrum of Compound B in $CDCl_3$ (400 MHz).

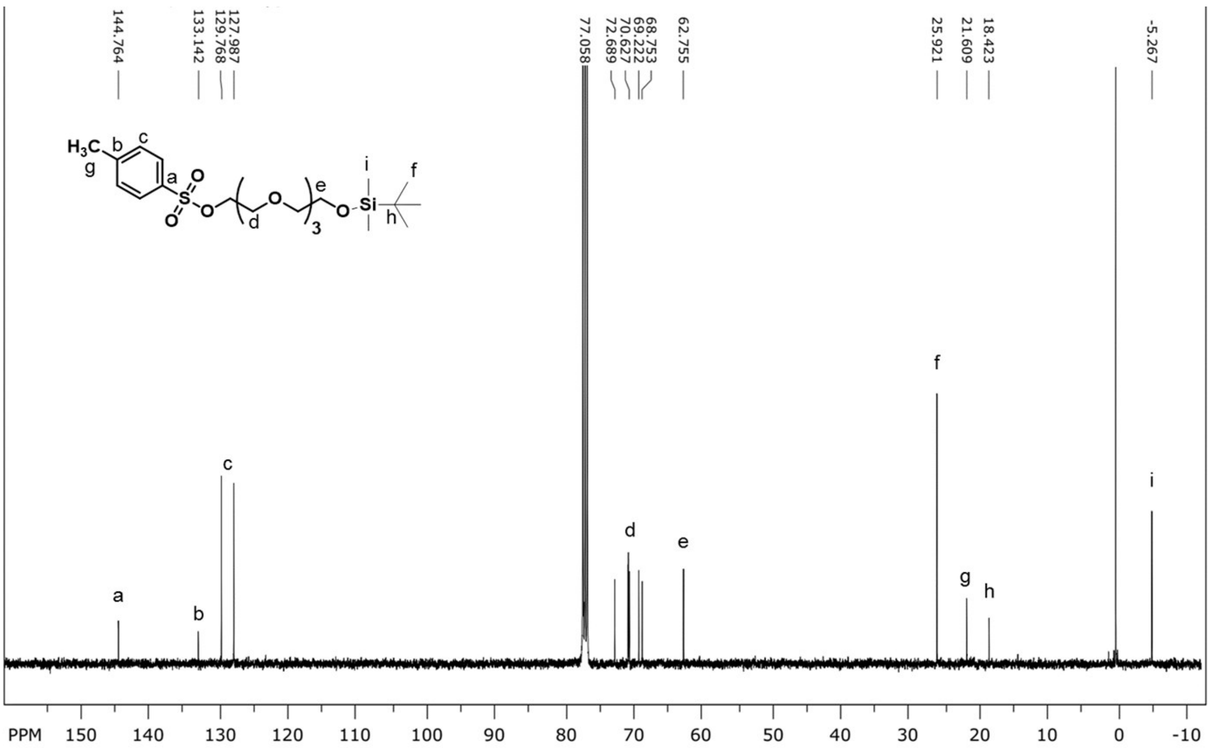

Figure S4. ^{13}C NMR spectrum of Compound B in $CDCl_3$ (400 MHz).

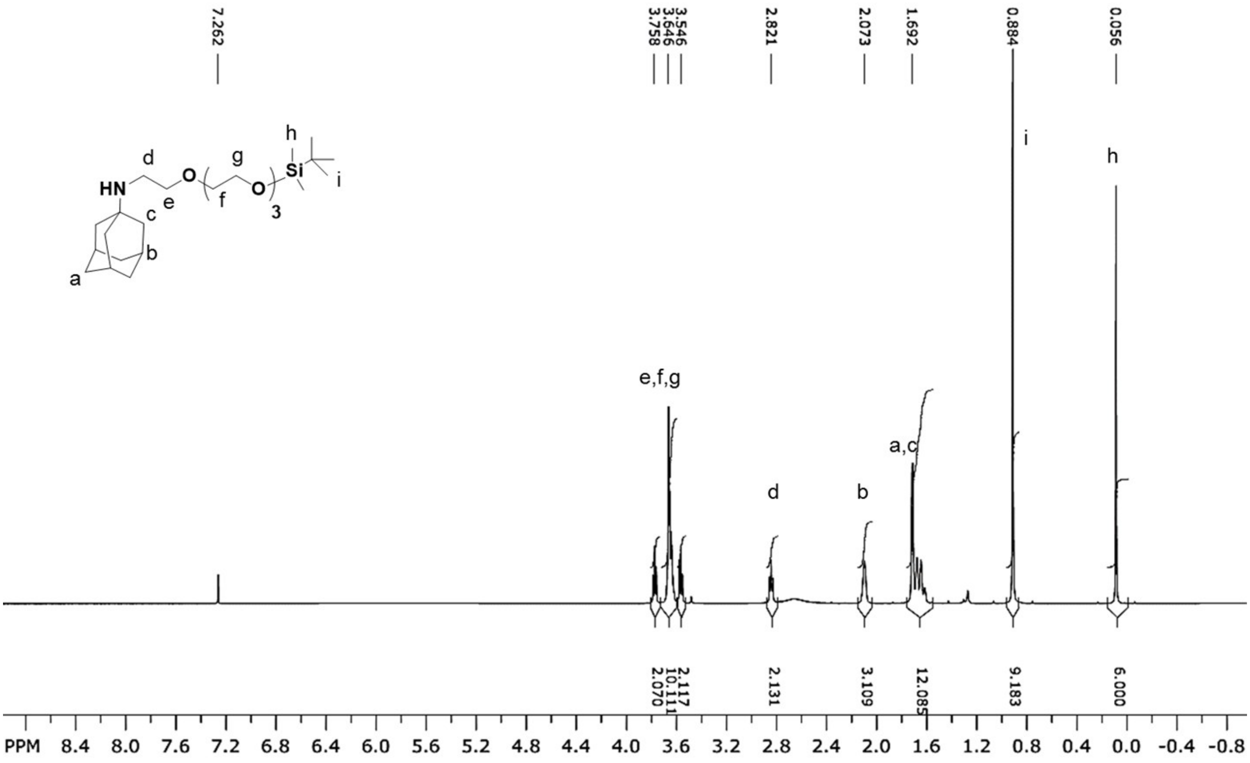

Figure S5. ^{1}H NMR spectrum of Compound C in $CDCl_3$ (400 MHz).

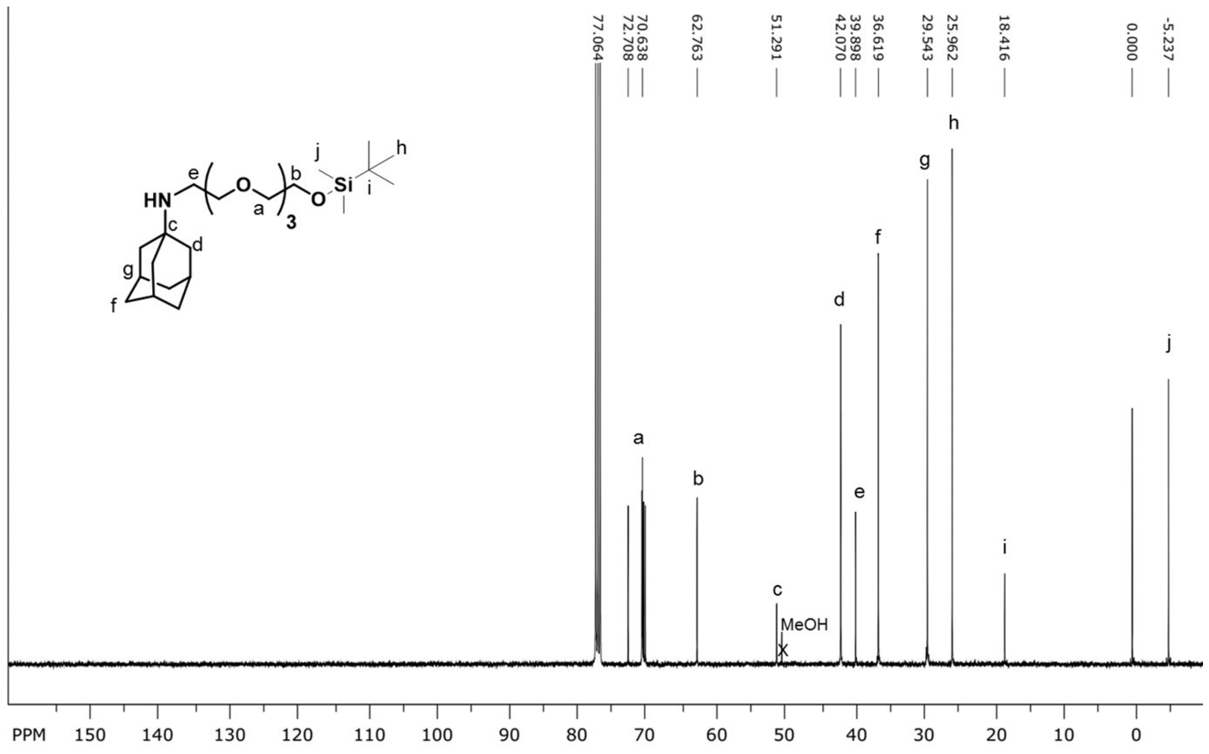

Figure S6. ^{13}C NMR spectrum of Compound C in $CDCl_3$ (400 MHz).

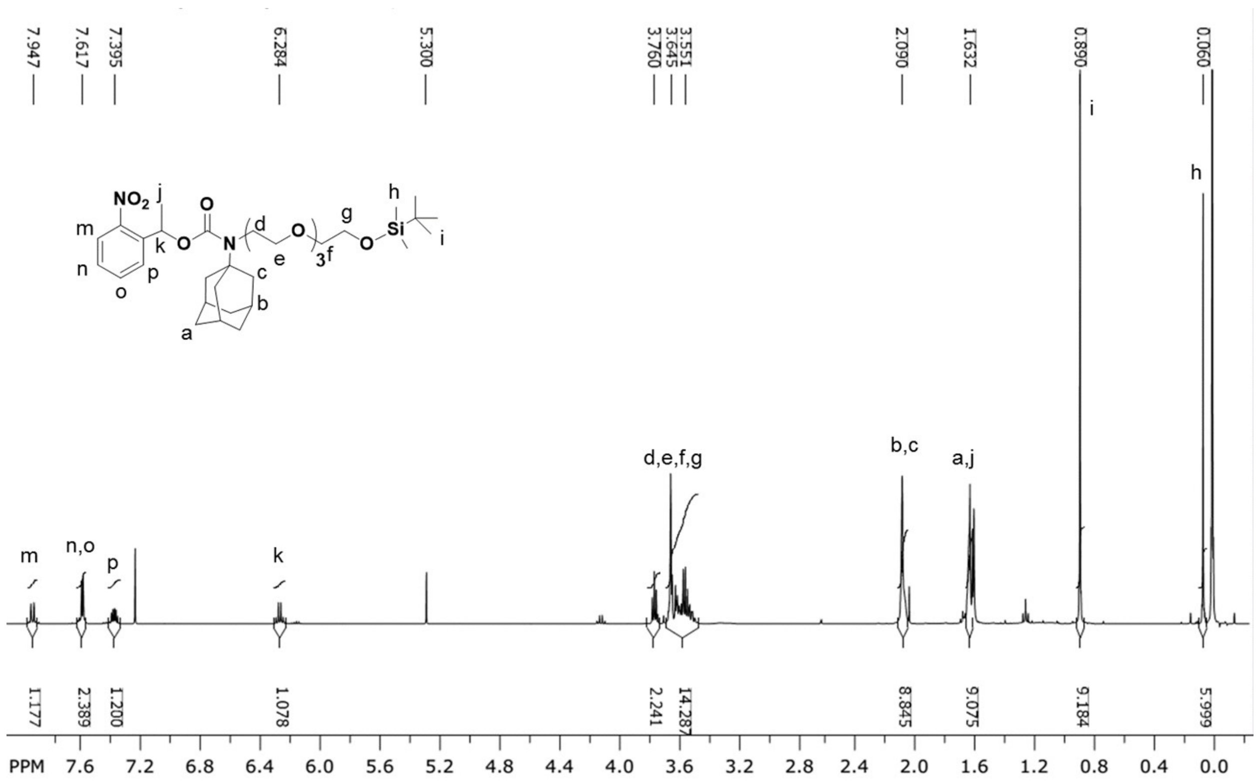

Figure S7. ^{1}H NMR spectrum of Compound D in $CDCl_3$ (400 MHz).

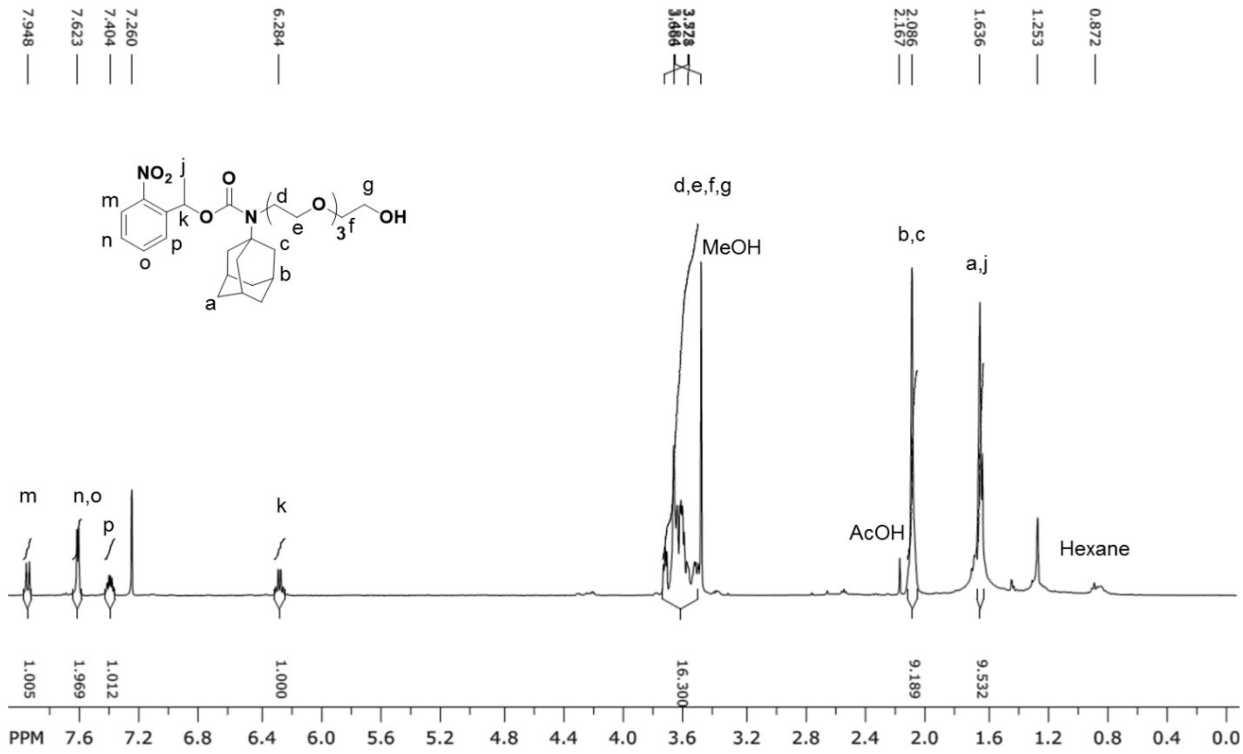

Figure S8. ^{1}H NMR spectrum of Compound E in $CDCl_3$ (400 MHz).

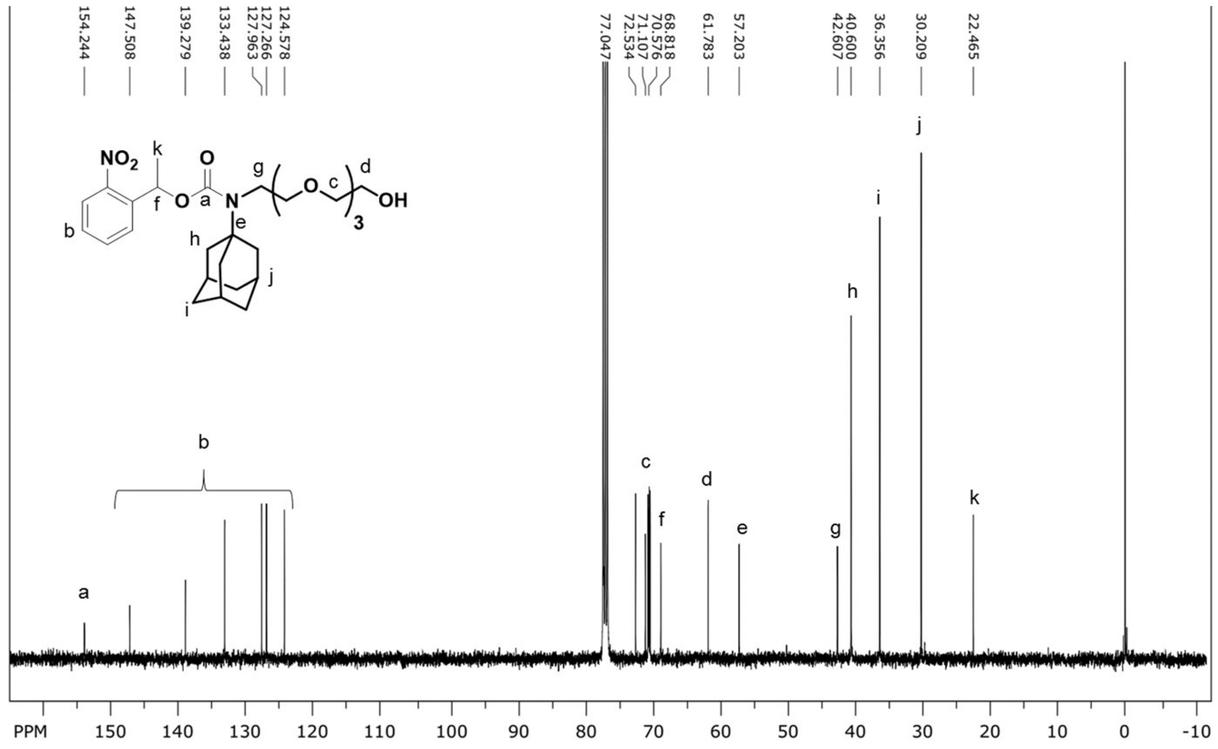

Figure S9. ^{13}C NMR spectrum of Compound E in $CDCl_3$ (400 MHz).

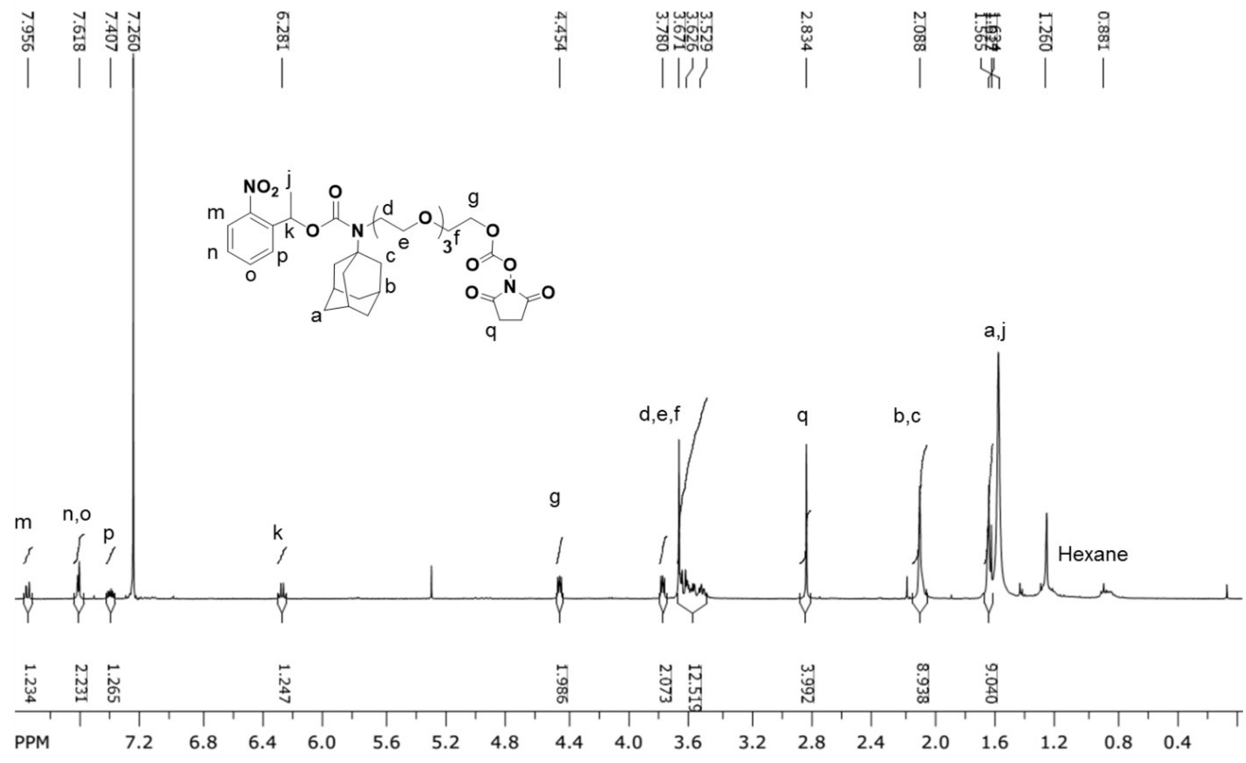

Figure S10. ^{1}H NMR spectrum of Compound F in $CDCl_3$ (400 MHz).

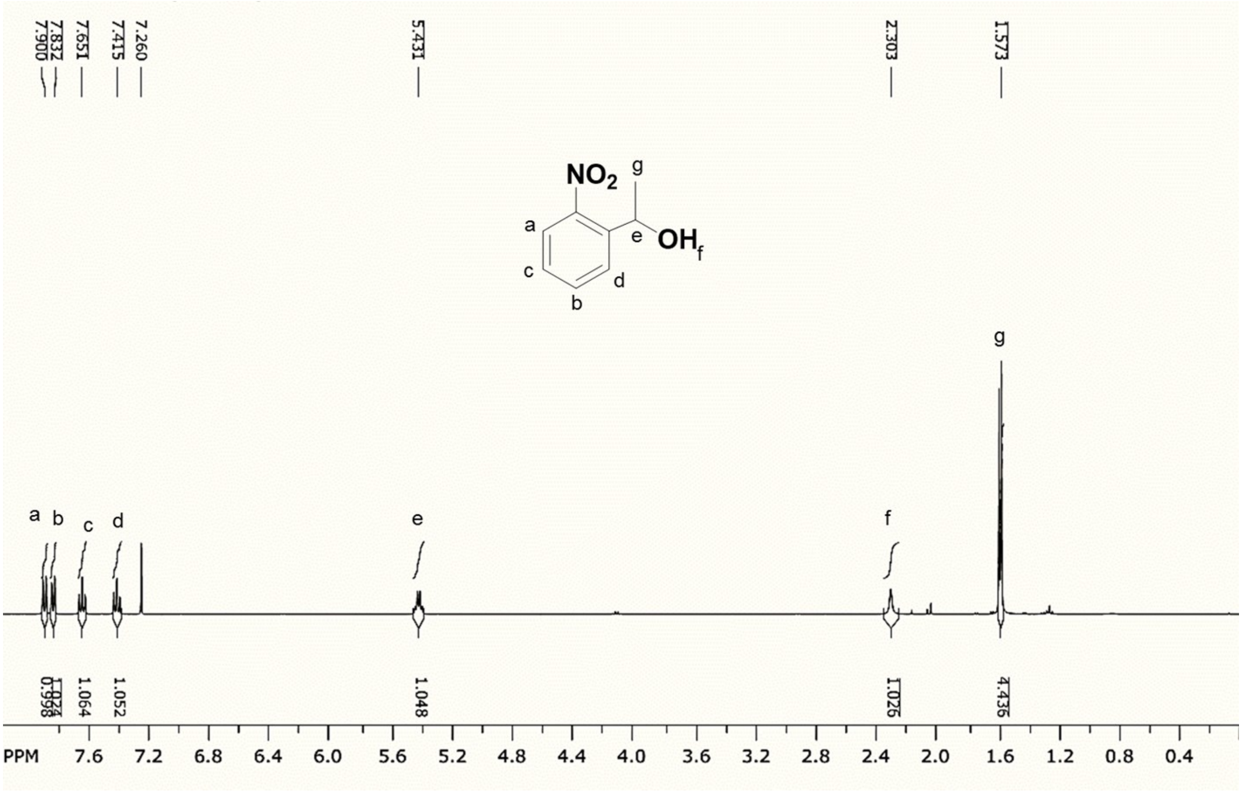

Figure S11. ^{1}H NMR spectrum of Compound G in $CDCl_3$ (400 MHz).

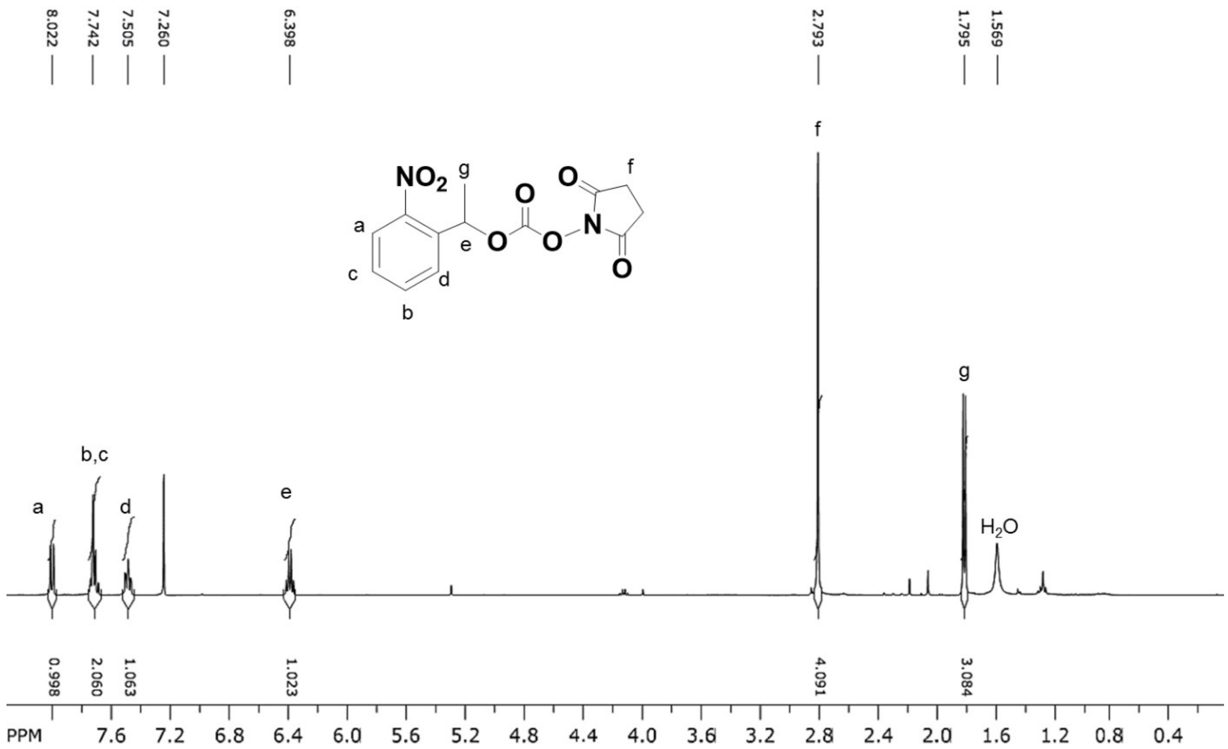

Figure S12. ^{1}H NMR spectrum of Compound H in $CDCl_3$ (400 MHz).

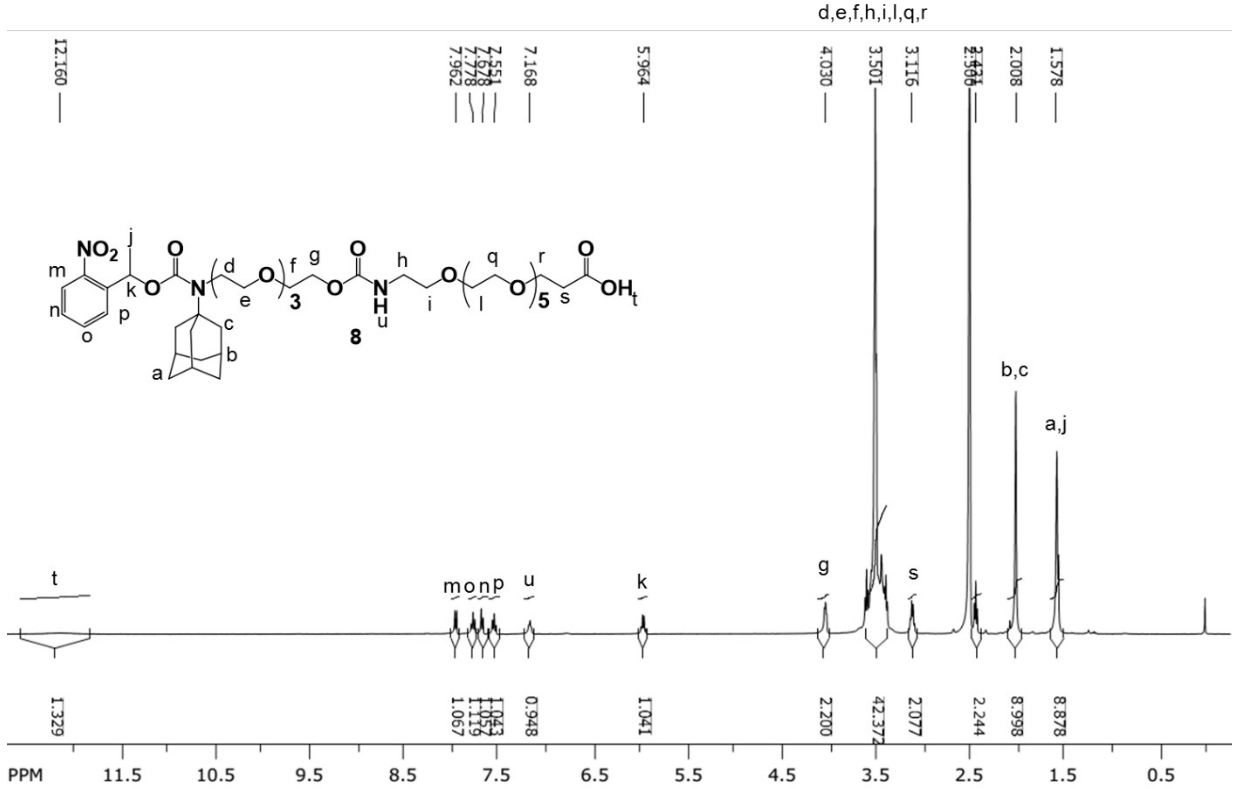

Figure S13. ^{1}H NMR spectrum of Compound I in DMSO-d6 (400 MHz).

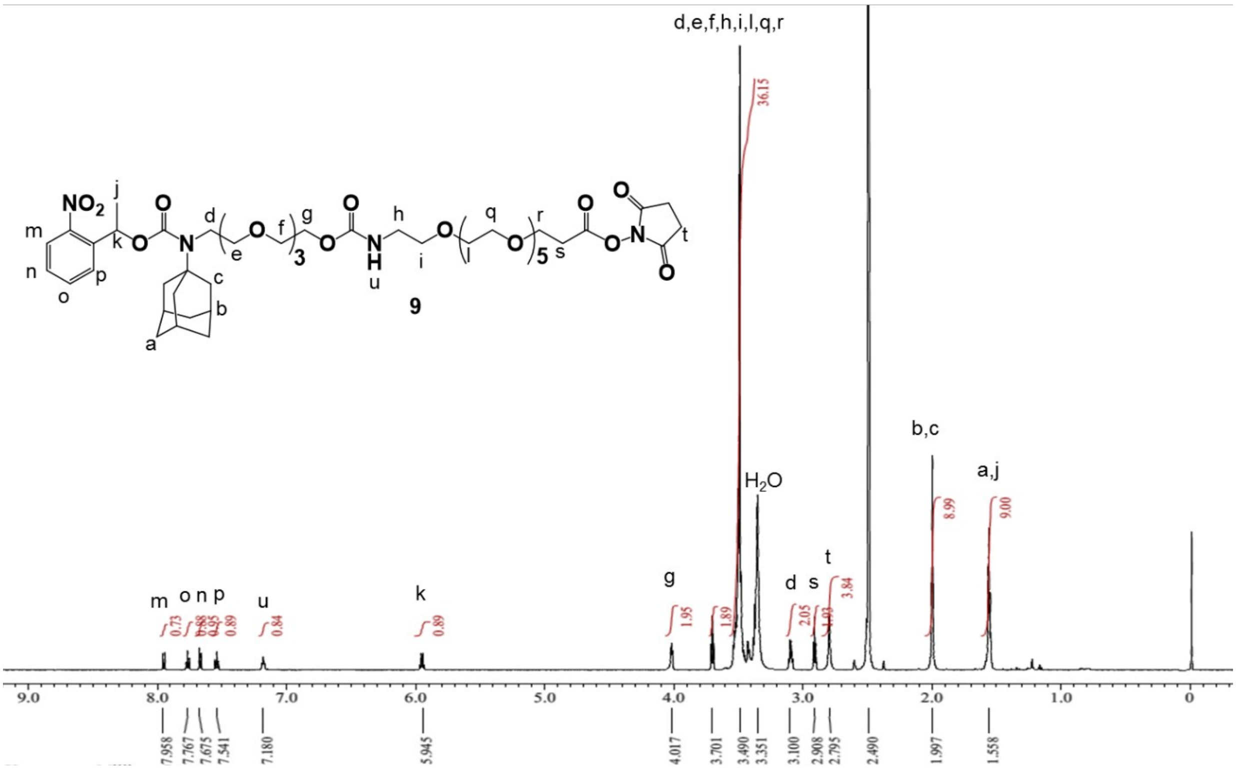

Figure S14. 1H NMR spectrum of Compound J in DMSO-d6 (600 MHz).

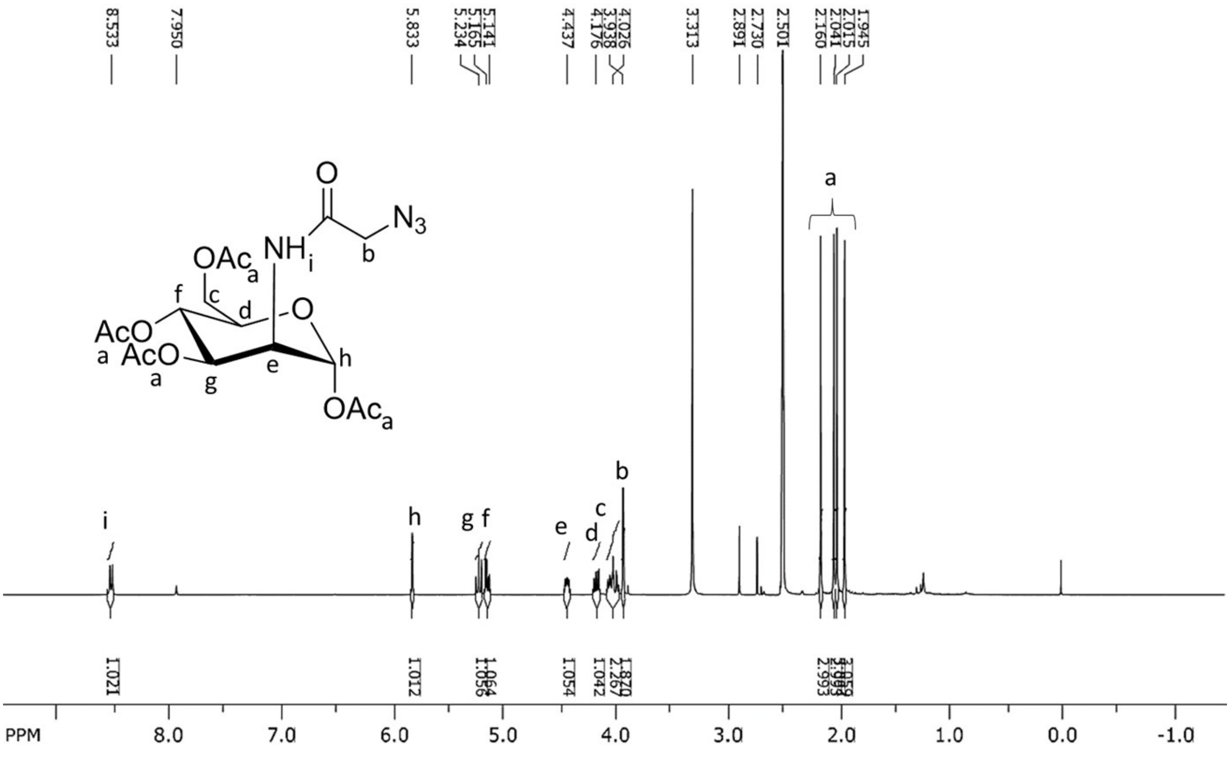

Figure S15. ^{1}H NMR spectrum of compound K in DMSO-d6 (400 MHz).

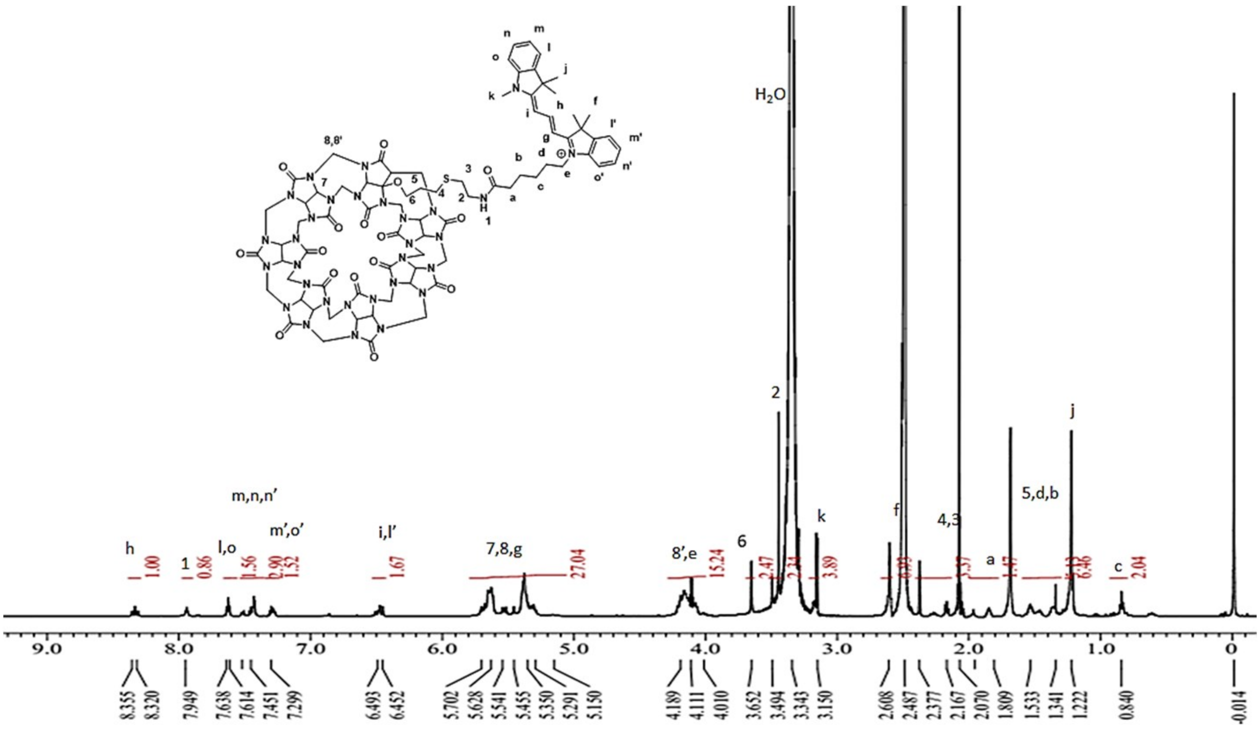

Figure S16: ^1H NMR spectrum of CB[7]–Cy3 conjugate

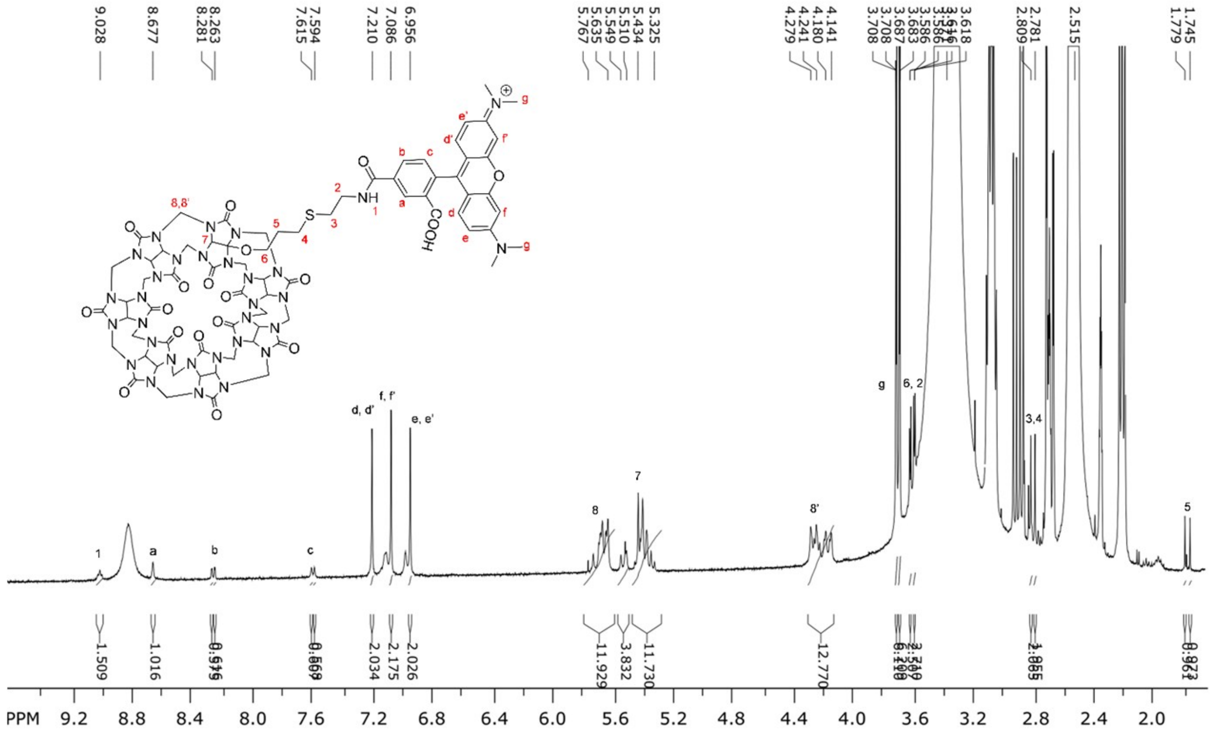

Figure S17: ^{1}H NMR spectrum of CB[7]–TAMRA conjugate

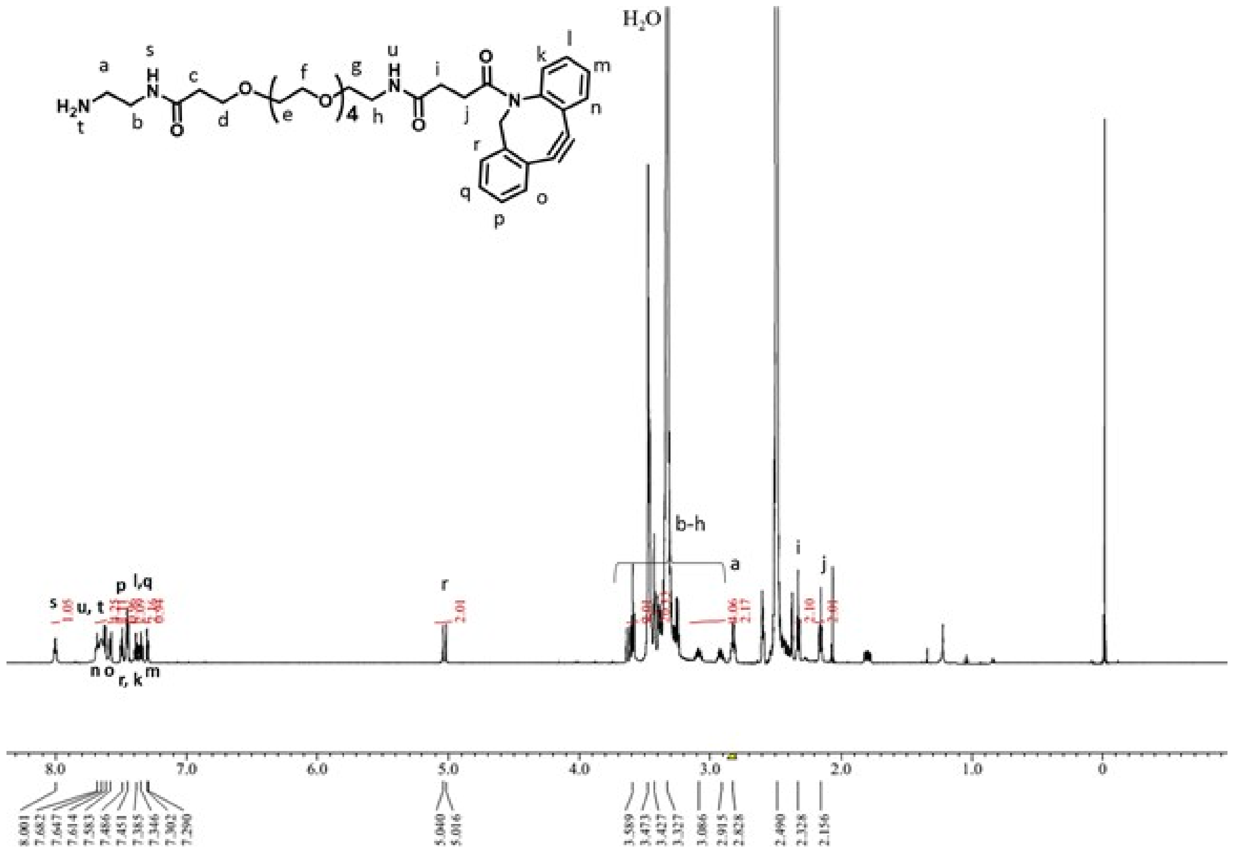

Figure S18. ^{1}H NMR spectrum of DBCO-diamine in DMSO-d6 (600 MHz).

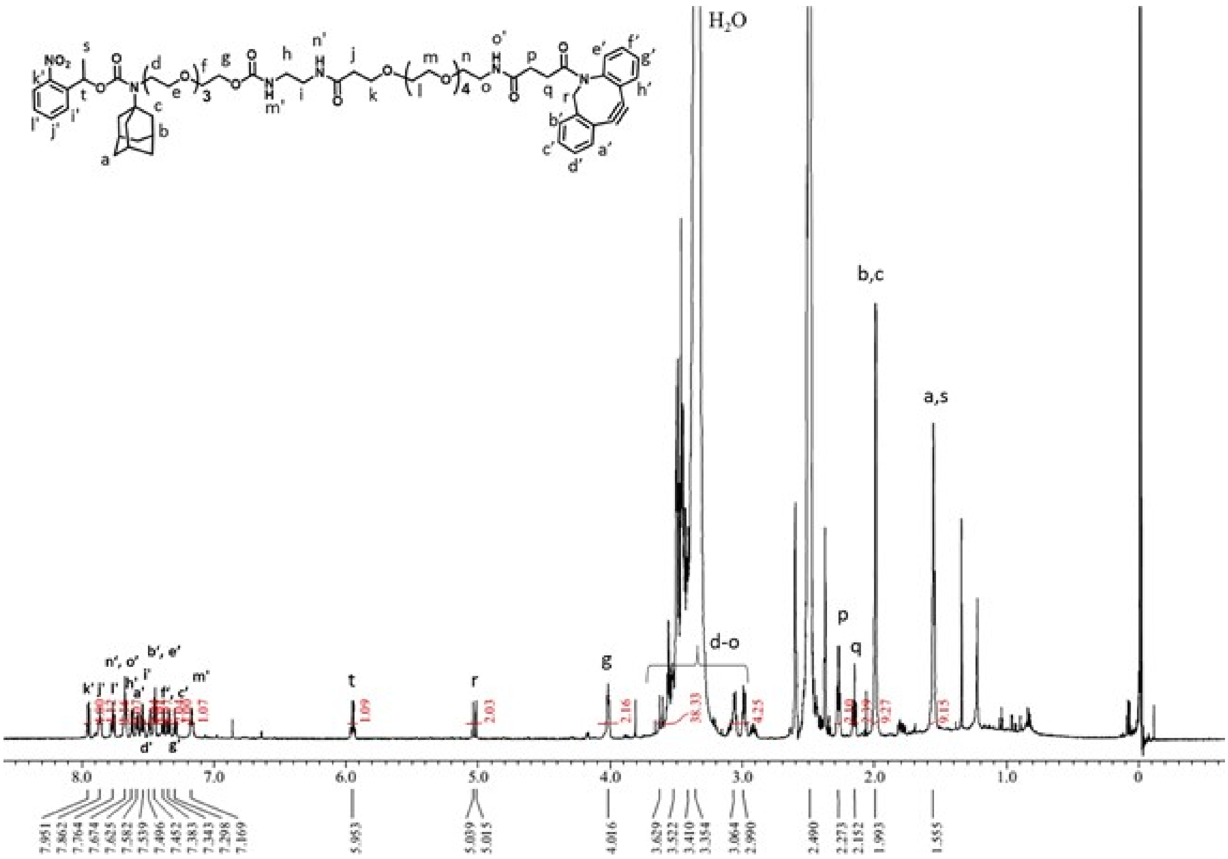

Figure S19. ^{1}H NMR spectrum of ^{C}ADA–DBCO in DMSO-d6 (600 MHz).

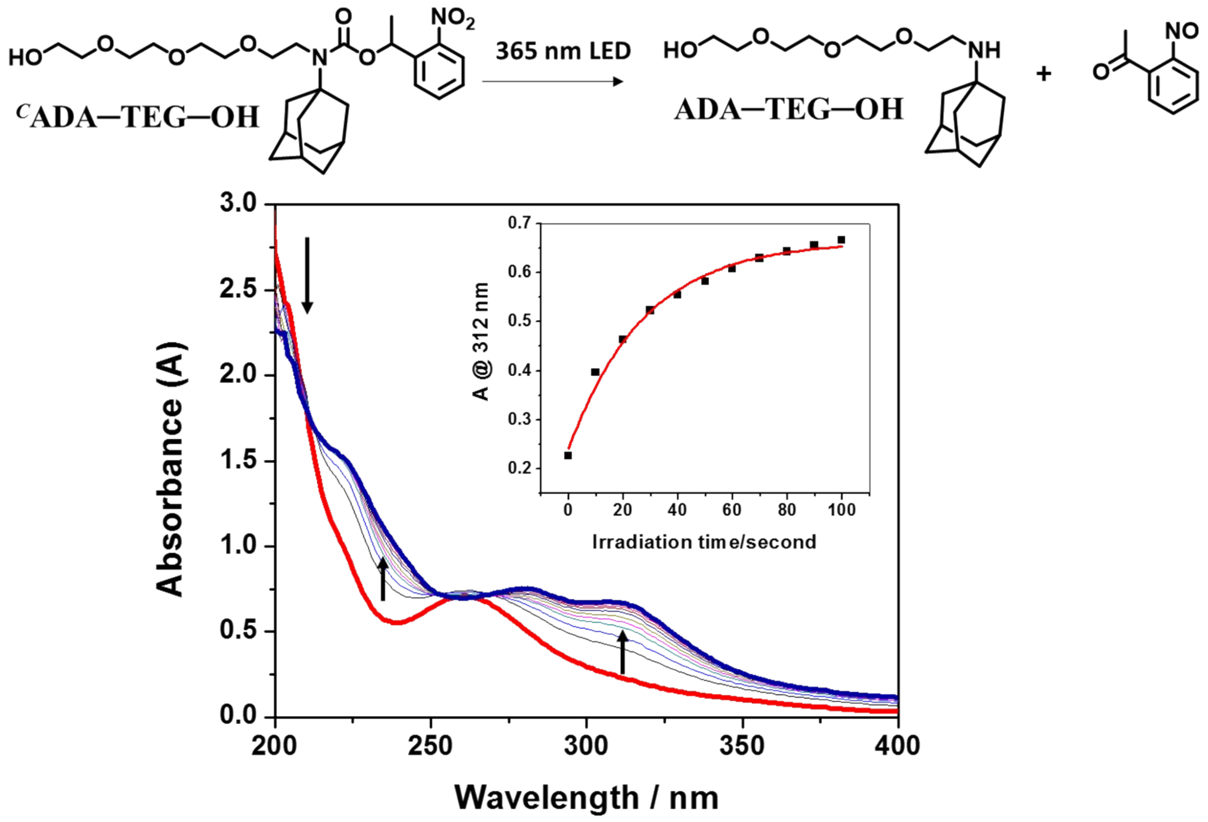

Figure S20. UV-Vis spectroscopic changes of ^{C}ADA–TEG–OH (100 μM) upon irradiation

with different duration of 365 nm LED light illumination (each spectrum taken at an interval of 10 s). The inset shows the variation in absorbance at 312 nm with irradiation time.

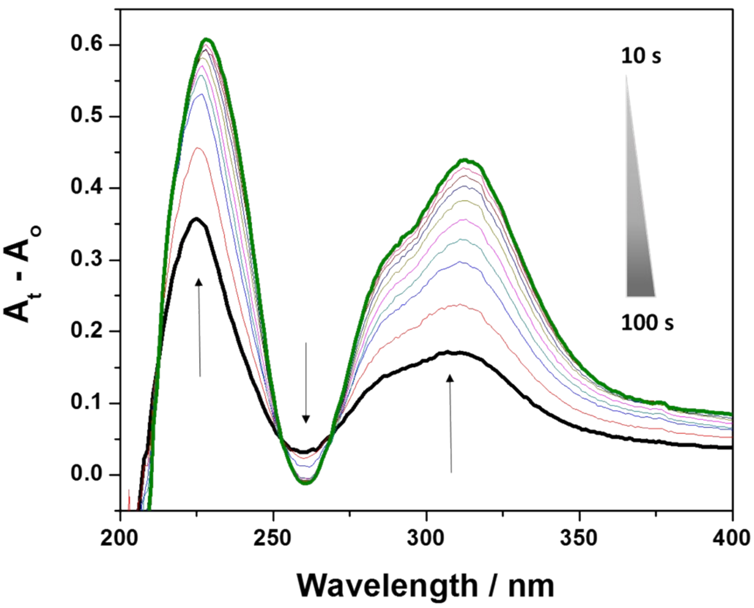

Time differential absorption profiles.

Figure S21. Differential absorption profile of ^{C}ADA—TEG—OH upon irradiation with UV light (λ = 365 nm, irradiation using 50 mW/cm^2 emitted light from a LED source).

Photochemical conversion of the ^{C}ADA-derivatives probed by LCMS

Photo-cleavage experiment was carried out for the ^{C}ADA-derivatives. High-performance liquid chromatography (HPLC) was carried out to observe the change in the retention time before and after irradiation (365 nm LED for 5 min, 50 mW/cm^2 light emission). The isolated compound was mass characterised (ESI mode in LCMS) to determine its change in molecular weight before and after irradiation. The HPLC chromatogram was obtained at 254 nm. The retention time and mass change are as follows:

Supporting Table 4: HPLC and LCMS of ^{C}ADA-derivatives before and after irradiation

^{C}ADA-Derivatives	Before Irradiation		After Irradiation	
	R_t	Mass	R_t	Mass
a. ^{C}ADA-Phalloidin	20.8	1334.86	12.8	1141.71
b. ^{C}ADA- SCy5	22.0	1231.76	17.0	1038.75
c. ^{C}ADA- DBCO	14.4	1185.93	9.2	993.81

a.

i. HPLC of ^{C}ADA-Phalloidin before and after irradiation.

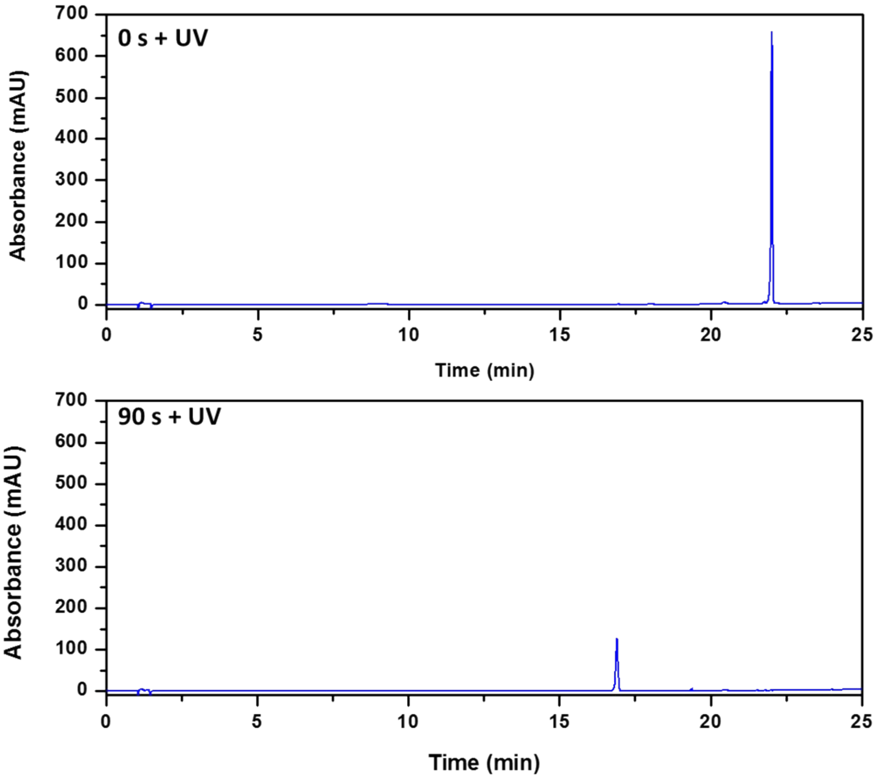

ii. LCMS of ^{C}ADA-Phalloidin before and after irradiation.

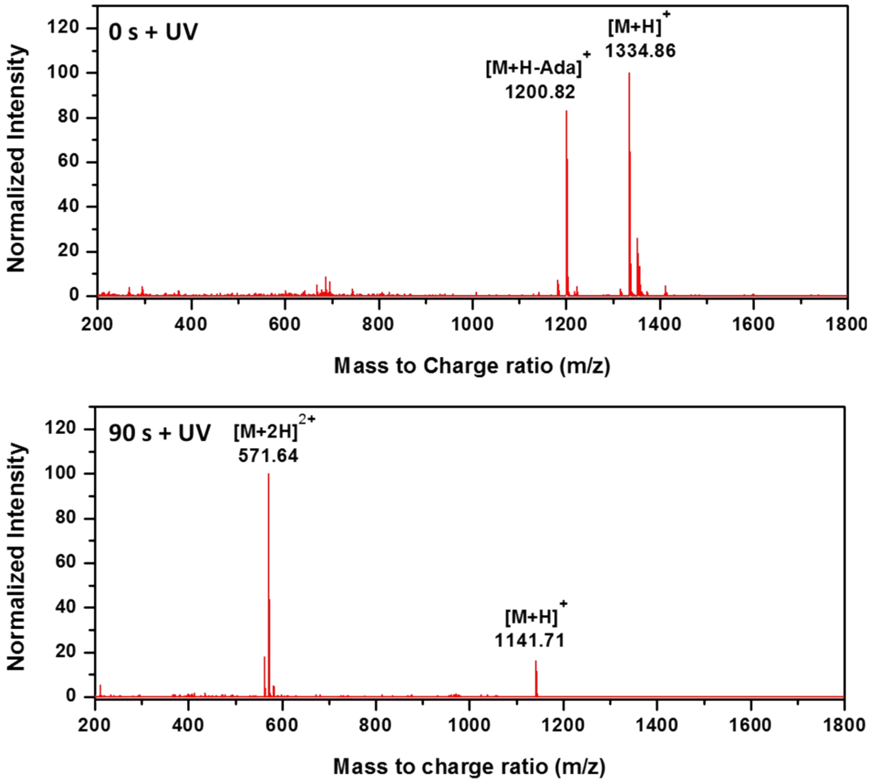

b.

i. HPLC of ^{C}ADA- SCy5 before and after irradiation.

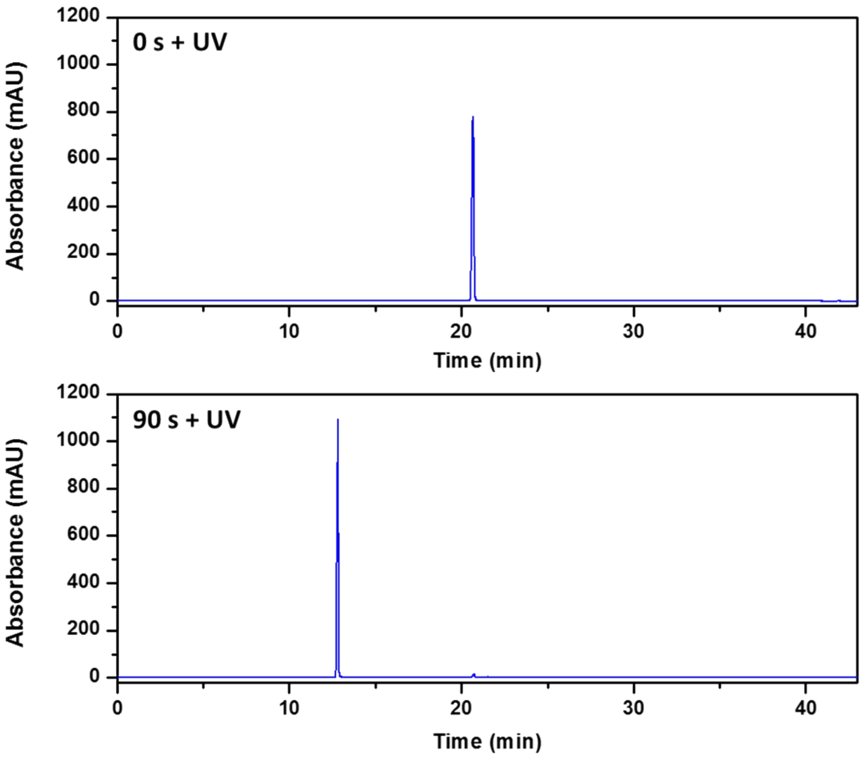

ii. LCMS of ^{C}ADA- SCy5 before and after irradiation.

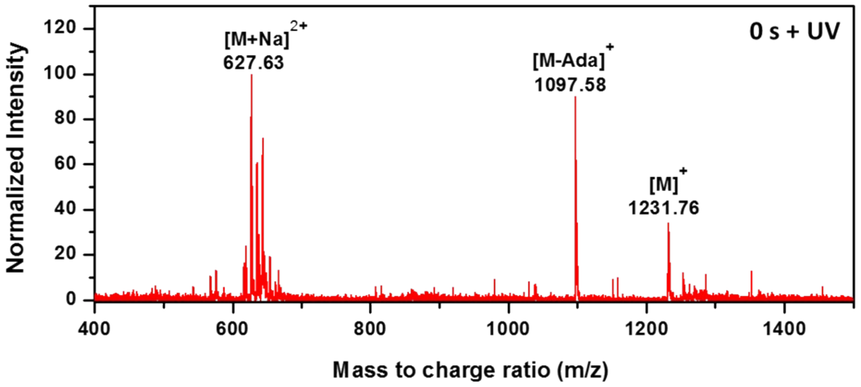

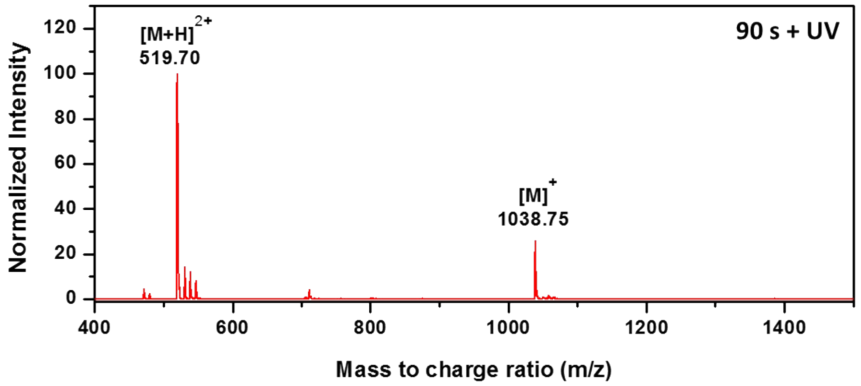

c.

i. HPLC of ^{C}ADA- DBCO before and after irradiation.

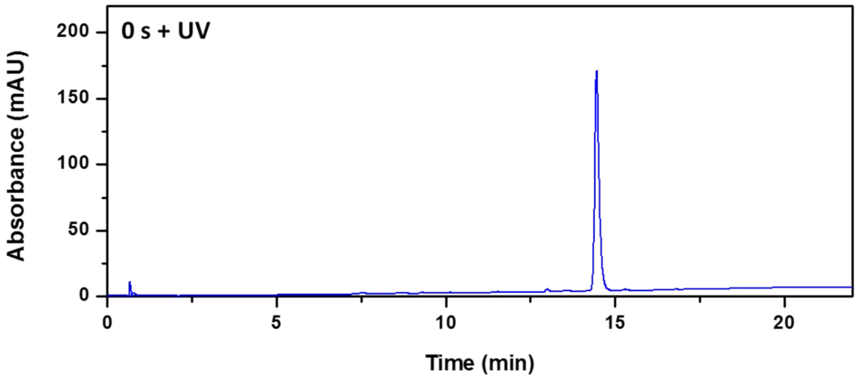

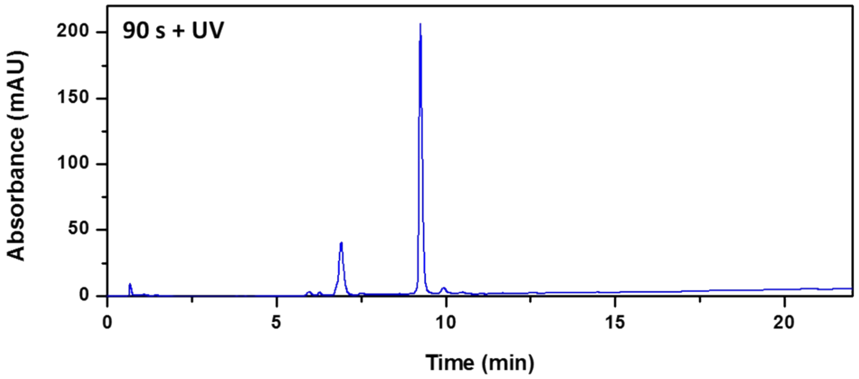

ii. LCMS of . ^{C}ADA- DBCO before and after irradiation.

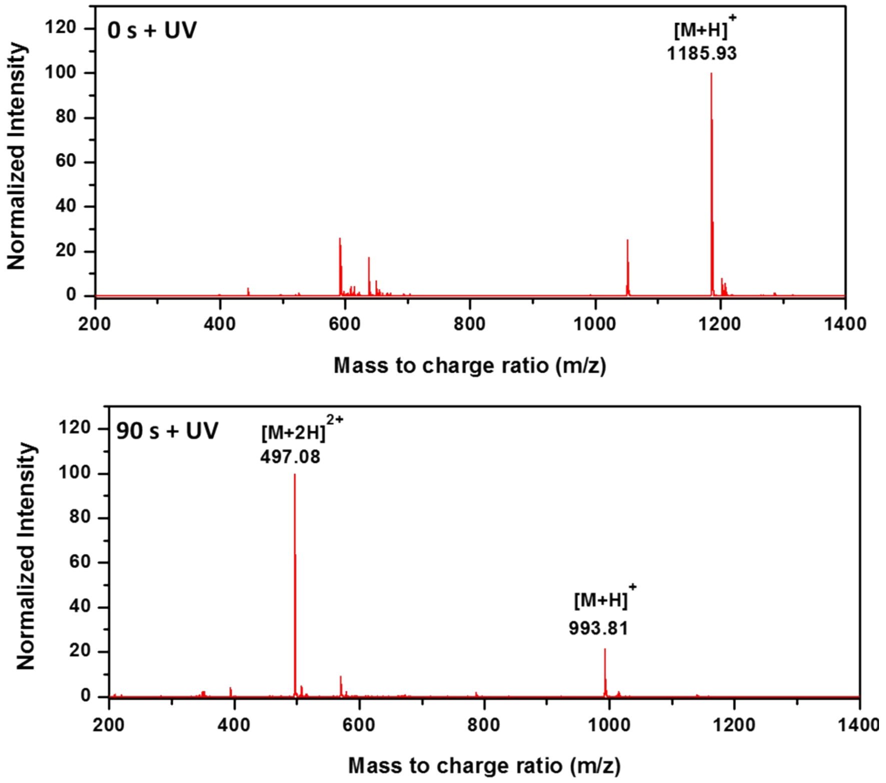

Figure S22. HPLC and LCMS chromatograms of isolated probe molecules before and after photoactivation. a) ^{C}ADA─phalloidin, b) ^{C}ADA─sCy5, and c) ^{C}ADA─DBCO in acetonitrile/water (1:1). Irradiation was performed for 90 s using 50 mW/cm^2 emitted light from a 365 nm LED light source.

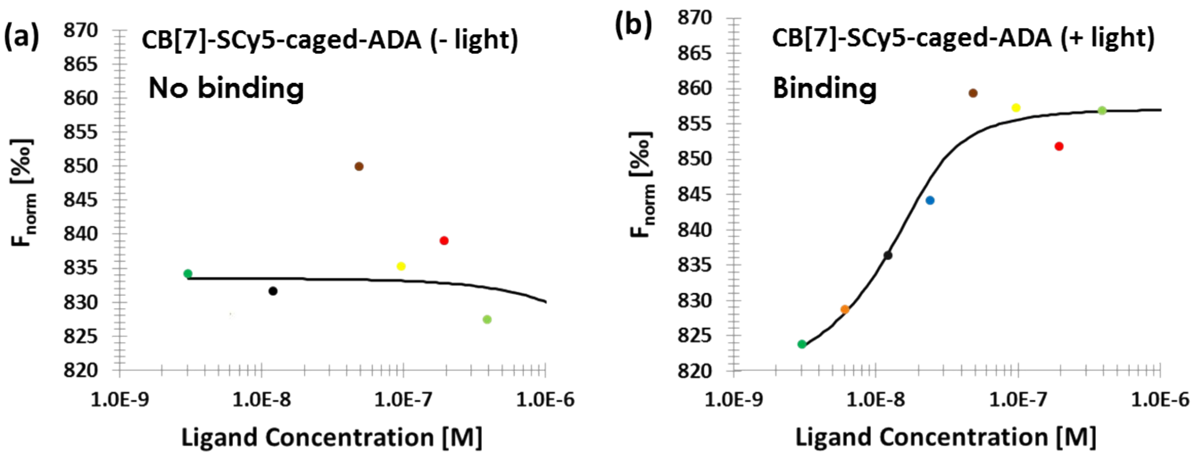

Figure S23: Microscale thermophoresis of 20 nM concentration of target ^{C}ADA─sCy5 with varying concentration of ligand CB[7] (a) Dose response chart before irradiation (b) Dose

response chart after irradiation (λ = 365 nm, 50 mW/cm^2 LED emission) for 5 min. Buffer: Water, capillary: Monolith NT.115 standard treated capillary, excitation: red (excitation wavelength: 600-650 nm), MST power: medium.

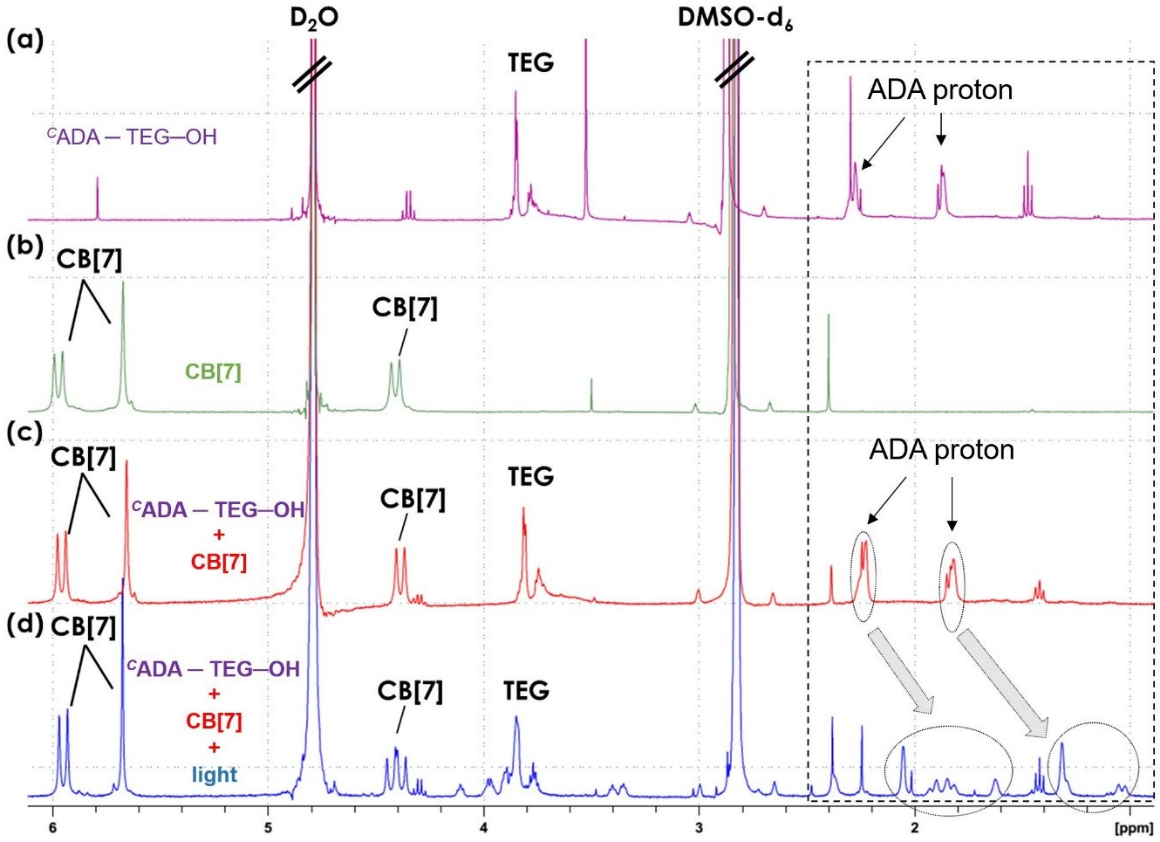

Figure S24. Overlay of ^{1}H NMR spectra of a) ^{C}ADA─TEG─OH b) CB[7] (c) CB[7]: ^{C}ADA─TEG─OH (1:1) without UV irradiated and (d) CB[7]: ^{C}ADA─TEG─OH (1:1) with UV irradiated (365 nm, 5 min, 100 mW/cm^2) in D_2O/DMSO-d$_6$ at 25°C. Concentration of all the components were kept at 5 mM.

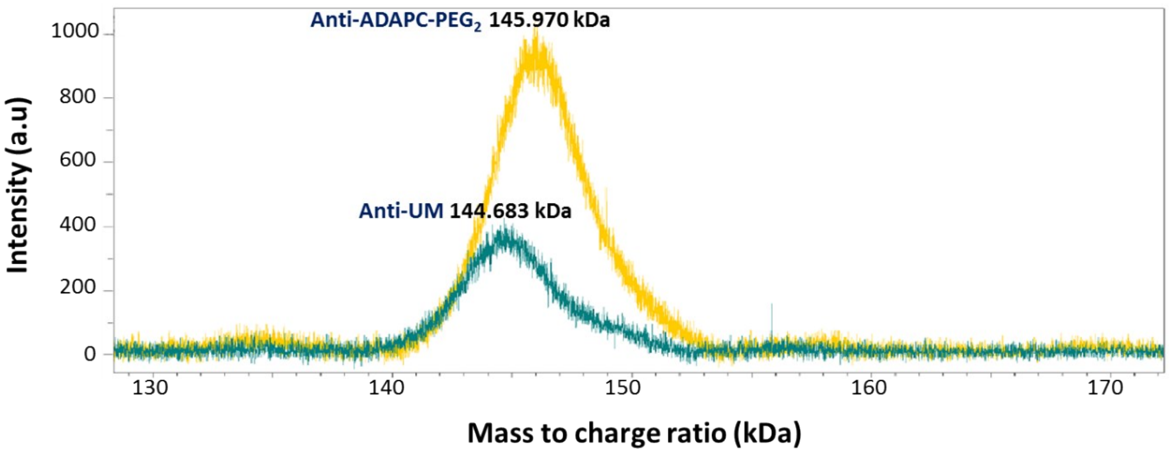

Figure S25: MALDI-TOF spectra of Donkey Anti-Rat antibody (unmodified, labelled as Anti-UM) and ^{C}ADA conjugated version of Donkey Anti-Rat antibody (modified, labelled as Anti-ADAPC-PEG$_2$). The mass difference between the two antibodies shows a 1287 Da increase in molecular weight for the modified antibody as compared to the unmodified one. The mass increase is approximately the molecular weight of a single unit of ^{C}ADA derivative that is used to modify the antibody. Expected mass increase for a single unit of ^{C}ADA–TEG–NHS-B (Compound J) incorporation is 883 Da.

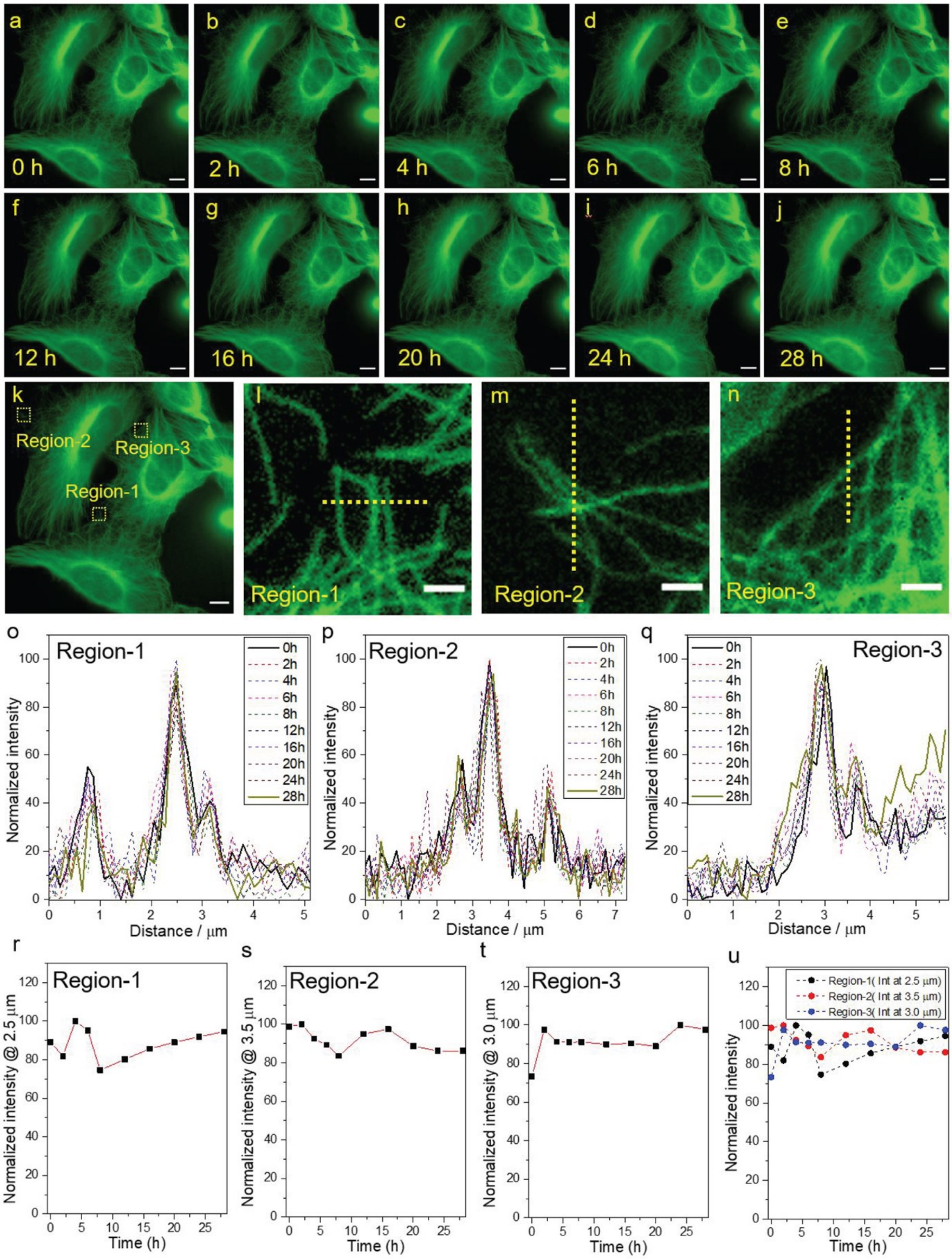

Figure S26: Long-term monitoring of photoactivated host-guest based microtubule labeling in fixed U2OS cells. (a)-(j) Fluorescence images of the same cells at different time point (0 h to 28 h). Microtubules are tagged using ^{C}ADA-antibody followed by photoirradiation (λ = 365 nm, 50 mW/cm^2 LED emission, 90 s) and labeling by CB[7]-TAMRA (100 nM). Scale

bar: 10 µm. (k) Areas selected (shown by square boxes) for drawing the intensity plot profile. Scale bar: 10 µm. (l) –(n) Zoomed images of the three selected regions which were monitored over time. Fluorescence intensity profile is drawn along the selected yellow dotted lines. Scale bar: 2 µm. (o)-(q) Intensity profile shown for three microtubule regions (Region -1,2,3), which is monitored for 28 h. The graph is plotted by subtracting the background signal from outside the cell. Therefore, the dotted line for the intensity plot is drawn from outside cellular region to the inside microtubule region. The final and initial intensity (with respect to time) is shown by solid line while the other intermediate intensity is shown in dotted line. (r)-(t) Variation in fluorescence intensity at a fixed position (maxima) over time in Region 1 (monitored at 2.5 µm of the line profile), Region 2 (monitored at 3.5 µm of the line profile) and Region 3 (monitored at 3.0 µm of the line profile). (u) Merged intensity plot of (r)-(t) for all the three regions (Region -1 in black, Region-2 in red and Region-3 in blue).

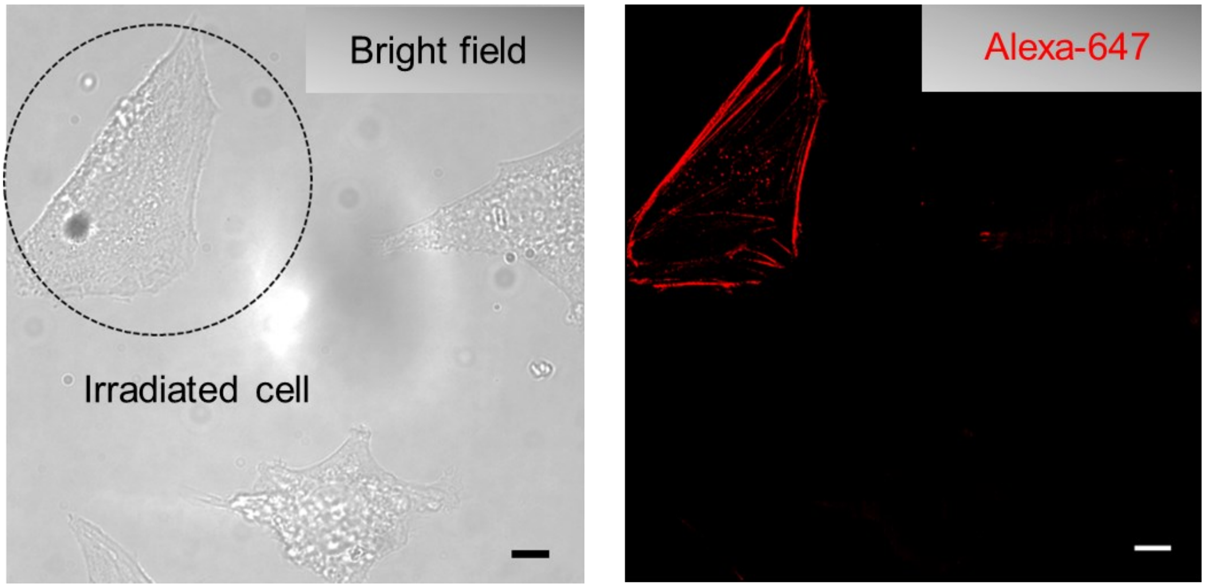

Figure S27. Spatially controlled labelling of actin. Brightfield image (left) of HeLa cell showing the targeted circular ROI selected for irradiation by 405 nm laser. SIM image (right) represents the spatiotemporal labelling of actin fibers in a single cell (in red) in Alexa-647 channel (642 nm). Scale bar: 5 µm

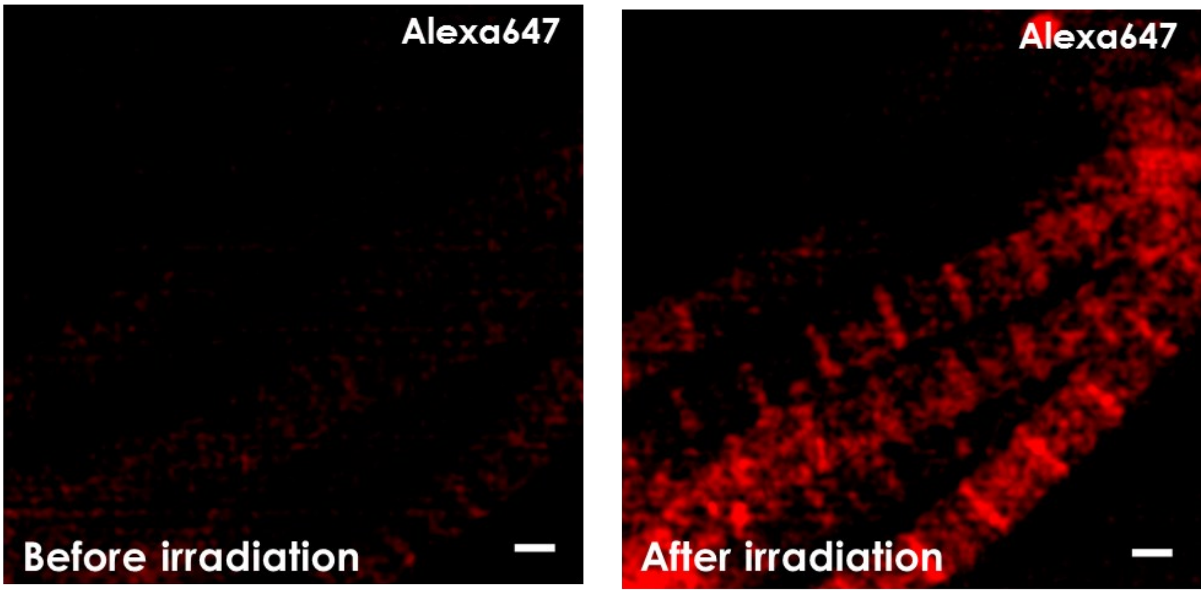

Figure S28. Photoactivated host-guest interaction mediated actin labelling in thoracic muscle tissue of *Drosophila* before and after irradiation. After irradiation shows labelling of actin in Alexa 647 channel (642 nm). Scale bar: 2 µm.

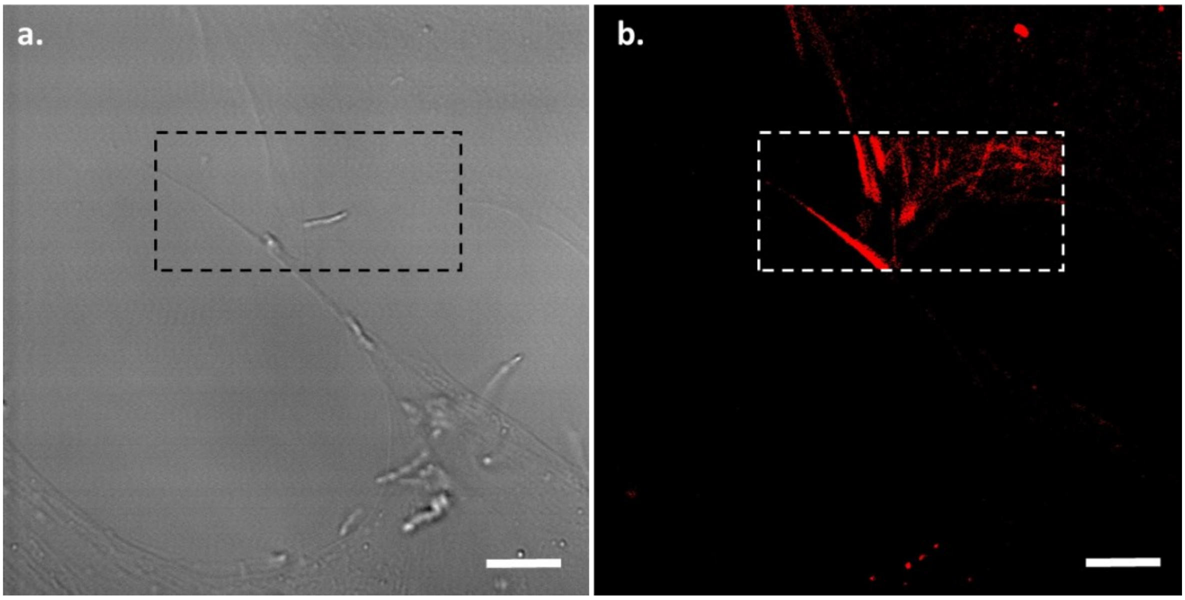

Figure S29. Activation of ^{C}ADA probe by two photon excitation using 750 nm pulsed laser. (a) Bright field image and activated region (boxed region). (b) Confocal fluorescence microscopy image after activation of probe. Scale bar: 5 µm.

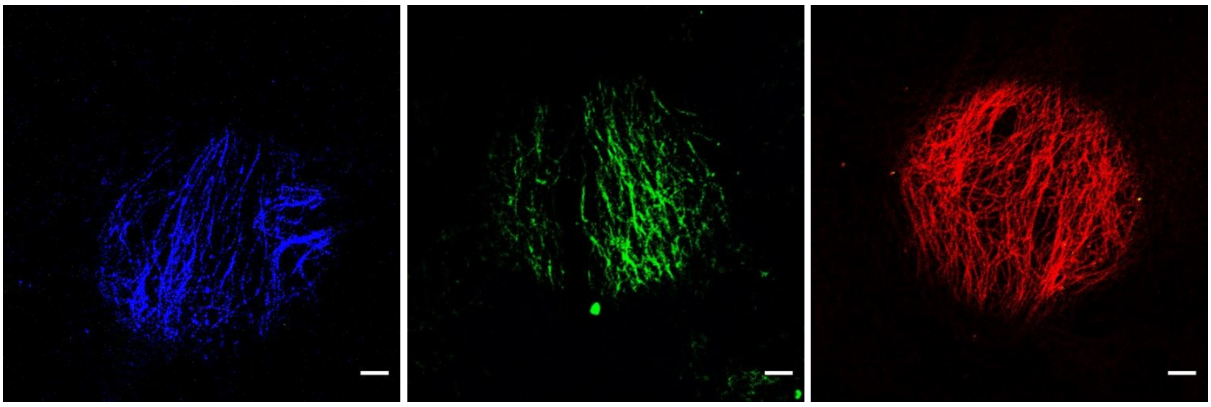

Figure S30. Magnified CLSM images of the color-coded cellular regions, clearly showing specific microtubule localization of the host-guest probe in each region. From left to right, labelling by CB[7]- fluorescein, CB[7]-TAMRA and CB[7]-Alexa647. Scale bar: 10 μm.

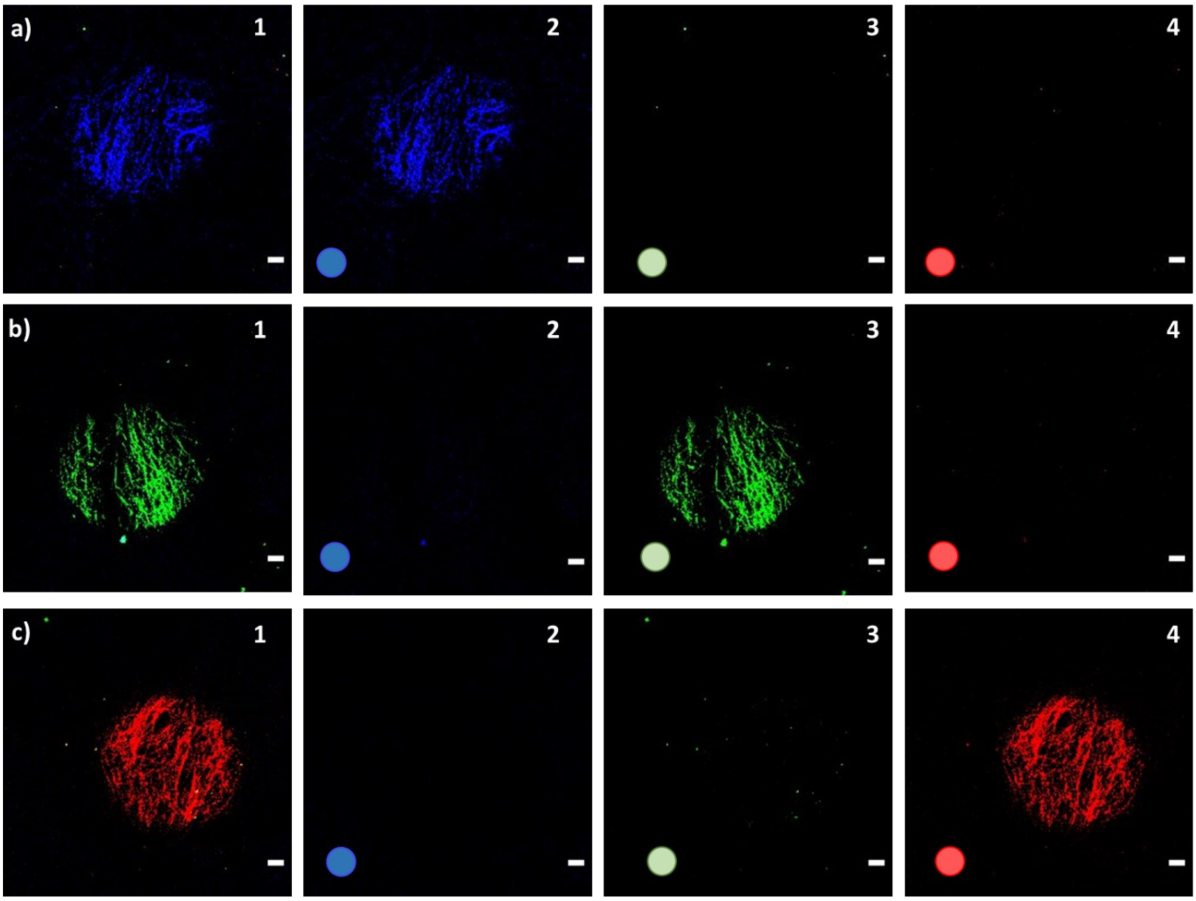

Figure S31. CLSM images of the encoded cellular barcodes (using single fluorophores in each cycle). Different fluorophore channels are individually shown from the image that is acquired after complete cycle of colour coding. This clearly indicates the negligible colour mixing between each cycle. a) CB[7]─ fluorescein b) CB[7]─TAMRA c) CB[7]─Alexa647.

1 - merged channel, 2 - 488 nm channel, 3 – 561 nm channel and 4 – 633 nm channel. The images were acquired after the ROI-3 activation. Scale bar: 10 µm.

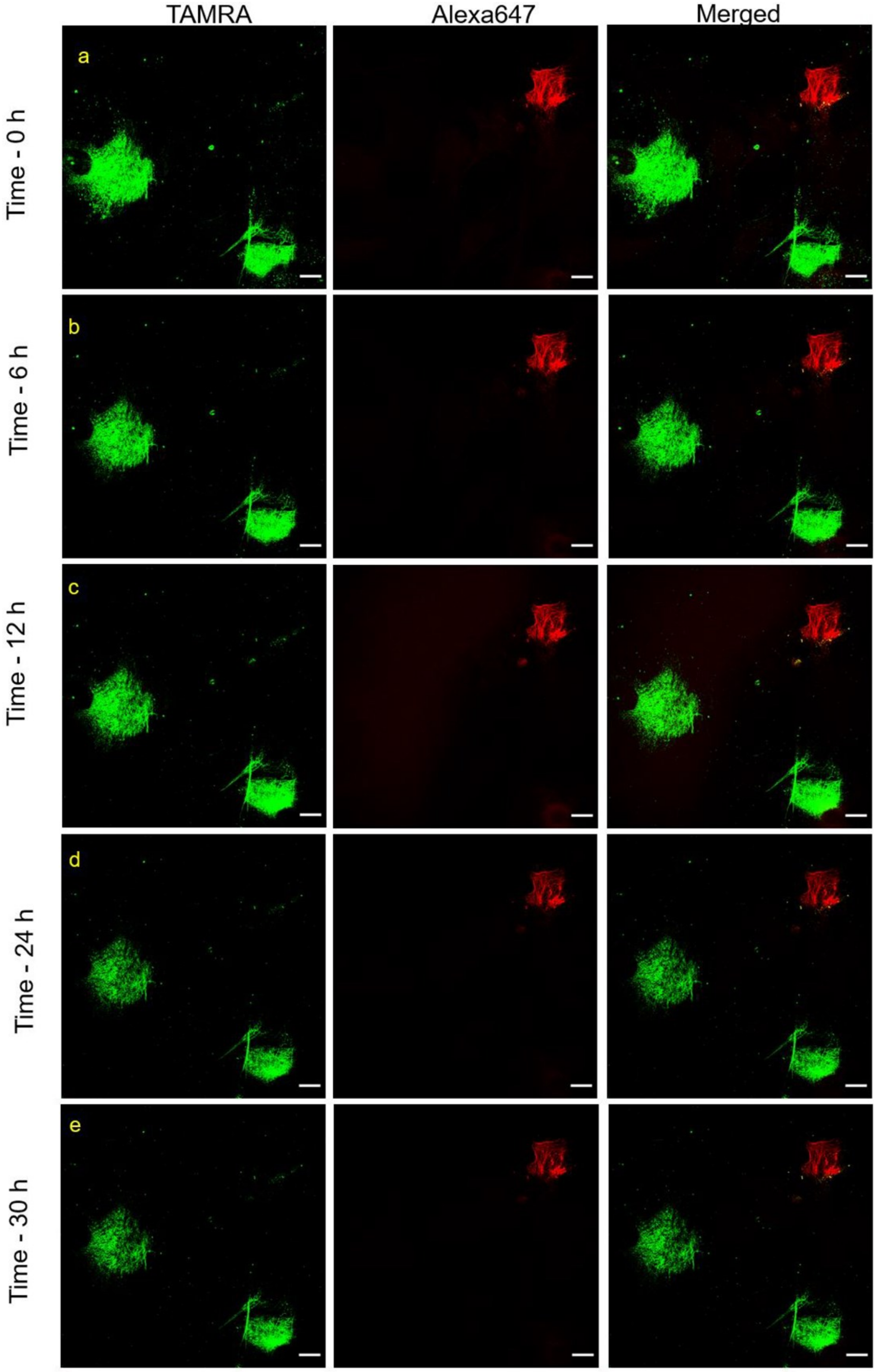

Figure S32: Time lapse imaging of two-color barcodes in MEF cell. (a)-(e) Monitoring of two-color barcodes for a time-interval of 30 h in fixed MEF cells. Three microtubule regions are optically coded with CB[7]-TAMRA (2 regions) and CB[7]-Alexa 647 (1 region). CLSM

images were presented in same brightness contrast throughout the time-interval. Both the channels (Alexa647/Red channel and TAMRA/Green channel) show no mixing of the respective colors over a time period of 30 h. Scale bar: 20 µm.

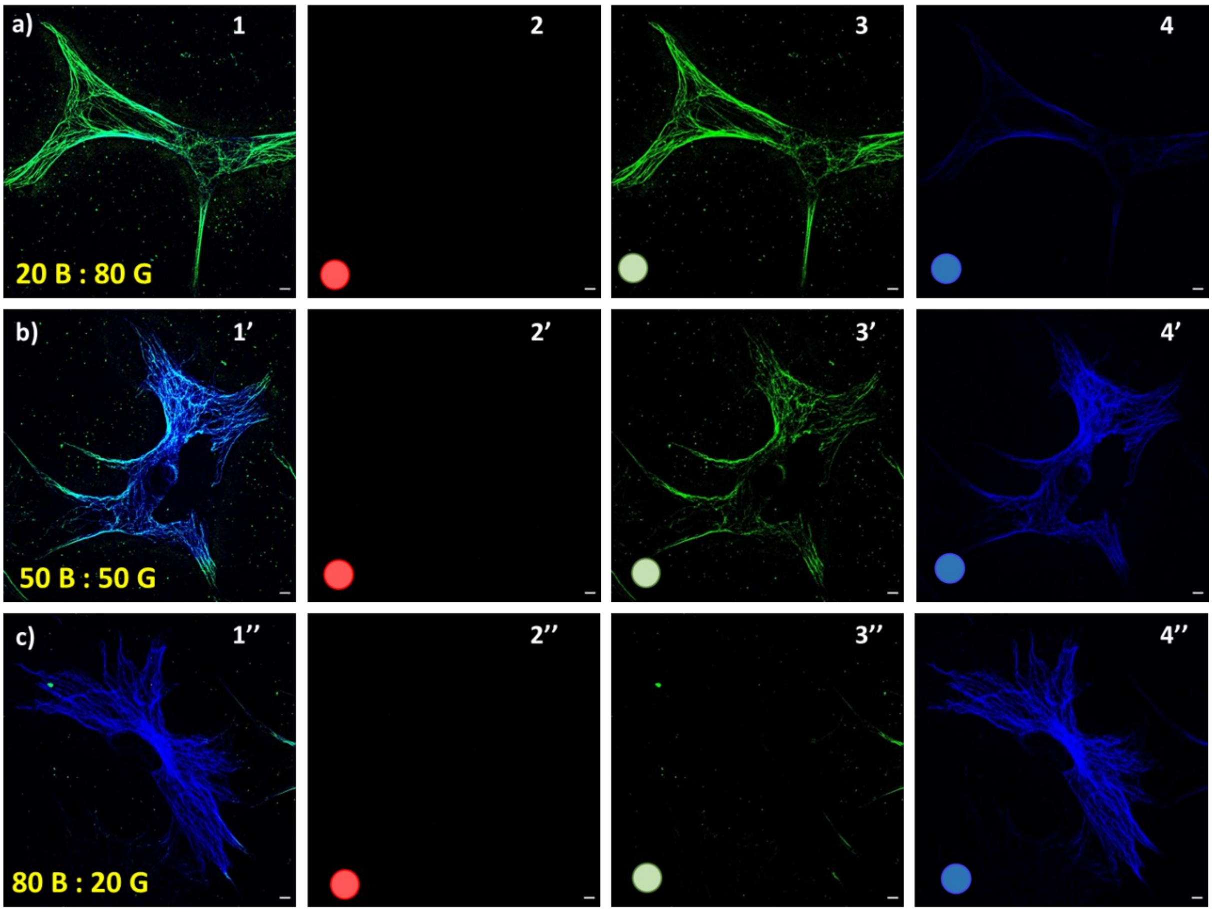

Figure S33. Barcoding cells with different mixtures of CB[7]─fluorescein (B) and CB[7]─TAMRA (G). a) 20%CB[7]─ fluorescein /80%CB[7]─TAMRA, b) 50%CB[7]─fluorescein/50%CB[7]─TAMRA, and c) 80%CB[7]─ fluorescein/20%CB[7]─TAMRA, where 1 - merged channel, 2 - 488 nm channel, 3 – 561 nm channel and 4 – 633 nm channel. The images are acquired after the ROI-3 activation. Scale bar: 10 µm.

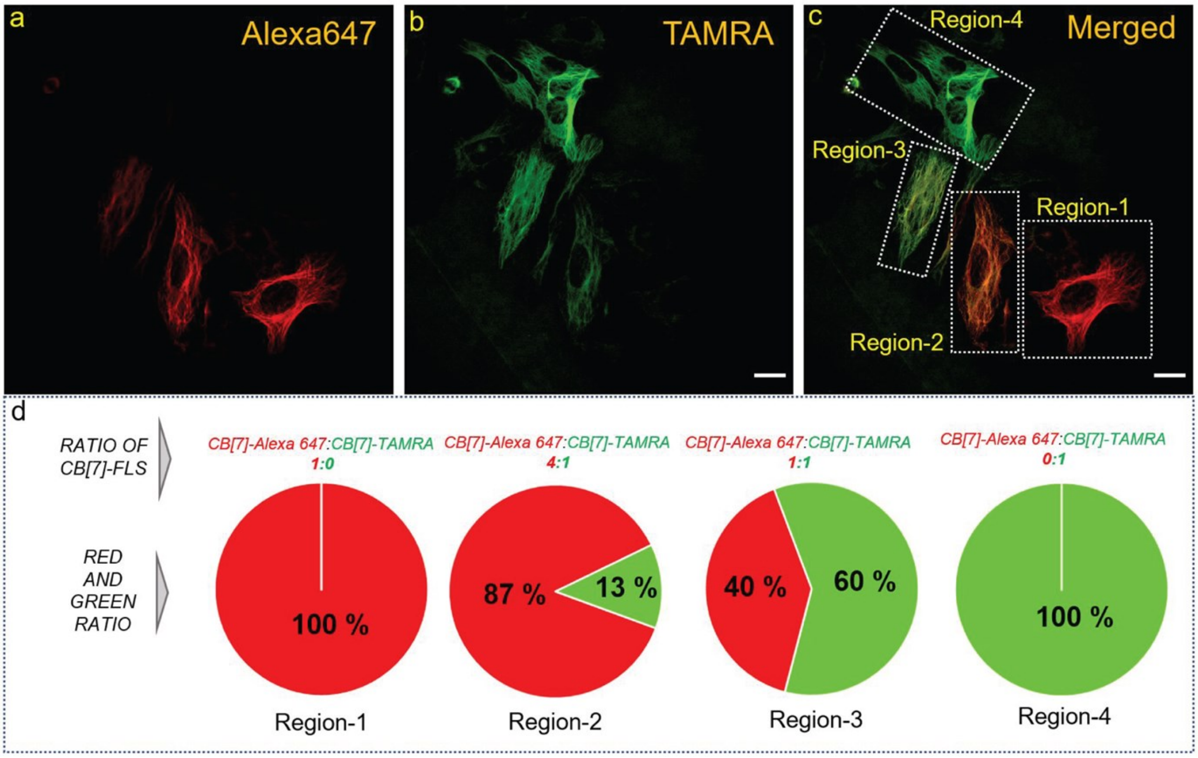

Figure S34: Barcoding cells with different ratio of CB[7]─Alexa 647 (Red) and CB[7]─TAMRA (Green). Microtubules are tagged using ^{C}ADA-antibody followed by photoirradiation and subsequent cycle of fluorophore incubation. Region-1 (CB[7]-Alexa647: CB[7]-TAMRA-1:0), Region-2 (CB[7]-Alexa647:CB[7]-TAMRA-4:1), Region-3 (CB[7]-Alexa647:CB[7]-TAMRA-1:1) and Region-4 (CB[7]-Alexa647:CB[7]-TAMRA-0:1) are irradiated in four cycles, with each cycle consists of incubation with respective ratio of fluorophores. (a) Alexa 647 channel (b) TAMRA channel and (c) Merged image. (d) Relative green and red fluorescence ratio of each barcodes. ROI manager in ImageJ (Fiji) was used to calculate the mean intensity in each region of interest (ROI) in red and green channel. The mean intensity obtained in each channel was normalized after subtracting the background intensity (from outside the cell) for each channel. Final ratio between green and red fluorescence colours were assigned by normalizing against single CB[7]-FL labelled region (i.e., 1:0 or 0:1 ratio). Scale bar: 20 µm

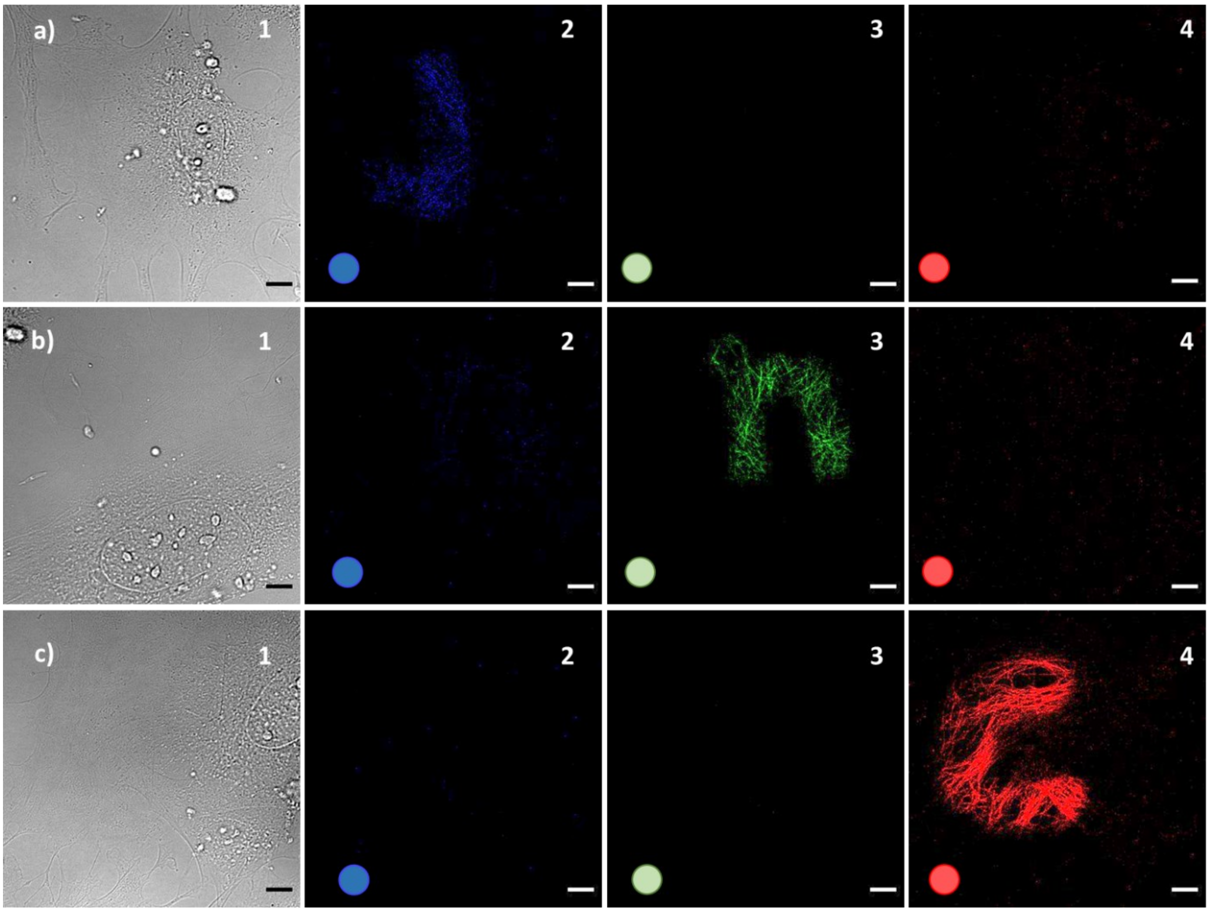

Figure S35. Barcoding with an alphabetical shape (using single fluorophores in each cycle). Different fluorophore channels are individually shown from the image that is acquired after complete cycle of colour coding. This clearly indicates the negligible colour mixing between each cycle. a) CB[7]─fluorescein b) CB[7]─TAMRA c) CB[7]─Alexa647, where 1 - merged channel, 2 - 488 nm channel, 3 – 561 nm channel and 4 – 633 nm channel. The images are acquired after the ROI-3 activation. Scale bar: 10 µm.

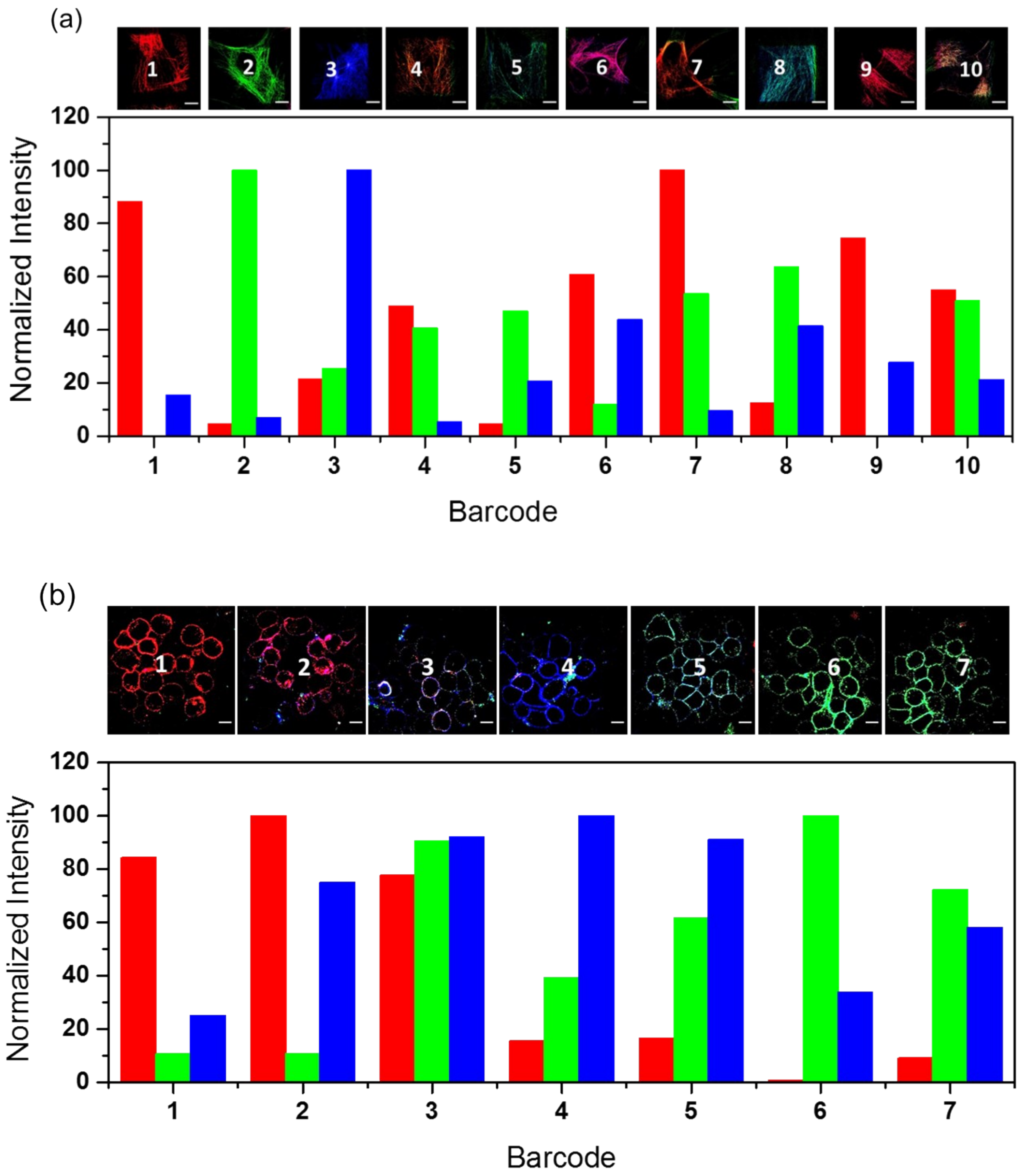

Figure S36. Relative emission intensities of barcoded regions in (a) fixed MEF cells, containing microtubule targeted ^{C}ADA and (b) live MCF7 cells, containing glycan targeted ^{C}ADA. All the barcoded regions are shown with serial numbers with respect to the given plots. Blue: CB[7]-fluorescein , Green: CB[7]-TAMRA and Red: CB[7]-Alexa 647. Scale bar: 10 μm.

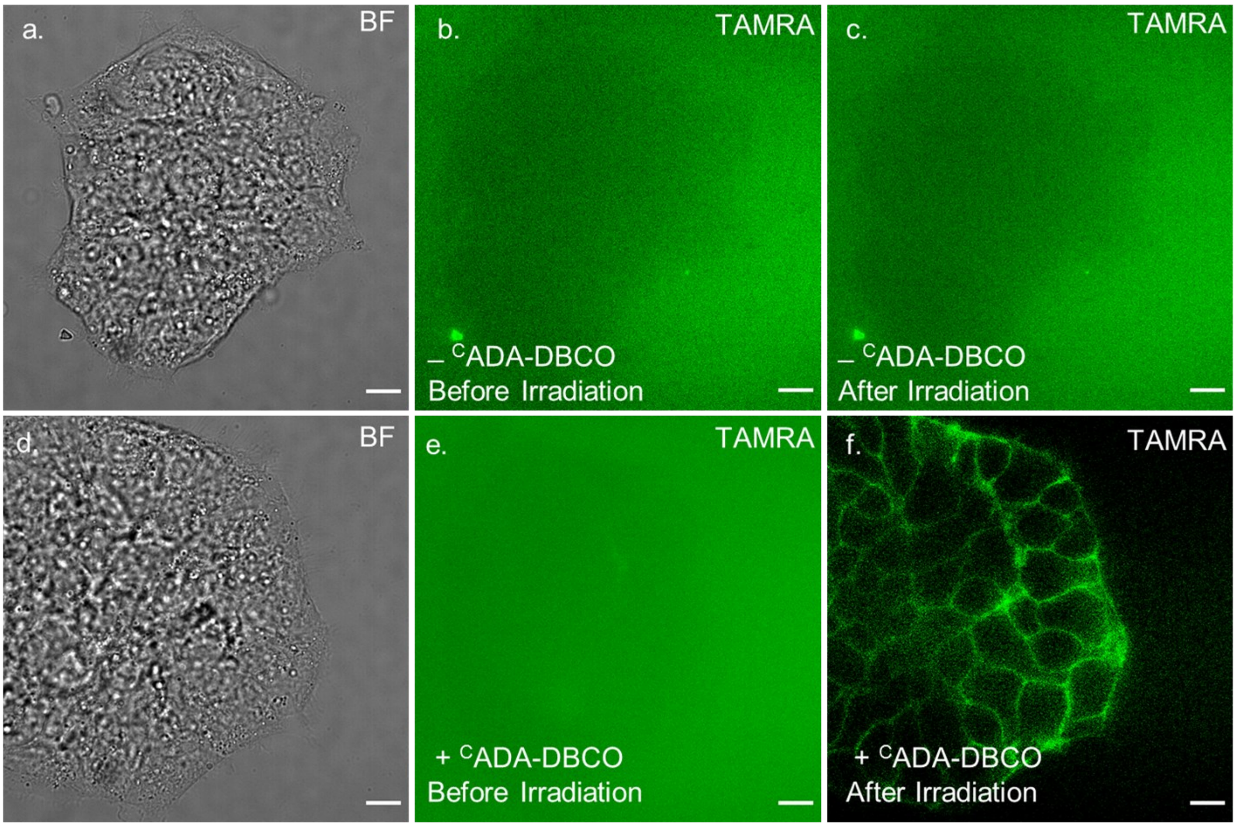

Figure S37. Control experiments for live cell glycan labelling. (a)-(c) Cells were incubated with $Ac_4ManNAz$ (25.0 µM, 72 h) and imaged with CB[7]-TAMRA (50 nM, without ^{C}ADA-DBCO). (a) Brightfield image of HEK cells (b) Before irradiation image in TAMRA channel (c) After irradiation image in TAMRA channel. (d)-(f) Cells were incubated with $Ac_4ManNAz$ (25.0 µM, 72 h) followed by ^{C}ADA-DBCO incubation (25.0 µM, 2 h) and imaged with CB[7]-TAMRA (50 nM). (d) Brightfield image of HEK cells (e) Before irradiation image in TAMRA channel (f) After irradiation image in TAMRA channel. Irradiation was performed for 90 s using 50 mW/cm^2 emitted light from a 365 nm LED light source. Scale bar – 10 µm.

References:

1. S.-G. Sampathkumar, A. V Li and K. J. Yarema, *Nat. Protoc.,* 2006, **1**, 2377–2385.

2. R. Sasmal, N. Das Saha, M. Pahwa, S. Rao, D. Joshi, M. S. Inamdar, V. Sheeba and S. S. Agasti, *Anal. Chem.,* 2018, **90**, 11305–11314.

3. W. L. Jorgensen, J. Chandrasekhar, J. D. Madura, R. W. Impey and M. L. Klein, *J. Chem. Phys.,* 1983, **79**, 926-935.

4. S. Kim, J. Lee, S. Jo, C. L. Brooks, H. S. Lee and W. Im, *J. Comput. Chem.,* 2017, **38**, 1879– 1886.

5. J. Wang, W. Wang, P. A. Kollman and D. A. Case, *J. Mol. Graph. Model.,* 2006, **25**, 247-260.

6. M. J. Abraham, T. Murtola, R. Schulz, S. Páll, J. C. Smith, B. Hess and E. Lindah, *SoftwareX,* 2015, **1–2**, 19-25.

7. G. Bussi, D. Donadio and M. Parrinello, *J. Chem. Phys.,* 2007, **126**, 014101, DOI 10.1063/1.2408420.

8. M. Parrinello and A. Rahman, *J. Appl. Phys.,* 1981, **52**, 7182-7190.

9. S. Kumar, J. M. Rosenberg, D. Bouzida, R. H. Swendsen and P. A. Kollman, *J. Comput. Chem.,* 1992, **13**, 1011–1021.
